# Global, regional and national burden of childhood sexual abuse and bullying in adolescents and young adults: a Global Burden of Disease 2021 analysis

**DOI:** 10.3389/fpsyt.2025.1679479

**Published:** 2025-10-30

**Authors:** Derong Lin, Tong Yin, Zhuangtang Shi, Xiaohua Xie, Jingya Fang, Mei Li, Yue Li, Shuxiong Luo, Aiguo Xue, Jingrong Liang

**Affiliations:** ^1^ Dongguan Hospital of Guangzhou University of Chinese Medicine, Dongguan, China; ^2^ The Second Affiliated Hospital of Guangzhou University of Chinese Medicine, Guangzhou, China; ^3^ Zhuhai Chronic Disease Prevention and Treatment Centre, Zhuhai, China

**Keywords:** child abuse, sexual, bullying, adolescent, young adult, global health, disability-adjusted life years

## Abstract

**Objective:**

Childhood sexual abuse and bullying (CSA/B) undermine adolescent and young-adult health worldwide. We quantified CSA/B-attributable mortality and disability globally across 204 countries and territories (1990–2021), with regional and national disaggregation.

**Methods:**

We analysed the GBD 2021 dataset and applied the comparative risk assessment framework to estimate CSA/B-attributable deaths and disability-adjusted life years (DALYs) by sex, age and Sociodemographic Index (SDI), summarising temporal trends using estimated annual percentage change (EAPC).

**Results:**

CSA/B-related deaths fell from 260 to 187 (–28 %), whereas DALYs rose from 2.54 million to 3.69 million (+45 %). The age-standardised mortality rate declined (EAPC –2.4 %), but the age-standardised DALY rate grew slightly (EAPC +0.5 %). Males carried higher absolute counts, yet females showed steeper DALY growth (+51 % *vs* +40 %). High-middle and middle-SDI regions achieved the greatest mortality reductions; deaths climbed 103 % and DALY rates 220 % in low-SDI areas. Anxiety accounted for most disability at 15–19 years, whereas depressive and alcohol-use disorders predominated at 20–24 years. Regionally, South Asia led deaths/DALYs, Australasia was lowest; Age-standardised mortality rates (ASMR) peaked in Eastern Europe, age-standardised DALY rates (ASDR) in high-income North America; DALYs rose fastest in Western/Central/Eastern sub-Saharan Africa.

**Conclusions:**

Falling mortality alongside expanding disability reveals a widening survivorship gap driven by mental ill-health, particularly among young women in resource-poor settings. Age-specific, gender-responsive violence-prevention and mental-health services are urgently needed to stem the growing DALY burden and advance global AYA wellbeing.

## Introduction

Childhood sexual abuse (CSA) and bullying victimisation (BV) are common, harmful exposures with enduring mental-health consequences from early adolescence through young adulthood. Recent global syntheses indicate substantial lifetime and recent exposure to sexual violence in youth and marked impairment in mental health across ages 10–24 years, underscoring the policy salience of these risks (1, 2). Within the Global Burden of Disease (GBD) 2021 framework, CSA and BV are recognised risk factors linked to depressive and anxiety disorders and alcohol use disorder in adolescents and young adults (AYA), enabling comparative assessment across 204 countries and territories (3).

To improve narrative coherence and avoid implying country-specific emphasis in a global analysis, we removed earlier single-country examples and instead reference multi-region evidence. Cross-national data show heterogeneity by context and survey system, as well as consistently higher reported exposure among girls/young women, which reflects both differential victimisation and differential disclosure (4). These patterns motivate stratified analyses by sex and sociodemographic development.

AYA are particularly susceptible to the sequelae of peer- and adult-perpetrated violence due to developmental sensitivity to social evaluation and peer influence, interacting with rapid neurocognitive change and context. Contemporary neurodevelopmental reviews highlight normative increases in sensitivity to peer feedback and social information processing during adolescence, which can amplify the mental-health impact of adverse interpersonal experiences such as bullying and sexual victimisation (5, 6).

Against this background, our aim is to quantify the global, regional, and national burden of CSA/B in AYA using GBD 2021 risk-outcome pairs, disaggregating disability-adjusted life years (DALYs) into years lived with disability (YLD) and years of life lost (YLL) and stratifying by age, sex, and sociodemographic index (SDI). This design aligns with evidence that, despite declines in mortality among youth, the non-fatal burden from mental disorders predominates and is rising in many settings, making explicit YLD–YLL presentation essential for interpretation (7).

Finally, we use standard GBD 2021 comparative risk assessment methods and reporting standards to enhance transparency and comparability across locations and time.

Abbreviations: adolescents and young adults (AYA); childhood sexual abuse and bullying (CSA/B); disability-adjusted life years (DALYs); years of life lost (YLLs); years lived with disability (YLDs); Sociodemographic Index (SDI); estimated annual percentage change (EAPC).

## Methods

### Data sources

We performed a secondary analysis of the Global Burden of Disease 2021 dataset covering 204 countries and territories, 371 diseases and injuries, and 88 risk factors for the period 1990–2021 (8). The GBD comparative risk assessment hierarchy includes 631 risk–outcome pairs synthesised from 54,561 distinct data sources (3, 9). Raw data were retrieved from the Institute for Health Metrics and Evaluation Global Health Data Exchange (IHME–GHDx). Exposure distributions by age, sex, region, and year were estimated using spatiotemporal Gaussian process regression (ST–GPR) or DisMod–MR 2.1, while exposure–relative–risk (RR) curves were fitted using meta-regression—Bayesian, regularised, trimmed (MR–BRT).

### Key definitions and attribution assumptions

Within the GBD comparative risk framework, CSA was defined as any unwanted contact sexual act experienced by individuals aged 15 years or younger, perpetrated by someone at least 5 years older. BV was defined as peer bullying occurring at least weekly during the past 30 days. Attributable outcomes were limited to major depressive disorder, anxiety disorder, and alcohol-use disorder, with corresponding relative risks derived from published meta-regression analyses (3). DALYs were calculated as the sum of YLL and YLD.

### Measurement heterogeneity & source mapping

We recognise heterogeneity in how CSA and bullying victimization are defined and measured across sources. Legal frameworks, survey design, recall periods, frequency thresholds, item wording, and respondent protections vary across settings (e.g., school-based HBSC/GSHS versus household VACS and administrative series) (10). Within GBD 2021, study-level adjustments (e.g., MR–BRT) were used to harmonise disparate definitions, yet residual variability likely persists, especially where data are sparse (11). Grey literature and some administrative surveillances are only partly represented in the GBD source inventory, which may yield over-/under-ascertainment (3). Accordingly, we emphasise region/SDI-level patterns over fine-grained country ranking.

### Attribution scope

Following GBD 2021, CSA/B-attributable outcomes for adolescents and young adults were limited to major depressive disorder, anxiety disorder, and alcohol-use disorder. Other plausible sequelae (e.g., PTSD, self-harm, broader substance-use disorders, selected physical conditions) fall outside the current risk-outcome set, implying conservative burden estimates (12).

### Population stratification

Analyses were restricted to adolescents and young adults aged 10–24 years, further subdivided into three 5-year age groups: 10–14, 15–19, and 20–24 years. The 204 countries and territories were stratified into five SDI quintiles (high, high-middle, middle, low-middle, and low), based on their SDI values for 2021 (13).

### Statistical analysis

Estimates were summarised by sex, age group, the 21 GBD regions, and SDI quintiles. To eliminate the confounding effect of varying population age structures, we computed ASMR and ASDR using the WHO weights:


$$ASR=\frac{\sum r_{i}w_{i}}{\sum w_{i}}$$

where $r_{i}$ represents age-specific rates, and $w_{i}$ is WHO 2000–2025 weight (3). Temporal trends in ASR were assessed via log-linear regression:


ln(ASR)=α+β(year)+ϵ

from which the estimated annual percentage change (EAPC) was derived as:


$$EAPC\;=\;100(e^{\beta }-\;1)$$

The corresponding 95% confidence intervals (CIs) were calculated as previously described (14). Spatial heterogeneity in the CSA/B burden was evaluated across the 21 GBD regions and five SDI quintiles. Associations between SDI and 2021 ASMR, ASDR, and their respective EAPCs were assessed using Spearman’s rank correlation coefficient ( ρ ):


$$\rho =1-\frac{6\sum d_{i}^{2}}{n(n^{2}-1)}$$

where $d_{i}\;$ represents rank differences, and n is the number of ranked pairs. This non-parametric approach aligns with recent GBD 2021 thematic analyses examining the relationship between sociodemographic development and disease burden (15). All statistical analyses and visualisations were conducted in R (version 4.4.2). Two-sided *P* values less than 0.05 were deemed statistically significant.

### Risk attribution parameters and uncertainty

In the GBD 2021 comparative risk assessment, the theoretical minimum-risk exposure level (TMREL) is the exposure associated with the lowest risk; for unequivocally harmful exposures such as CSA and BV, TMREL is set to zero. We quantified parameter, model and sampling uncertainty using 500 posterior draws, reporting point estimates as draw means and 95% uncertainty intervals as the 2.5th–97.5th percentiles. This specification follows GBD 2021 risk-factor methods and recent subnational implementations (3, 16). Because the CSA/B-attributable risk-outcome pairs in GBD 2021 are non-fatal mental disorders, attributable YLL is minimal; we therefore present deaths and DALYs in the main text and interpret DALYs as YLD-dominated for these outcomes.

### Ethics statement

The study used aggregated, publicly available GBD data and contained no identifiable personal information; therefore, institutional review board approval and informed consent were not required.

## Results

### Overall burden attributable to CSA/B

**Figure 1:**
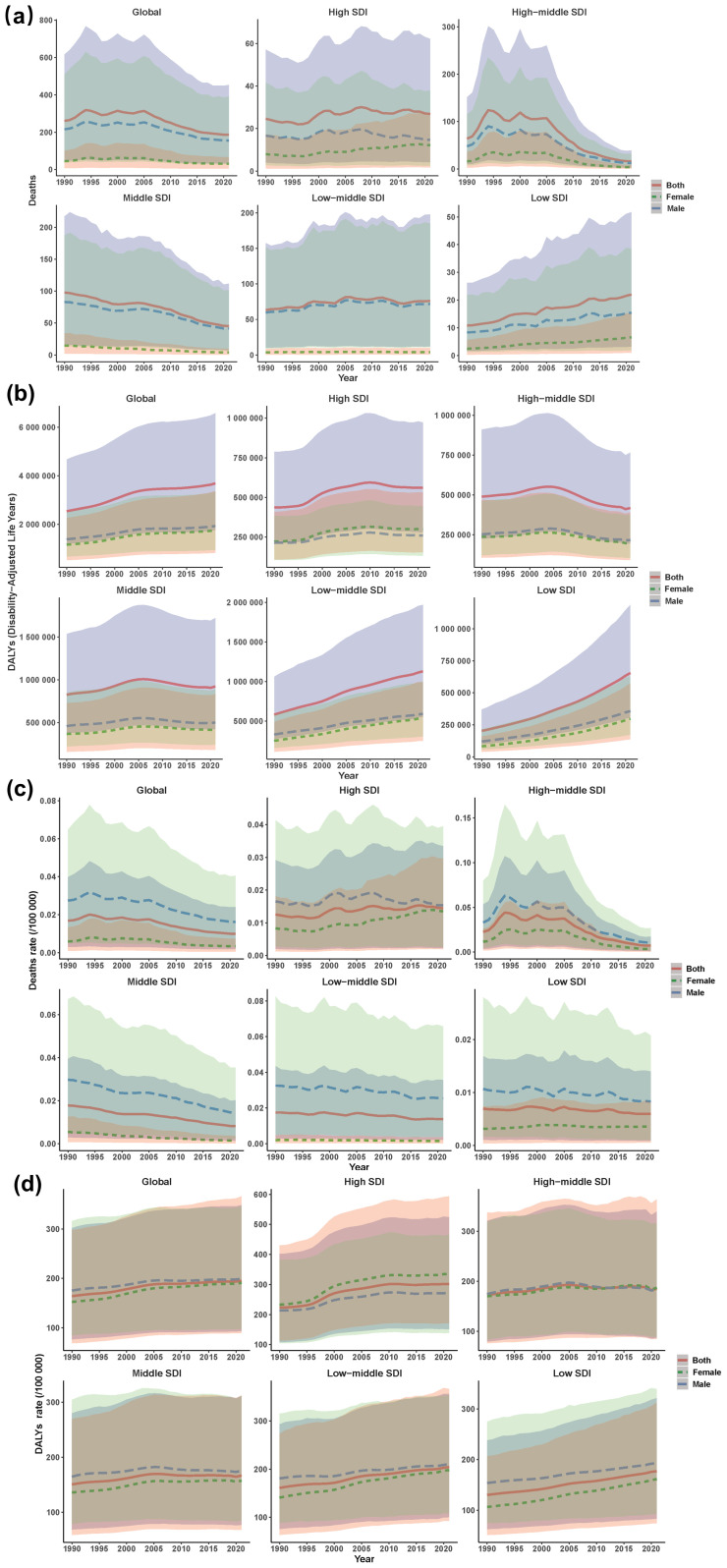
Global and SDI-level trends in CSA/B-attributable mortality and DALY burden, 1990–2021, by sex. **(a)** deaths (number); **(b)** DALYs (number); **(c)** death rate per 100 000; **(d)** DALY rate per 100 000. Colours encode sex and match legend swatches. Line style reinforces sex: red solid = both, green dashed = females, blue dashed = males. Shaded ribbons use the same hue to denote 95% uncertainty intervals. CSA/B, childhood sexual abuse and bullying; DALY, disability-adjusted life year; SDI, sociodemographic index.

Globally, the CSA/B-attributable burden among AYA between 1990 and 2021 was characterised by decreasing mortality yet increasing disability. Deaths declined by 28.3%, from 260.31 (95% UI: 45.95 to 615.84) in 1990 to 186.67 (33.69 to 453.54) in 2021. Correspondingly, the crude mortality rate decreased from 0.02 per 100,000 to 0.01 per 100,000, with an EAPC in ASMR of –2.40% (95% CI: –2.77 to –2.02; **Figure 1c**, **Supplementary Table S1**). Conversely, DALYs rose significantly by 45.2%, from 2.54 million (95% UI: 1.19 to 4.67 million) to 3.69 million (95% UI: 1.76 to 6.57 million). The global crude DALY rate increased from 164.13 to 195.28 per 100 000, with a positive ASDR EAPC of 0.54% (95% CI: 0.48 to 0.61; **Supplementary Tables S1**, **S2**, **S7**, **S8**, **Figure 1**). Consistent with the composition of DALYs in this context, the CSA/B-attributable burden among AYA was almost entirely non-fatal; increases in DALYs chiefly reflect disability rather than premature mortality.

### Regional disparities in CSA/B burden (1990–2021)

From 1990 to 2021, pronounced regional disparities in CSA/B burden emerged across the 21 GBD regions. Death counts decreased in 14 regions, with the largest declines in Eastern Europe (−78.2%), high-income Asia-Pacific (–76.7%), and East Asia (–74.6%). By contrast, Western sub-Saharan Africa recorded a sharp increase of 199.9%. DALYs increased in 16 regions, with Western, Central, and Eastern sub-Saharan Africa each showing increases exceeding 230%. In 2021, South Asia had the highest absolute number of deaths and DALYs, whereas Australasia had the lowest. Eastern Europe recorded the highest ASMR, and high-income North America had the highest ASDR (**Supplementary Tables S7**, **S8**, **Supplementary Figure S1**).

### National patterns in CSA/B burden

**Figure 2:**
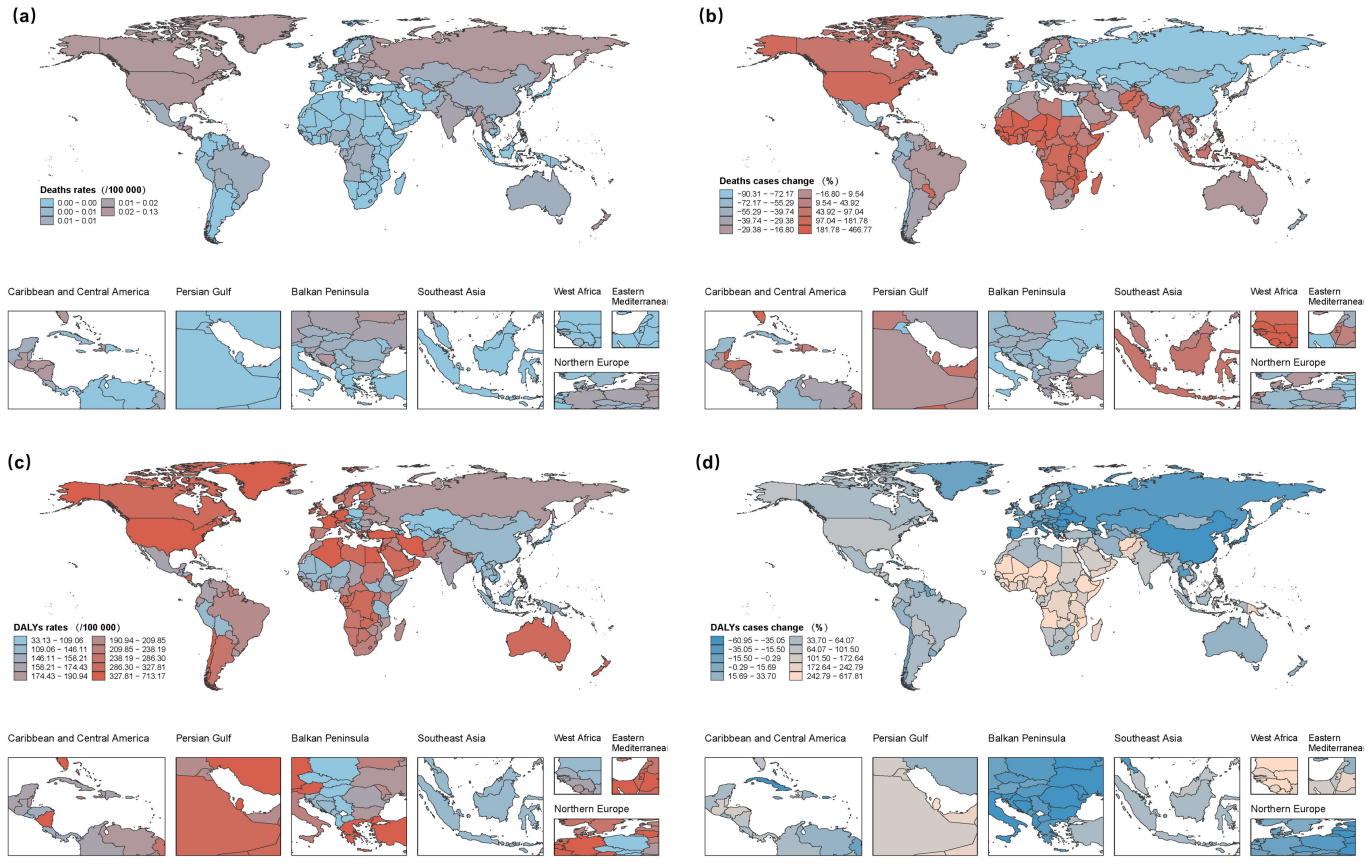
Global burden of childhood sexual abuse and bullying (CSA/B) among adolescents and young adults (10–24 years) across 204 countries and territories. **(a)** mortality rate in 2021. **(b)** Absolute change in the number of CSA/B-attributable deaths, 1990 to 2021. **(c)** DALY rate in 2021. **(d)** Absolute change in CSA/B-attributable DALYs, 1990 to 2021. DALY, disability-adjusted life year; CSA/B, childhood sexual abuse and bullying.

Among the 204 countries and territories, considerable national-level variations in CSA/B burden emerged in terms of absolute numbers, age-standardised rates, and temporal trends. In 2021, India, China, and the United States had the highest absolute death counts, whereas Greenland, Myanmar, and Guatemala exhibited the highest population-adjusted mortality rates. Chad, Cameroon, and Mali recorded the largest proportional increases in deaths from 1990 to 2021, while Egypt had the greatest decline in mortality rates, with an EAPC of –10.2% (95% CI: –11.4 to –8.9). The highest absolute DALY counts were also concentrated in India, China, and the United States, but the highest age-standardised DALY rates occurred primarily in several countries in sub-Saharan Africa (**Figures 2a–d**, **Supplementary Tables S3**–**S5**).

### Association between CSA/B burden and sociodemographic index

Between 1990 and 2021, CSA/B-attributable death counts diverged sharply by SDI quintile: high-middle and middle SDI regions saw declines of 74.3% (95% UI –83.6 to –61.1) and 53.6% (–73.5 to –22.7), respectively; high SDI remained stable (+9.3%; –17.6 to +47.6); whereas low-middle and low SDI increased by 20.2% (–40.5 to +141.8) and 103.5% (13.7 to 291.1). Age-standardised mortality rates fell across all SDI groups, most steeply in high-middle (EAPC –5.48%; –6.66 to –4.27) and middle (–2.60%; –2.75 to –2.45) regions, with smaller declines in low-middle (–1.14%; –1.29 to –0.98) and low (–0.52%; –0.69 to –0.36) SDI; high SDI reached its nadir around 2000 before rising modestly (EAPC+0.70%; 0.49 to 0.91). In contrast, DALY counts rose in all but the high SDI region (–14.6%; –22.2 to –4.5), with largest increases in middle (222.4%; 205.5 to 240.0), low (94.1%; 78.8 to 112.9), and low–middle (11.7%; 4.7 to 18.5) SDI areas. Age–standardised DALY rates increased most in high-middle (EAPC+1.09%; 0.90 to 1.28) and middle (0.96%; 0.94 to 0.98) SDI regions. By 2021, DALY rates per 100,000 formed an inverted U-shape: high-middle (301.88), low (204.24), high (185.11), middle (177.19), and low-middle (166.83) SDI (**Supplementary Tables S7**, **S8**; **Figures 1a–d**).

**Figure 3:**
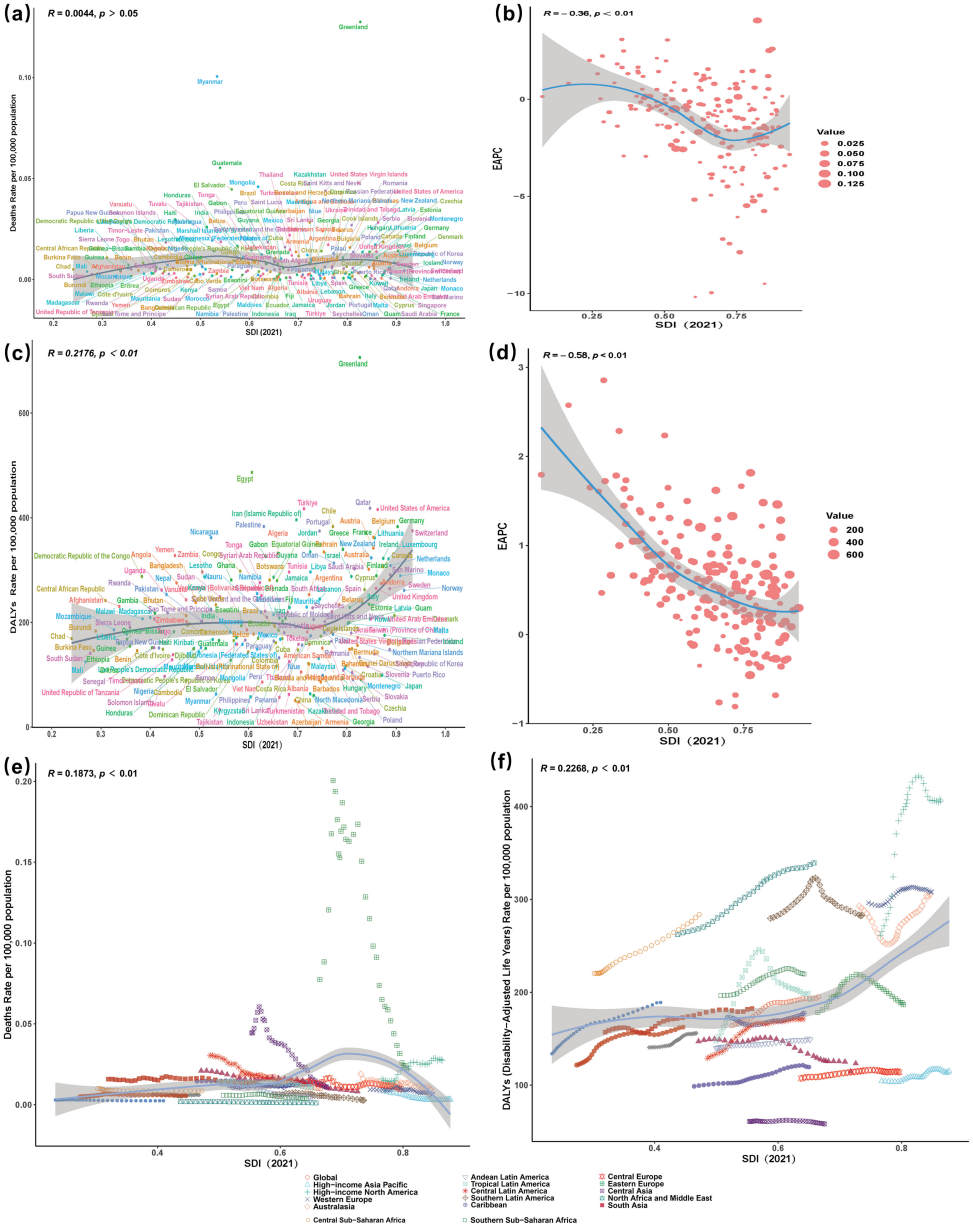
Correlations between CSA/B-related mortality and DALY rates and SDI/EAPC. **(a, c)** Associations in 2021 between SDI and mortality rate **(a)** or DALY rate **(c)**. **(b, d)** Associations in 1990–2021 between SDI and the EAPC of mortality rate **(b)** or the EAPC of DALY rate **(d)**. **(e, f)** SDI–mortality and SDI–DALY trajectories across 21 GBD regions, 1990–2021; points from left to right denote successive years and the solid line shows the expected value. CSA/B, childhood sexual abuse and bullying; SDI, sociodemographic index; DALY, disability-adjusted life year; EAPC, estimated annual percentage change.

Spearman correlation analysis revealed no significant association between SDI and CSA/B-related mortality rates globally (*p*>0.05), whereas a weak but significant positive correlation was identified between SDI and DALY rates (*p*<0.01). Several high-SDI/high-income countries—notably Greenland and Qatar—showed higher-than-expected DALY rates for their SDI levels, whereas Tajikistan and Armenia were markedly lower (**Figures 3a, c**). Negative correlations between SDI and the EAPC for mortality and DALYs indicated faster burden growth in low SDI countries, though a few middle SDI countries, such as Azerbaijan and Mongolia, diverged from this general pattern (**Figures 3b, d**).

Additionally, mortality rates in Tropical Latin America have been consistently higher than expected since 1990. With the exception of High-income North America—where age-standardised rates increase as SDI rises—the vast majority of regions exhibit a declining trend with increasing SDI (**Figure 3e**). Furthermore, DALYs exhibited a weak positive correlation with the SDI (*p* < 0.001); however, the curve’s shape reveals a plateau or modest decline in DALY rates across low- to middle-SDI settings, whereas at high SDI levels—particularly in high-income North America and Western Europe—an inflection point is followed by a renewed rise in DALYs (**Figure 3f**).

### Sex-specific differences in CSA/B burden

Between 1990 and 2021, mortality decreased by 27.6% among males and 31.5% among females, yet males consistently accounted for around five times more deaths than females in 2021. By contrast, DALYs increased in both sexes, with females exhibiting a notably greater relative increase (+51.4%, ASDR+25.2%) compared to males (+39.9%, ASDR+13.7%), a difference statistically significant (*p*<0.001). Analysis stratified by SDI indicated substantial decreases in deaths and DALYs within high-middle SDI regions, whereas low SDI regions demonstrated substantial increases in female deaths (+168.7%) and DALYs (+260.1%), highlighting profound gender disparities (**Figures 1a, b**, **Supplementary Tables S1**, **S2**).

### Age-specific trends in CSA/B burden

In 2021, CSA/B-attributable deaths and DALYs increased progressively with age, peaking in the 20–24 years age group. Anxiety disorders dominated the DALY burden among those aged 10–14 years (61%), whereas depressive disorders became predominant among 15–19-year-olds (≈52%). By the age of 20–24 years, depressive disorders further increased to approximately 57%, alongside a notable emergence of alcohol-use disorders (5%). This age-related shift represents a transition from anxiety-dominated disability in early adolescence to depression and substance-use disorders in late adolescence and young adulthood (**Supplementary Figure S2**, **Supplementary Tables S6**).

## Discussion

Using the most recent GBD 2021 dataset, we comprehensively assess global, regional, and national burdens attributable to CSA/B among adolescents and young adults aged 10–24 years. Between 1990 and 2021, we found substantial declines (~28%) in CSA/B-related mortality, accompanied by a marked (~45%) increase in DALYs, indicating a shifting burden from fatal outcomes toward chronic mental health sequelae. Sex and age-specific disparities were prominent: males consistently had higher mortality and DALY counts, whereas females demonstrated a greater relative rise in DALYs. Moreover, the CSA/B burden escalated significantly with age, peaking among youth aged 20–24 years, transitioning from anxiety disorders prevalent at younger ages (10–14 years) to depressive and substance-use disorders dominating in older adolescents and young adults (20–24 years).

Marked disparities across the SDI spectrum emerged in our analysis, revealing substantial inequities. High-middle and middle SDI regions experienced pronounced mortality reductions alongside moderate declines in DALYs, whereas low-middle and low SDI regions saw marked increases in both deaths and DALYs—most notably in low SDI areas, where female DALYs more than doubled. This pronounced SDI gradient likely reflects variation in legislative enforcement, digital safeguards against bullying, availability of mental health services, and implementation of preventive interventions. The inverted U-shaped distribution of crude DALY rates by SDI quintile underscores the multifaceted socioeconomic forces shaping CSA/B burdens globally.

Our findings both corroborate and extend prior prevalence-based studies of CSA/B. For instance, monthly bullying victimization was reported at 45.1% in the Eastern Mediterranean and 43.5% in Africa, compared with approximately 8.4% in Europe (17). Similarly, lifetime prevalence of sexual harassment was estimated at 11.4% globally, disproportionately affecting girls (6.8%) more than boys (3.3%) (18). Unlike these earlier investigations, our GBD-based framework integrates mortality and DALYs to quantify the broader public health impact of CSA/B exposures, thereby illuminating critical geographic and demographic disparities.

In high-SDI regions (e.g., Australasia, Western Europe, high-income Asia-Pacific), sustained declines in DALYs since 1990 are likely attributable to decreased traditional bullying, improved disclosure and reporting of sexual abuse due to heightened mental-health awareness, and the widespread implementation of mandatory reporting laws and comprehensive school-based violence-prevention programs (19). Indeed, recent meta-analyses suggest school-based interventions can reduce bullying victimisation by approximately 20% (20). Robust surveillance systems and well-resourced child protection services further contribute to early detection and timely interventions, effectively mitigating long-term mental health disability (21).

However, within high-income regions, discrepancies persist. High-income North America recorded the highest ASDR in 2021, attributable to factors such as high prevalence of early sexual violence, compounded mental-health impacts of cyber and traditional bullying, and comprehensive surveillance systems that more effectively identify cases under-reported in other regions (1, 22).

Moreover, the disproportionately rapid increase in DALYs in low-SDI regions likely reflects the chronic fragility of local health systems—marked by insufficient mental-health infrastructure, acute workforce shortages, and high out-of-pocket costs—compounded by pervasive stigma, which together amplify the observed DALY rise in the most resource-constrained settings (23, 24). Furthermore, pronouncedgaps in mental-health service coverage likely contributed to the escalation of chronic depressive and anxiety disorders among survivors, driving DALYs upward (25). By contrast, high-SDI nations have successfully implemented trauma-informed school programs and mandatory reporting frameworks, stabilising or reducing CSA/B burdens. Nonetheless, their vulnerability to socioeconomic disruptions underscores the need for sustained and adaptable preventive interventions. Additionally, the common co-occurrence of CSA/B with substance misuse and self-harm emphasises the urgency for integrated, multisectoral approaches in low-resource settings (26, 27).

At the national level, substantial variations in CSA/B burden emerged. India, China, and the United States together contributed approximately 40% of the global DALY count, reflecting large youth populations and rapid digital exposure outpacing protective regulatory mechanisms (28, 29). Conversely, sub-Saharan African countries such as Nigeria, Democratic Republic of Congo, and Tanzania exhibited among the highest age-standardised DALY rates, largely driven by widespread gender inequities and weak child protection systems (30). Small Pacific island nations (e.g., Tuvalu, Niue, Tokelau) experienced notably elevated per capita burdens, likely resulting from compounded vulnerabilities including limited mental-health infrastructure, prevalent peer violence, and stigma associated with obesity and related psychosocial stressors (7, 31, 32). Conversely, Nordic countries like Sweden maintained low burdens due to comprehensive mental-health screening, supportive parenting policies, and robust anti-bullying legislation (33, 34).

Age-specific analyses revealed progressive increases in CSA/B-related DALYs with advancing age, from early adolescence (dominated by anxiety disorders) toward young adulthood (dominated by depressive and substance-use disorders). This trajectory aligns with developmental vulnerabilities during adolescence, underscoring the critical importance of timely, targeted preventive and therapeutic interventions at different life stages (35, 36). Sex-specific disparities were also evident, with males consistently carrying a higher mortality burden, whereas females experienced a greater relative increase in DALYs over time. These differences may reflect gender-specific coping mechanisms, biological vulnerabilities to stress-related psychopathology, and gendered exposures to different forms of violence (37, 38).

### Public health implications

Our study emphasises several critical public-health imperatives. Firstly, universal implementation of evidence-based school bullying-prevention programs can significantly reduce victimisation rates (approximately 20% reduction) and thus should be widely adopted (39). Secondly, integrating timely screening within primary healthcare systems and digital platforms is essential for early identification of CSA/B survivors, reducing long-term mental health impacts (40). Thirdly, multisectoral strategies tailored to local contexts, aligned with global initiatives (e.g., INSPIRE), must be implemented. While integrated approaches exemplified by Sweden’s comprehensive parenting and anti-bullying policies serve as valuable models (41, 42), adaptation to local cultural and socioeconomic contexts remains imperative.

These actions align with the INSPIRE technical package—seven evidence-based strategies (implementation and enforcement of laws; norms and values; safe environments; parent/caregiver support; income and economic strengthening; response/support services; education and life skills)—offering a cross-sector roadmap adaptable to low-resource settings (43).

### Future research directions

Future studies should address persistent data gaps, particularly in low-resource settings, and expand understanding of causal mechanisms linking CSA/B exposures to adverse health outcomes. Further investigation into effective implementation and scaling of intervention programs is also essential, alongside longitudinal evaluations of policy impacts. Improving granularity in CSA versus bullying exposure distinctions and integrating novel digital violence into GBD assessments would further enhance global preventive efforts.

### Limitations

Our study has several limitations. Exposure ascertainment relied on heterogeneous surveys with varying definitions, recall periods, and instruments, alongside stigma-related underreporting—together likely biasing CSA/B prevalence downward (18). Source coverage is uneven (grey literature and administrative series are only partially mapped), so under-/over-ascertainment remains possible. The current GBD comparative-risk set for adolescents and young adults includes depressive, anxiety, and alcohol-use disorders only; exclusion of PTSD, self-harm, and chronic physical sequelae implies conservative DALY estimates (3). Binary exposure classifications cannot capture severity, frequency, duration, timing, or re-victimisation. Sex-specific reporting biases—particularly lower disclosure among males—may distort apparent gender gaps (44). Estimates are ecological and should not be interpreted as individual-level causal effects (45). Cause-of-death miscoding (e.g., to suicide or other pathways) may shift YLL attribution (46). Cross-national differences in services, legal protections, socioeconomic composition, and reporting lags cannot be fully adjusted (47). Finally, beyond 95% uncertainty intervals, we did not pre-specify scenario-based sensitivity analyses to bound regional under-/over-reporting; these will be prioritised in future work.

## Conclusions

As of 2021, childhood sexual abuse and bullying victimisation impose increasing health burdens on adolescents and young adults globally. Despite reductions in mortality, substantial rises in disability highlight the need for intensified, age-specific, gender-sensitive, and contextually tailored interventions. Addressing structural and behavioural drivers of CSA/B through integrated, multisectoral approaches is essential to narrow global disparities and reduce the escalating burden among the world’s youth.

## Data Availability

Data used in this study were obtained from the Global Health Data Exchange Global Burden of Disease Results Tool (https://vizhub.healthdata.org/gbd-results/), accessed on 10 June 2025.

## References

[1] Cagney J Spencer CN Flor L Herbert M Khalil M O’Connell E. Prevalence of sexual violence against children and age at first exposure: a global analysis by location, age, and sex (1990–2023). Lancet. (2025) 405:1817–36. doi: 10.1016/S0140-6736(25)00311-3, PMID: 40347967 PMC12100463

[2] Kieling C Buchweitz C Caye A Silvani J Ameis SH Brunoni AR. Worldwide prevalence and disability from mental disorders across childhood and adolescence: evidence from the global burden of disease study. JAMA Psychiatry. (2024) 81:347–56. doi: 10.1001/jamapsychiatry.2023.5051, PMID: 38294785 PMC10831630

[3] GRFC. Global burden and strength of evidence for 88 risk factors in 204 countries and 811 subnational locations, 1990–2021: a systematic analysis for the Global Burden of Disease Study 2021. Lancet. (2024) 403:2162–203. doi: 10.1016/s0140-6736(24)00933-4, PMID: 38762324 PMC11120204

[4] Supke M Hahlweg K Schulz W Job A-K. Sex-specific differences in the experience of adverse childhood experiences: transmission, protective, and risk factors from the perspectives of parents and their children–results of an 18-year German longitudinal study. Child Adolesc Psychiatry Ment Health. (2025) 19:46. doi: 10.1186/s13034-025-00904-6, PMID: 40287696 PMC12034139

[5] Venticinque JS McMillan SJ Guyer AE. Expanding understanding of adolescent neural sensitivity to peers: using social information processing theory to generate new lines of research. Dev Cogn Neurosci. (2024) 67:101395–5. doi: 10.1016/j.dcn.2024.101395, PMID: 38823235 PMC11176966

[6] Irani F Muotka J Lyyra P Parviainen T Monto S. Social influence in adolescence: Behavioral and neural responses to peer and expert opinion. Soc Neurosci. (2024) 19:1–12. doi: 10.1080/17470919.2024.2323745, PMID: 38426851

[7] Wang W Wang Y Shao K Lei Z Cheng L Wang F. Global, regional, and national burden of bullying related mental disorders of adolescent from 1990 to 2019: a systematic analysis for the Global Burden of Disease Study 2019. Psychiatry Res. (2024) 341:116154–4. doi: 10.1016/j.psychres.2024.116154, PMID: 39217828

[8] BD 2021 Diseases and Injuries Collaborators. Global incidence, prevalence, years lived with disability (YLDs), disability-adjusted life-years (DALYs), and healthy life expectancy (HALE) for 371 diseases and injuries in 204 countries and territories and 811 subnational locations, 1990–2021: a systematic analysis for the Global Burden of Disease Study 2021. Lancet. (2024) 403:2133–61. doi: 10.1016/S0140-6736(24)00757-8, PMID: 38642570 PMC11122111

[9] GRFC. Global burden of 87 risk factors in 204 countries and territories, 1990–2019: a systematic analysis for the global burden of disease study 2019. Lancet. (2020) 396:1223–49. doi: 10.1016/s0140-6736(20)30752-2, PMID: 33069327 PMC7566194

[10] Roberts C Freeman J Samdal O Schnohr CW de Looze ME Nic Gabhainn S. The Health Behaviour in School-aged Children (HBSC) study: methodological developments and current tensions. Int J Public Health. (2009) 54:140–50. doi: 10.1007/s00038-009-5405-9, PMID: 19639259 PMC2732766

[11] Zheng P Barber R Sorensen R Murray C Aleksandr A. Trimmed constrained mixed effects models: formulations and algorithms. bioRxiv (Cold Spring Harbor Laboratory). (2020) 30:544–56. doi: 10.1101/2020.01.28.923599

[12] Spencer CN Khalil M Herbert M Aravkin AY Arrieta A Baeza MJ. Health effects associated with exposure to intimate partner violence against women and childhood sexual abuse: a Burden of Proof study. Nat Med. (2023) 29:3243–58. doi: 10.1038/s41591-023-02629-5, PMID: 38081957 PMC10719101

[13] Lu M Li D Hu Y Zhang L Li Y Zhang Z. Persistence of severe global inequalities in the burden of Hypertension Heart Disease from 1990 to 2019: findings from the global burden of disease study 2019. BMC Public Health. (2024) 24:110. doi: 10.1186/s12889-023-17573-9, PMID: 38184560 PMC10771693

[14] Li X Xiao X Wu Z Li A Wang W Lin R. Global, regional, and national burden of early-onset colorectal cancer and projection to 2050: An analysis based on the Global Burden of Disease Study 2021. Public Health. (2025) 238:245–53. doi: 10.1016/j.puhe.2024.12.011, PMID: 39700867

[15] He C Lu S Yu H Sun Y Zhang X. Global, regional, and national disease burden attributable to high systolic blood pressure in youth and young adults: 2021 Global Burden of Disease Study analysis. BMC Med. (2025) 23:74. doi: 10.1186/s12916-025-03918-1, PMID: 39915840 PMC11804021

[16] Mokdad AH Bisignano C Hsu JM Aldridge RW Aravkin AY Brauer M. The burden of diseases, injuries, and risk factors by state in the USA, 1990–2021: a systematic analysis for the Global Burden of Disease Study 2021. Lancet. (2024) 404:2314–40. doi: 10.1016/s0140-6736(24)01446-6, PMID: 39645376 PMC11694014

[17] Biswas T Scott JG Munir K Thomas HJ Huda MM Hasan MM. Global variation in the prevalence of bullying victimisation amongst adolescents: Role of peer and parental supports. EClinicalMedicine. (2020) 20:100276. doi: 10.1016/j.eclinm.2020.100276, PMID: 32300737 PMC7152826

[18] Piolanti A Schmid IE Fiderer FJ Ward CL Stöckl H Foran HM. Global prevalence of sexual violence against children. JAMA Pediatrics. (2025) 179:264–72. doi: 10.1001/jamapediatrics.2024.5326, PMID: 39804632 PMC11877167

[19] Molcho M Walsh SD King N Pickett W Donnelly PD Cosma A. Trends in indicators of violence among adolescents in Europe and North America 1994–2022. Int J Public Health. (2025) 70:1607654. doi: 10.3389/ijph.2025.1607654, PMID: 40065986 PMC11891013

[20] Gaffney H Ttofi MM Farrington DP. Effectiveness of school-based programs to reduce bullying perpetration and victimization: An updated systematic review and meta-analysis. Campbell Systematic Rev. (2021) 17:1–102. doi: 10.1002/cl2.1143, PMID: 37131921 PMC8356322

[21] Chiang L Miedema S Saul J Mercy J Brooks A Butchart A. Successful child sexual violence prevention efforts start with data: how the Violence Against Children and Youth Survey helped curb the tide of child sexual violence in 20 countries. BMJ paediatrics Open. (2024) 8:e002497–e002497. doi: 10.1136/bmjpo-2024-002497, PMID: 38479727 PMC10936510

[22] Hong C Liu Z Gao L Jin Y Shi J Liang R. Global trends and regional differences in the burden of anxiety disorders and major depressive disorder attributed to bullying victimisation in 204 countries and territories, 1999–2019: an analysis of the Global Burden of Disease Study. Epidemiol Psychiatr Sci. (2022) 31:e85. doi: 10.1017/s2045796022000683, PMID: 36440549 PMC9714217

[23] Graves JM Abshire DA Koontz E Mackelprang JL. Identifying challenges and solutions for improving access to mental health services for rural youth: insights from adult community members. Int J Environ Res Public Health. (2024) 21:725. doi: 10.3390/ijerph21060725, PMID: 38928971 PMC11203972

[24] Sobhani M Parisa S Mahdi N Reza K Behnaz S Shima I. Improving mental health infrastructure across the Middle East. Asian J Psychiatry. (2024) 93:103908. doi: 10.1016/j.ajp.2023.103908, PMID: 38237532

[25] Yu R Perera C Sharma M Ipince A Bakrania S Shokraneh F. Child and adolescent mental health and psychosocial support interventions: An evidence and gap map of low- and middle-income countries. Campbell Systematic Rev. (2023) 19:e1349. doi: 10.1002/cl2.1349, PMID: 37621301 PMC10445093

[26] Huang H Ding Y Wan X Liang Y Zhang Y Lu G. A meta-analysis of the relationship between bullying and non-suicidal self-injury among children and adolescents. Sci Rep. (2022) 12:17285. doi: 10.1038/s41598-022-22122-2, PMID: 36241694 PMC9568539

[27] Vrijen C Wiertsema M Ackermans MA van der Ploeg R Kretschmer T. Childhood and adolescent bullying perpetration and later substance use: A meta-analysis. Pediatrics. (2021) 147:e2020034751. doi: 10.1542/peds.2020-034751, PMID: 33597287

[28] Konovalov I. Contribution of China and India to the development of cooperation of Asia-Pacific countries in science and technology. Inf Innovations. (2021) 16:66–73. doi: 10.31432/1994-2443-2021-16-3-66-73

[29] Swaminathan S Hemalatha R Pandey A Kassebaum NJ Laxmaiah A Longvah T. The burden of child and maternal malnutrition and trends in its indicators in the states of India: the Global Burden of Disease Study 1990–2017. Lancet Child Adolesc Health. (2019) 3:855–70. doi: 10.1016/s2352-4642(19)30273-1, PMID: 31542357 PMC6839043

[30] Cerna-Turoff I Fang Z Meierkord A Wu Z Yanguela J Bangirana CA. Factors associated with violence against children in low- and middle-income countries: A systematic review and meta-regression of nationally representative data. Trauma Violence Abuse. (2021) 22:152483802098553. doi: 10.1177/1524838020985532, PMID: 33461441 PMC7961628

[31] Deva MP D’Souza R Sundram S. Developing mental health resources for low and medium income countries of the Pacific—The Cook Islands experience. Asian J Psychiatry. (2010) 3:47–8. doi: 10.1016/j.ajp.2010.01.007, PMID: 23051142

[32] Sharma B Lee TH Nam EW. Loneliness, insomnia and suicidal behavior among school-going adolescents in western Pacific island countries: role of violence and injury. Int J Environ Res Public Health. (2017) 14:791. doi: 10.3390/ijerph14070791, PMID: 28714893 PMC5551229

[33] Astrid Durdei M Valla L Mirjam L Henriksen L. Management of suspicions of child maltreatment at child and family clinics: A mixed-methods study. Int J Child Maltreatment Res Policy Practice. (2024) 7:569–92. doi: 10.1007/s42448-024-00214-y

[34] Ellonen N Lucas S Tindberg Y Janson S. Parents’ Self-reported use of corporal punishment and other humiliating upbringing practices in Finland and Sweden - A comparative study. Child Abuse Review. (2017) 26:289–304. doi: 10.1002/car.2482

[35] Cluver L Orkin M Boyes ME Sherr L. Child and adolescent suicide attempts, suicidal behavior, and adverse childhood experiences in South Africa: A prospective study. J Adolesc Health. (2015) 57:52–9. doi: 10.1016/j.jadohealth.2015.03.001, PMID: 25936843

[36] Winstone L Jamal S Mars B. Cyberbullying perpetration and victimization as risk factors for self-harm: results from a longitudinal cohort study of 13–14-year-olds in England. J Adolesc Health. (2024) 75:298–304. doi: 10.1016/j.jadohealth.2024.04.004, PMID: 38864792

[37] Liu L Wang X Chen B Chui W-H Wang X. Association between child abuse, depression, and school bullying among Chinese secondary school students. Int J Environ Res Public Health. (2022) 20:697. doi: 10.3390/ijerph20010697, PMID: 36613015 PMC9819395

[38] Vallejo Valdivieso PA Zambrano Pincay GH Beltran-Aroca CM Girela-Lopez E. Relationship between child abuse and delinquent behavior in male adolescents deprived of liberty. Int J Environ Res Public Health. (2022) 19:16666. doi: 10.3390/ijerph192416666, PMID: 36554547 PMC9779293

[39] Fraguas D Díaz-Caneja CM Ayora M Durán-Cutilla M Abregú-Crespo R Ezquiaga-Bravo I. Assessment of school anti-bullying interventions. JAMA Pediatrics. (2021) 175:44. doi: 10.1001/jamapediatrics.2020.3541, PMID: 33136156 PMC7607493

[40] Alsaleem S Alsaleem M Asiri A Alkhidhran S Alqahtani WS Alzahrani M. Knowledge and attitude regarding child abuse among primary health care physician in Abha, Saudi Arabia, 2018. J Family Med Primary Care. (2019) 8:706. doi: 10.4103/jfmpc.jfmpc_442_18, PMID: 30984699 PMC6436322

[41] Heymann J Levy JK Bose B Ríos-Salas V Mekonen Y Swaminathan H. Improving health with programmatic, legal, and policy approaches to reduce gender inequality and change restrictive gender norms. Lancet. (2019) 393:2522–34. doi: 10.1016/s0140-6736(19)30656-7, PMID: 31155271

[42] Schindler S Schuster C Olsson MIT Froehlich L Hübner AK Block K. Policy as normative influence? On the relationship between parental leave policy and social norms in gender division of childcare across 48 countries. Br J Soc Psychol. (2024) 64:e12806. doi: 10.1111/bjso.12806, PMID: 39439425

[43] Do HP Dunne MP Vo TV Nguyen LH Luong-Thanh B-Y Valdebenito S. Applying the WHO INSPIRE framework to ending violence against pregnant women and unborn children: A case study in Vietnam. Violence Against Women. (2024) 31:813–40. doi: 10.1177/10778012241230324, PMID: 38380997

[44] Thomas JC Kopel J. Male victims of sexual assault: a review of the literature. Behav Sci. (2023) 13:304. doi: 10.3390/bs13040304, PMID: 37102818 PMC10135558

[45] Greenland S. Ecologic versus individual-level sources of bias in ecologic estimates of contextual health effects. Int J Epidemiol. (2001) 30:1343–50. doi: 10.1093/ije/30.6.1343, PMID: 11821344

[46] Kapusta ND. Declining autopsy rates and suicide misclassification. Arch Gen Psychiatry. (2011) 68:1050. doi: 10.1001/archgenpsychiatry.2011.66, PMID: 21646567

[47] Dosunmu EF Emeh RO Dixit S Bakeer MK Coats MT Owen DR. The anti-microbial peptide TP359 attenuates inflammation in human lung cells infected with Pseudomonas aeruginosa via TLR5 and MAPK pathways. PLoS One. (2017) 12:e0176640–e0176640. doi: 10.1371/journal.pone.0176640, PMID: 28467446 PMC5415104

