# Supplementary material for: Global, regional and national burden of childhood sexual abuse and bullying in adolescents and young adults: a Global Burden of Disease 2021 analysis
Source: Front Psychiatry. 2025 Oct 30;16:1679479. doi: 10.3389/fpsyt.2025.1679479 (PMC12612861; doi:10.3389/fpsyt.2025.1679479)
Supplement: Supplementary file 1 [file DataSheet1.docx]

**Table S1.** Changes in childhood sexual abuse and bullying-related deaths and death rates in adolescents and young adults by sex and SDI region, 1990-2021

| **Location** | **Death cases (95% UI)** | | **Percentage change in death case (95%UI)** | **Death rate/100, 000(95%UI)** | | **Percentage change in death rate/100, 000 (95%UI)** | **EAPC of death rate**  **/100, 000 (95%CI)** |
| --- | --- | --- | --- | --- | --- | --- | --- |
|  | **1990** | **2021** | **1990-2021** | **1990** | **2021** | **1990-2021** | **1990-2021** |
| **Global** | | | | | | | |
| Global both | 260.31 (45.95, 615.84) | 186.67 (33.69, 453.54) | -28.29(-52.02,12.11) | 0.02 (0.00, 0.04) | 0.01 (0.00, 0.02) | -41.23(-60.68, -8.12) | -2.26(-2.60, -1.92) |
| Global woman | 44.87 (6.99, 101.32) | 30.74 (4.47, 66.70) | -31.50(-42.35, -15.42) | 0.01 (0.00, 0.01) | 0.00 (0.00, 0.01) | -43.35(-52.33, -30.05) | -2.80(-3.35, -2.24) |
| Global man | 215.44 (38.70, 508.89) | 155.94 (27.26, 391.69) | -27.62(-55.82,21.44) | 0.03 (0.00, 0.06) | 0.02 (0.00, 0.04) | -41.19(-64.10, -1.32) | -2.17(-2.46, -1.87) |
| **SDI region** | | | | | | | |
| High SDI both | 24.58 (4.01, 57.13) | 26.88 (4.15, 62.15) | 9.35(-17.62,47.59) | 0.01 (0.00, 0.03) | 0.01 (0.00, 0.03) | 15.41(-13.06,55.76) | 0.85(0.64,1.07) |
| High SDI woman | 7.95 (1.21, 17.31) | 12.13 (1.73, 26.67) | 52.68(20.30,87.44) | 0.01 (0.00, 0.02) | 0.01 (0.00, 0.03) | 62.18(27.79,99.11) | 2.23(2.00,2.46) |
| High SDI man | 16.64 (2.78, 41.53) | 14.75 (2.50, 37.86) | -11.35(-44.65,44.57) | 0.02 (0.00, 0.04) | 0.02 (0.00, 0.04) | -7.01(-41.94,51.65) | 0.03(-0.27,0.32) |
| High-middle SDI both | 63.87 (10.16, 151.26) | 16.42 (2.71, 38.92) | -74.30(-83.56, -61.14) | 0.02 (0.00, 0.05) | 0.01 (0.00, 0.02) | -67.71(-79.35, -51.18) | -5.23(-6.42, -4.02) |
| High-middle SDI woman | 16.14 (2.65, 36.43) | 3.80 (0.46, 8.98) | -76.48(-82.15, -69.75) | 0.01 (0.00, 0.03) | 0.00 (0.00, 0.01) | -69.51(-76.87, -60.79) | -5.60(-7.14, -4.04) |
| High-middle SDI man | 47.73 (7.47, 115.40) | 12.62 (2.16, 31.77) | -73.56(-85.37, -55.78) | 0.03 (0.01, 0.08) | 0.01 (0.00, 0.03) | -67.73(-82.15, -46.04) | -5.17(-6.24, -4.08) |
| Middle SDI both | 97.74 (16.52, 216.88) | 45.36 (7.64, 111.72) | -53.59(-73.53, -22.73) | 0.02 (0.00, 0.04) | 0.01 (0.00, 0.02) | -53.92(-73.72, -23.27) | -2.43(-2.59, -2.28) |
| Middle SDI woman | 14.78 (2.17, 33.90) | 4.06 (0.64, 9.61) | -72.50(-80.27, -56.85) | 0.01 (0.00, 0.01) | 0.00 (0.00, 0.00) | -72.21(-80.06, -56.39) | -4.30(-4.42, -4.18) |
| Middle SDI man | 82.96 (14.34, 187.47) | 41.29 (6.56, 101.39) | -50.22(-73.54, -11.82) | 0.03 (0.01, 0.07) | 0.01 (0.00, 0.04) | -51.40(-74.17, -13.91) | -2.22(-2.40, -2.04) |
| Low-middle SDI both | 63.16 (10.45, 157.39) | 75.90 (11.87, 197.63) | 20.17(-40.50,141.81) | 0.02 (0.00, 0.04) | 0.01 (0.00, 0.04) | -21.36(-61.06,58.24) | -0.83(-0.97, -0.68) |
| Low-middle SDI woman | 3.53 (0.54, 8.54) | 4.14 (0.68, 10.07) | 17.15(-9.55,97.11) | 0.00 (0.00, 0.00) | 0.00 (0.00, 0.00) | -22.95(-40.51,29.63) | -1.13(-1.25, -1.01) |
| Low-middle SDI man | 59.63 (8.96, 151.22) | 71.76 (11.14, 184.91) | 20.35(-42.69,149.05) | 0.03 (0.00, 0.08) | 0.03 (0.00, 0.07) | -21.62(-62.68,62.19) | -0.83(-0.97, -0.68) |
| Low SDI both | 10.81 (1.67, 26.25) | 21.99 (3.20, 51.71) | 103.46(13.72,291.13) | 0.01 (0.00, 0.02) | 0.01 (0.00, 0.01) | -14.27(-52.08,64.81) | -0.47(-0.63, -0.30) |
| Low SDI woman | 2.45 (0.32, 5.72) | 6.58 (0.98, 15.10) | 168.72(89.03,411.21) | 0.00 (0.00, 0.01) | 0.00 (0.00, 0.01) | 13.05(-20.48,115.05) | 0.24(0.02,0.46) |
| Low SDI man | 8.36 (1.31, 21.90) | 15.41 (1.86, 38.56) | 84.35(-21.89,316.23) | 0.01 (0.00, 0.03) | 0.01 (0.00, 0.02) | -22.19(-67.03,75.67) | -0.71(-0.90, -0.53) |
| SDI, sociodemographic index; UI, uncertainty interval; CI, confidence interval; EAPC, estimated annual percentage change. | | | | | | | |

**Table S2.** Changes in childhood sexual abuse and bullying-related DALYs and DALY rates in adolescents and young adults by sex and SDI region, 1990-2021

| **Location** | **DALYs (95% UI)** | | **Percentage change in DALYs (95%UI)** | **DALY rate/100, 000 (95%UI)** | | **Percentage change in DALY rate/100, 000 (95%UI)** | **EAPC of DALY rate/100, 000 (95%CI)** |
| --- | --- | --- | --- | --- | --- | --- | --- |
|  | **1990** | **2021** | **1990-2021** | **1990** | **2021** | **1990-2021** | **1990-2021** |
| **Global** | | | | | | | |
| Global Both | 2539478.64(1188028.88,4668588.62) | 3686522.88(1762134.17,6574720.99) | 45.17(37.07,55.15) | 164.13(76.79,301.75) | 195.28(93.34,348.28) | 18.98(12.34,27.16) | 0.58(0.51,0.64) |
| Global woman | 1160458.52(525088.38,2266572.98) | 1757275.39(824999.02,3368008.84) | 51.43(42.40,63.16) | 152.47(68.99,297.80) | 190.94(89.64,365.96) | 25.23(17.76,34.93) | 0.78(0.70,0.86) |
| Global man | 1379020.12(665677.53,2481864.90) | 1929247.49(945200.06,3342227.10) | 39.90(30.08,50.18) | 175.43(84.68,315.72) | 199.42(97.70,345.47) | 13.67(5.69,22.03) | 0.40(0.34,0.46) |
| **SDI region** | | | | | | | |
| High SDI both | 437300.46(211460.77,787490.89) | 560207.65(280455.87,972500.62) | 28.11(20.04,40.15) | 223.27(107.97,402.07) | 301.88(151.13,524.05) | 35.20(26.69,47.91) | 1.10(0.92,1.29) |
| High SDI woman | 222222.15(108201.05,410649.40) | 300146.17(153516.87,532820.55) | 35.07(25.44,48.76) | 233.00(113.45,430.56) | 334.29(170.98,593.43) | 43.47(33.25,58.02) | 1.28(1.07,1.50) |
| High SDI man | 215078.31(105065.03,384731.87) | 260061.48(132656.89,446466.53) | 20.91(11.91,31.88) | 214.05(104.56,382.89) | 271.49(138.49,466.09) | 26.84(17.39,38.34) | 0.90(0.75,1.06) |
| High-middle SDI both | 489543.72(225494.62,911395.83) | 418134.62(194819.22,766898.80) | -14.59(-22.24,-4.49) | 172.51(79.46,321.16) | 185.11(86.25,339.52) | 7.31(-2.31,19.99) | 0.22(0.13,0.31) |
| High-middle SDI woman | 236946.11(105915.36,470361.06) | 201122.71(90897.36,391060.65) | -15.12(-22.87,-3.92) | 170.21(76.08,337.88) | 187.23(84.62,364.05) | 10.00(-0.04,24.52) | 0.36(0.29,0.43) |
| High-middle SDI man | 252597.61(120486.77,460707.28) | 217011.91(105632.18,374731.69) | -14.09(-23.47,-2.98) | 174.72(83.34,318.67) | 183.19(89.17,316.33) | 4.85(-6.60,18.41) | 0.09(-0.02,0.20) |
| Middle SDI both | 825834.07(376810.33,1536430.78) | 922148.57(426155.97,1721678.27) | 11.66(4.68,18.50) | 150.47(68.66,279.95) | 166.83(77.10,311.48) | 10.87(3.94,17.66) | 0.31(0.22,0.39) |
| Middle SDI woman | 366931.00(158702.75,727234.20) | 420579.72(181870.86,833911.48) | 14.62(7.67,22.43) | 135.89(58.77,269.32) | 157.41(68.07,312.12) | 15.84(8.81,23.74) | 0.51(0.42,0.61) |
| Middle SDI man | 458903.07(219959.59,848003.49) | 501568.85(243668.38,887771.91) | 9.30(-0.36,17.15) | 164.60(78.90,304.16) | 175.64(85.33,310.88) | 6.71(-2.73,14.37) | 0.13(0.05,0.22) |
| Low-middle SDI both | 581789.83(272416.18,1059877.63) | 1128937.32(552467.13,1968846.11) | 94.05(78.79,112.94) | 160.84(75.31,293.02) | 204.24(99.95,356.19) | 26.98(17.00,39.35) | 0.79(0.75,0.83) |
| Low-middle SDI woman | 250900.78(112461.01,487754.95) | 537054.71(250422.08,994379.78) | 114.05(95.49,140.59) | 140.60(63.02,273.33) | 197.93(92.29,366.48) | 40.78(28.57,58.23) | 1.14(1.09,1.20) |
| Low-middle SDI man | 330889.04(161717.11,577778.35) | 591882.61(303094.48,994765.94) | 78.88(63.52,96.89) | 180.55(88.24,315.27) | 210.33(107.70,353.49) | 16.49(6.49,28.22) | 0.50(0.47,0.54) |
| Low SDI both | 203058.96(97710.65,370479.48) | 654586.21(307701.77,1186254.11) | 222.36(205.46,239.96) | 130.45(62.77,238.00) | 177.19(83.29,321.12) | 35.83(28.71,43.25) | 1.01(0.99,1.03) |
| Low SDI woman | 82525.93(39824.64,159943.21) | 297201.21(137386.76,574139.95) | 260.13(230.85,287.66) | 106.66(51.47,206.72) | 161.59(74.70,312.17) | 51.50(39.18,63.08) | 1.38(1.36,1.41) |
| Low SDI man | 120533.03(59063.05,215090.62) | 357385.00(172195.69,628898.27) | 196.50(176.22,216.47) | 153.96(75.44,274.74) | 192.66(92.83,339.04) | 25.14(16.58,33.57) | 0.73(0.71,0.75) |
| DALYs, disability-adjusted life years; SDI, sociodemographic index; UI, uncertainty interval; CI, confidence interval; EAPC, estimated annual percentage change. | | | | | | | |

**Table S3.** Ranks of childhood sexual abuse and bullying-related deaths, death rates, and changes in adolescents and young adults in 204 countries/territories, 1990-2021

| **The rank of death cases** | | |  | **The rank of percentage change in death case** | | |  | **The rank of death rates** | | |  | **The rank of percentage change in death rate** | | |
| --- | --- | --- | --- | --- | --- | --- | --- | --- | --- | --- | --- | --- | --- | --- |
| **(Descending)** | | |  | **(Descending) 1990-2021** | | |  | **(Descending)** | | |  | **(Descending) 1990-2021** | | |
| **Country or region** | **Rank** | |  | **Country or region** | **Rank** | **Value (95%UI)** |  | **Country or region** | **Rank** | |  | **Country or region** | **Rank** | **Value (95%UI)** |
|  | **1990** | **2021** |  |  |  |  |  |  | **1990** | **2021** |  |  |  |  |
| People's Republic of China | 1 | 2 |  | Chad | 1 | 462.14 (118.85, 1574.27) |  | Greenland | 1 | 1 |  | Sao Tome and Principe | 1 | 112.5767(-50.4580,710.1719) |
| Republic of India | 2 | 1 |  | Cameroon | 2 | 362.26 (92.58, 1109.60) |  | Republic of Guatemala | 2 | 3 |  | United States Virgin Islands | 2 | 100.6097(-41.6104,484.8313) |
| Russian Federation | 3 | 5 |  | Mali | 3 | 360.49 (87.94, 1101.75) |  | Republic of the Union of Myanmar | 3 | 2 |  | Lesotho | 3 | 91.5073(-19.0409,307.5417) |
| Republic of the Union of Myanmar | 4 | 4 |  | Burkina Faso | 4 | 349.55 (95.63, 1087.90) |  | Republic of El Salvador | 4 | 6 |  | Burkina Faso | 4 | 81.2594(-21.1233,378.9601) |
| United Mexican States | 5 | 9 |  | Benin | 5 | 346.34 (55.90, 1134.97) |  | Mongolia | 5 | 5 |  | Chad | 5 | 75.9259(-31.5111,423.9724) |
| United States of America | 6 | 3 |  | Niger | 6 | 338.57 (66.78, 1150.47) |  | Ukraine | 6 | 23 |  | Sierra Leone | 6 | 74.9460(-36.1723,380.6592) |
| Ukraine | 7 | 18 |  | Gambia | 7 | 337.06 (63.97, 1082.14) |  | Russian Federation | 7 | 11 |  | United States of America | 7 | 72.2296(8.6955,191.8365) |
| Federative Republic of Brazil | 8 | 6 |  | Guinea | 8 | 328.22 (68.23, 1065.81) |  | Republic of Kazakhstan | 8 | 58 |  | Gambia | 8 | 67.9820(-36.9773,354.3484) |
| Federal Republic of Germany | 9 | 12 |  | Sierra Leone | 9 | 318.57 (52.71, 1050.02) |  | Republic of Belarus | 9 | 14 |  | Canada | 9 | 67.9060(2.1135,171.6677) |
| Republic of Guatemala | 10 | 10 |  | Uganda | 10 | 308.96 (79.56, 844.57) |  | Kyrgyz Republic | 10 | 28 |  | Guinea | 10 | 65.6315(-34.9300,350.9264) |
| Republic of Kazakhstan | 11 | 42 |  | Liberia | 11 | 297.51 (62.18, 965.70) |  | New Zealand | 11 | 13 |  | Belize | 11 | 62.1374(-39.1758,369.8301) |
| Republic of Korea | 12 | 36 |  | Sao Tome and Principe | 12 | 270.71 (-13.60, 1312.86) |  | Republic of Estonia | 12 | 33 |  | Liberia | 12 | 60.7045(-34.4351,330.8448) |
| Republic of Uzbekistan | 13 | 55 |  | Togo | 13 | 247.62 (30.99, 853.62) |  | Republic of Latvia | 13 | 34 |  | Zimbabwe | 13 | 59.7025(-32.4982,272.4394) |
| Kingdom of Thailand | 14 | 13 |  | Belize | 14 | 242.61 (28.53, 892.80) |  | Republic of Lithuania | 14 | 22 |  | Togo | 14 | 58.5759(-40.2471,335.0236) |
| Islamic Republic of Pakistan | 15 | 8 |  | Mozambique | 15 | 221.54 (47.16, 604.78) |  | Saint Kitts and Nevis | 15 | 7 |  | Uganda | 15 | 52.2923(-33.1350,251.7501) |
| Democratic Socialist Republic of Sri Lanka | 16 | 39 |  | Somalia | 16 | 208.66 (60.47, 512.34) |  | Turkmenistan | 16 | 16 |  | Benin | 16 | 47.0725(-48.6303,306.9361) |
| Federal Republic of Nigeria | 17 | 7 |  | Guinea-Bissau | 17 | 205.08 (31.30, 806.07) |  | Republic of Nicaragua | 17 | 17 |  | Guinea-Bissau | 17 | 46.7372(-36.8477,335.7947) |
| Republic of Belarus | 18 | 45 |  | Republic of Côte d'Ivoire | 18 | 201.30 (3.18, 804.95) |  | Democratic Socialist Republic of Sri Lanka | 18 | 64 |  | Mali | 18 | 46.9838(-40.0117,283.5894) |
| Republic of El Salvador | 19 | 23 |  | Senegal | 19 | 196.57 (12.07, 750.84) |  | Republic of Honduras | 19 | 12 |  | Bahamas | 19 | 46.3941(-38.5363,217.6681) |
| Republic of the Philippines | 20 | 15 |  | Equatorial Guinea | 20 | 189.85 (-6.09, 691.07) |  | Bosnia and Herzegovina | 20 | 9 |  | Saint Vincent and the Grenadines | 20 | 44.5480(-35.6330,243.4523) |
| Republic of Poland | 21 | 22 |  | Yemen | 21 | 181.78 (23.26, 543.70) |  | Republic of Uzbekistan | 21 | 127 |  | Cameroon | 21 | 43.5083(-40.2136,275.5222) |
| United Kingdom of Great Britain and Northern Ireland | 22 | 14 |  | Angola | 22 | 169.62 (17.31, 520.24) |  | Republic of Haiti | 22 | 8 |  | United Kingdom | 22 | 42.5869(-32.8765,183.0839) |
| Democratic Republic of the Congo | 23 | 11 |  | Nigeria | 23 | 169.43 (18.30, 573.63) |  | United Mexican States | 23 | 51 |  | Senegal | 23 | 37.3164(-48.1112,293.9494) |
| Republic of Peru | 24 | 34 |  | Afghanistan | 24 | 150.10 (21.65, 473.67) |  | Republic of Azerbaijan | 24 | 34 |  | Côte d'Ivoire | 24 | 33.8644(-54.1575,302.0549) |
| Republic of South Africa | 25 | 24 |  | Lesotho | 25 | 145.29 (3.69, 421.99) |  | Federal Republic of Germany | 25 | 29 |  | Paraguay | 25 | 31.7015(-46.4120,188.5421) |
| Kyrgyz Republic | 26 | 58 |  | Madagascar | 26 | 128.86 (14.05, 405.42) |  | Republic of Tajikistan | 26 | 18 |  | Indonesia | 26 | 31.8149(-55.8724,247.6572) |
| Romania | 27 | 71 |  | Djibouti | 27 | 128.12 (-5.03, 424.02) |  | Republic of Mauritius | 27 | 40 |  | Niger | 27 | 27.4241(-51.5420,263.3183) |
| Republic of Indonesia | 28 | 17 |  | Zimbabwe | 28 | 126.91 (-4.09, 429.18) |  | United States Virgin Islands | 28 | 4 |  | Tonga | 28 | 26.6901(-62.7359,310.5296) |
| Canada | 29 | 16 |  | Papua New Guinea | 29 | 126.73 (-39.14, 629.84) |  | Republic of Korea | 29 | 86 |  | Mozambique | 29 | 25.5683(-42.5320,175.2272) |
| Republic of Haiti | 30 | 19 |  | Kenya | 30 | 120.71 (28.14, 295.15) |  | People's Republic of China | 30 | 63 |  | Dominica | 30 | 23.8555(-40.3479,178.0386) |
| Republic of Azerbaijan | 31 | 50 |  | Mauritania | 31 | 120.59 (-20.85, 548.76) |  | Republic of Slovenia | 31 | 39 |  | Cabo Verde | 31 | 23.2293(-55.1045,229.3046) |
| Islamic Republic of Iran | 32 | 38 |  | Pakistan | 32 | 118.86 (-27.38, 542.17) |  | Antigua and Barbuda | 31 | 15 |  | Thailand | 32 | 23.3511(-60.5360,243.2408) |
| Socialist Republic of Viet Nam | 33 | 28 |  | United Republic of Tanzania | 33 | 118.39 (7.79, 366.21) |  | Republic of Costa Rica | 33 | 42 |  | Guyana | 33 | 20.4616(-46.5855,166.9761) |
| Mongolia | 34 | 43 |  | Democratic Republic of the Congo | 34 | 116.82 (-2.45, 406.07) |  | Republic of India | 34 | 24 |  | Monaco | 34 | 18.7212(-56.4914,245.2182) |
| Japan | 35 | 40 |  | Malawi | 35 | 115.47 (-0.12, 359.86) |  | Cook Islands | 35 | 43 |  | Trinidad and Tobago | 35 | 17.7264(-48.5113,183.0721) |
| Republic of Honduras | 36 | 21 |  | Paraguay | 36 | 112.50 (-13.54, 365.55) |  | Solomon Islands | 36 | 21 |  | Antigua and Barbuda | 36 | 16.3625(-45.1022,145.6510) |
| Federal Democratic Republic of Ethiopia | 37 | 26 |  | Zambia | 37 | 111.85 (-13.35, 413.83) |  | Northern Mariana Islands | 37 | 37 |  | South Sudan | 37 | 15.0401(-47.2078,169.4663) |
| New Zealand | 38 | 60 |  | United States of America | 38 | 101.72 (27.30, 241.80) |  | United States of America | 38 | 9 |  | Saint Kitts and Nevis | 38 | 14.9300(-40.4601,132.6133) |
| Republic of Nicaragua | 39 | 41 |  | Vanuatu | 39 | 100.70 (-41.64, 628.26) |  | Republic of Kiribati | 39 | 30 |  | Tokelau | 39 | 11.1505(-67.2601,251.6665) |
| Argentine Republic | 40 | 51 |  | South Sudan | 40 | 97.14 (-9.53, 361.78) |  | Plurinational State of Bolivia | 40 | 64 |  | Cambodia | 40 | 10.9815(-53.7095,206.0165) |
| Republic of Tajikistan | 41 | 29 |  | Solomon Islands | 41 | 97.04 (-30.46, 595.29) |  | Saint Lucia | 41 | 25 |  | Democratic People's Republic of Korea | 41 | 10.5372(-66.9837,203.6639) |
| Democratic People's Republic of Korea | 42 | 30 |  | United Arab Emirates | 42 | 96.05 (-18.28, 409.82) |  | Republic of Armenia | 42 | 116 |  | Somalia | 42 | 8.0667(-43.8156,114.3910) |
| Turkmenistan | 43 | 53 |  | Eritrea | 43 | 93.17 (-6.60, 336.96) |  | Georgia | 43 | 90 |  | Yemen | 43 | 7.8901(-52.8043,146.4633) |
| Federal Democratic Republic of Nepal | 44 | 25 |  | Ghana | 44 | 88.12 (-18.60, 343.66) |  | Republic of Poland | 43 | 34 |  | Solomon Islands | 44 | 7.8653(-61.9299,280.6259) |
| Bosnia and Herzegovina | 45 | 77 |  | Canada | 45 | 86.24 (13.27, 201.34) |  | Grand Duchy of Luxembourg | 45 | 85 |  | Libya | 45 | 7.7234(-56.8447,169.2777) |
| Australia | 46 | 54 |  | Congo | 46 | 83.82 (-13.84, 331.57) |  | Republic of Peru | 46 | 100 |  | Pakistan | 46 | 7.3910(-64.3661,215.1108) |
| French Republic | 47 | 61 |  | Timor-Leste | 47 | 83.09 (-38.85, 452.62) |  | Federative Republic of Brazil | 47 | 49 |  | Saint Lucia | 47 | 5.3451(-46.8824,105.6892) |
| Bolivarian Republic of Venezuela | 48 | 56 |  | Central African Republic | 48 | 80.56 (-13.10, 300.02) |  | Republic of the Marshall Islands | 48 | 43 |  | Nepal | 48 | 5.0235(-70.7578,318.2424) |
| Republic of Ecuador | 49 | 82 |  | Bahamas | 49 | 79.62 (-24.59, 289.76) |  | Republic of Nauru | 49 | 38 |  | Viet Nam | 49 | 4.3303(-69.0966,264.0560) |
| Republic of Lithuania | 50 | 106 |  | Burundi | 50 | 79.59 (-15.74, 307.61) |  | Czech Republic | 49 | 56 |  | Eritrea | 50 | 4.4288(-49.5080,136.2201) |
| Republic of Colombia | 51 | 81 |  | Haiti | 51 | 74.17 (-13.88, 232.56) |  | Central African Republic | 51 | 46 |  | Japan | 51 | 3.2449(-43.5334,110.5997) |
| Republic of Angola | 52 | 20 |  | Palestine | 52 | 73.67 (-47.24, 536.35) |  | Tuvalu | 51 | 50 |  | Malta | 52 | 1.7769(-59.5497,159.7451) |
| Czech Republic | 53 | 80 |  | Nepal | 53 | 66.64 (-53.60, 563.63) |  | Hungary | 53 | 46 |  | Kenya | 53 | 1.4404(-41.1076,81.6162) |
| Plurinational State of Bolivia | 54 | 59 |  | Cabo Verde | 54 | 61.92 (-41.01, 332.71) |  | Romania | 54 | 86 |  | Vanuatu | 54 | 1.2759(-70.5493,267.4868) |
| Hungary | 55 | 76 |  | Cambodia | 55 | 60.24 (-33.16, 341.84) |  | Saint Vincent and the Grenadines | 54 | 20 |  | Micronesia (Federated States of) | 55 | -0.6683(-70.3337,209.5884) |
| Republic of Cuba | 56 | 90 |  | Indonesia | 56 | 53.30 (-48.68, 304.34) |  | Republic of Austria | 56 | 73 |  | Jamaica | 56 | -1.6535(-50.4476,93.3484) |
| Republic of Chile | 57 | 94 |  | Sudan | 57 | 49.86 (-33.11, 255.80) |  | Guam | 56 | 153 |  | Suriname | 57 | -1.5168(-59.2377,149.1018) |
| United Republic of Tanzania | 58 | 27 |  | Honduras | 58 | 48.95 (-49.14, 297.22) |  | Kingdom of Thailand | 58 | 27 |  | Bulgaria | 58 | -1.9347(-68.8459,143.7555) |
| Kingdom of Spain | 59 | 108 |  | Namibia | 59 | 47.83 (-39.74, 263.32) |  | Republic of Serbia | 59 | 93 |  | Mauritania | 59 | -2.2282(-64.9163,187.5515) |
| Republic of Ghana | 60 | 31 |  | United Kingdom | 60 | 45.53 (-31.49, 188.93) |  | Kingdom of Norway | 60 | 110 |  | Nigeria | 60 | -2.4266(-57.1583,143.9507) |
| Republic of Serbia | 61 | 102 |  | Monaco | 61 | 43.92 (-47.26, 318.50) |  | Republic of Ecuador | 61 | 146 |  | Papua New Guinea | 61 | -2.7821(-73.9046,212.9405) |
| Republic of Italy | 62 | 109 |  | Jordan | 62 | 40.87 (-54.64, 274.76) |  | Commonwealth of Dominica | 62 | 30 |  | Finland | 62 | -3.1656(-56.5899,116.3518) |
| Republic of Latvia | 63 | 122 |  | Kiribati | 63 | 40.75 (-44.30, 250.10) |  | Federated States of Micronesia | 63 | 45 |  | Comoros | 63 | -3.2572(-54.7312,225.0399) |
| Republic of Turkey | 64 | 73 |  | Gabon | 64 | 38.90 (-44.08, 227.63) |  | Republic of Equatorial Guinea | 64 | 67 |  | United Republic of Tanzania | 64 | -3.4736(-52.3587,106.0612) |
| Taiwan (Province of China) | 65 | 88 |  | Israel | 65 | 38.40 (-40.32, 221.55) |  | Republic of Finland | 64 | 48 |  | Malawi | 65 | -4.2922(-55.6333,104.2612) |
| Republic of Austria | 66 | 97 |  | Comoros | 66 | 38.33 (-35.27, 364.77) |  | Republic of the Congo | 64 | 52 |  | Djibouti | 66 | -5.7418(-60.7573,116.5224) |
| Arab Republic of Egypt | 67 | 136 |  | Lao People's Democratic Republic | 67 | 35.36 (-55.97, 386.08) |  | Canada | 67 | 19 |  | Nauru | 67 | -5.6814(-71.3014,198.7754) |
| Georgia | 68 | 124 |  | Iraq | 68 | 33.65 (-38.46, 203.97) |  | Kingdom of Denmark | 67 | 61 |  | Dominican Republic | 68 | -5.8438(-64.9942,155.2778) |
| Kingdom of Morocco | 69 | 87 |  | Antigua and Barbuda | 69 | 31.85 (-37.80, 178.35) |  | Grenada | 69 | 90 |  | Namibia | 69 | -5.9092(-61.6458,131.2480) |
| Republic of Costa Rica | 70 | 83 |  | India | 70 | 31.47 (-57.18, 276.08) |  | Commonwealth of the Bahamas | 70 | 26 |  | Myanmar | 70 | -6.8569(-72.9696,211.6142) |
| People's Democratic Republic of Algeria | 71 | 85 |  | Libya | 71 | 27.65 (-48.86, 219.08) |  | Tokelau | 70 | 41 |  | Niue | 71 | -6.6198(-73.7121,229.4234) |
| Republic of Estonia | 72 | 130 |  | Malaysia | 72 | 26.30 (-64.69, 296.73) |  | Republic of Niue | 72 | 53 |  | Poland | 72 | -7.1115(-64.6555,160.3122) |
| Republic of Sudan | 73 | 57 |  | Qatar | 73 | 22.67 (-65.32, 306.01) |  | Republic of Angola | 73 | 60 |  | Netherlands | 73 | -7.7262(-45.1934,60.6826) |
| Islamic Republic of Afghanistan | 74 | 37 |  | Ethiopia | 74 | 18.16 (-36.05, 196.46) |  | Republic of Croatia | 74 | 77 |  | Greece | 74 | -7.8166(-63.4770,139.3876) |
| Kingdom of Belgium | 75 | 92 |  | Tonga | 75 | 17.85 (-65.34, 281.87) |  | Gabonese Republic | 75 | 66 |  | Madagascar | 75 | -8.7863(-54.5448,101.4429) |
| Malaysia | 76 | 67 |  | Suriname | 76 | 14.56 (-52.58, 189.77) |  | American Samoa | 76 | 69 |  | Haiti | 76 | -9.6199(-55.3097,72.5707) |
| Syrian Arab Republic | 77 | 104 |  | Tuvalu | 77 | 14.18 (-63.78, 280.69) |  | Australia | 76 | 81 |  | Congo | 77 | -10.1703(-57.8939,110.8984) |
| Republic of Kenya | 78 | 46 |  | Dominican Republic | 78 | 12.95 (-58.01, 206.22) |  | United Kingdom of Great Britain and Northern Ireland | 78 | 32 |  | Taiwan (Province of China) | 78 | -10.1995(-64.5843,122.9457) |
| Kingdom of Cambodia | 79 | 62 |  | Saint Kitts and Nevis | 79 | 10.23 (-42.89, 123.10) |  | Democratic Republic of the Congo | 79 | 61 |  | Timor-Leste | 79 | -10.9557(-70.2588,168.7652) |
| Republic of Madagascar | 80 | 48 |  | Tajikistan | 80 | 10.13 (-52.83, 136.00) |  | Swiss Confederation | 80 | 121 |  | Zambia | 80 | -10.9891(-63.5936,115.8891) |
| Republic of Armenia | 81 | 139 |  | Eswatini | 81 | 10.13 (-50.74, 102.85) |  | Republic of Cuba | 81 | 84 |  | Kiribati | 81 | -11.0195(-64.7881,121.3251) |
| Republic of Rwanda | 82 | 89 |  | Democratic People's Republic of Korea | 82 | 9.54 (-67.28, 200.94) |  | Republic of Vanuatu | 82 | 55 |  | Democratic Republic of the Congo | 82 | -12.6009(-60.6791,103.9974) |
| Swiss Confederation | 83 | 112 |  | Oman | 83 | 9.25 (-64.50, 220.72) |  | Democratic People's Republic of Korea | 83 | 54 |  | India | 83 | -14.8185(-72.2577,143.6757) |
| Kingdom of Sweden | 84 | 98 |  | Philippines | 84 | 9.25 (-61.75, 202.68) |  | Lao People's Democratic Republic | 83 | 72 |  | Bhutan | 84 | -14.8948(-76.8632,201.2479) |
| Kingdom of Denmark | 85 | 103 |  | Myanmar | 85 | 8.67 (-68.46, 263.55) |  | Slovak Republic | 85 | 86 |  | Ghana | 85 | -15.6639(-63.5064,98.8963) |
| Republic of Bulgaria | 86 | 111 |  | Nauru | 86 | 6.75 (-67.52, 238.16) |  | Kingdom of Belgium | 86 | 93 |  | Hungary | 86 | -15.5496(-66.2120,107.6982) |
| Republic of Uganda | 87 | 33 |  | Viet Nam | 87 | 2.86 (-69.53, 258.92) |  | Republic of Iceland | 86 | 114 |  | Central African Republic | 87 | -16.1914(-59.6646,85.6697) |
| Republic of Côte d'Ivoire | 88 | 44 |  | Saint Vincent and the Grenadines | 88 | 2.62 (-54.30, 143.84) |  | Kingdom of Bhutan | 86 | 74 |  | Malaysia | 88 | -16.1586(-76.5624,163.3549) |
| Portuguese Republic | 89 | 128 |  | United States Virgin Islands | 89 | -0.50 (-71.04, 190.07) |  | Puerto Rico | 86 | 125 |  | Lao People's Democratic Republic | 89 | -16.3210(-72.7831,200.4895) |
| Lao People's Democratic Republic | 90 | 79 |  | Guyana | 90 | -2.92 (-56.96, 115.15) |  | Republic of Suriname | 90 | 67 |  | Israel | 90 | -16.3659(-63.9365,94.3147) |
| Central African Republic | 91 | 68 |  | Trinidad and Tobago | 91 | -5.98 (-58.88, 126.06) |  | Republic of South Africa | 91 | 105 |  | Palau | 91 | -16.3960(-80.7769,208.1780) |
| Republic of Finland | 92 | 96 |  | Jamaica | 92 | -8.58 (-53.94, 79.74) |  | Republic of Palau | 92 | 79 |  | Eswatini | 92 | -17.5162(-63.1011,51.9320) |
| Republic of Croatia | 93 | 118 |  | Rwanda | 93 | -8.71 (-60.28, 148.46) |  | Republic of Chile | 92 | 149 |  | Angola | 93 | -18.1783(-64.3986,88.2251) |
| Republic of Cameroon | 94 | 32 |  | Finland | 94 | -8.78 (-59.11, 103.81) |  | Kingdom of Sweden | 92 | 97 |  | Bosnia and Herzegovina | 94 | -18.3900(-73.8629,146.7296) |
| Kingdom of Norway | 95 | 117 |  | Tokelau | 95 | -9.11 (-73.23, 187.56) |  | Republic of Panama | 95 | 153 |  | Marshall Islands | 95 | -18.7613(-73.6678,213.6738) |
| Kingdom of the Netherlands | 96 | 101 |  | Micronesia (Federated States of) | 96 | -9.38 (-72.94, 182.42) |  | Republic of the Philippines | 96 | 103 |  | Tuvalu | 96 | -19.3404(-74.4157,168.9250) |
| Slovak Republic | 97 | 115 |  | Botswana | 97 | -9.44 (-61.09, 91.59) |  | Republic of Guyana | 96 | 58 |  | Afghanistan | 97 | -20.6901(-61.4216,81.9189) |
| Dominican Republic | 98 | 91 |  | Dominica | 98 | -10.60 (-56.94, 100.69) |  | Republic of Moldova | 96 | 121 |  | Uruguay | 98 | -21.3132(-67.7252,90.0931) |
| Burkina Faso | 99 | 35 |  | Marshall Islands | 99 | -11.66 (-71.37, 241.08) |  | Barbados | 99 | 110 |  | Fiji | 99 | -21.9840(-81.3800,147.6099) |
| Republic of Malawi | 100 | 64 |  | Netherlands | 100 | -15.22 (-49.65, 47.63) |  | Federal Democratic Republic of Nepal | 99 | 70 |  | Samoa | 100 | -23.0434(-77.9541,173.9989) |
| Republic of Slovenia | 101 | 123 |  | Bangladesh | 101 | -15.29 (-59.60, 123.01) |  | Federal Republic of Nigeria | 101 | 75 |  | Northern Mariana Islands | 101 | -23.8261(-76.9804,163.0959) |
| Republic of the Congo | 102 | 72 |  | Saint Lucia | 102 | -15.69 (-57.49, 64.61) |  | Republic of Bulgaria | 102 | 77 |  | American Samoa | 102 | -24.3750(-77.5073,138.7488) |
| Republic of Zambia | 103 | 69 |  | Maldives | 103 | -16.69 (-70.35, 151.43) |  | Democratic Republic of Timor-Leste | 103 | 86 |  | Gabon | 103 | -24.4046(-69.5676,78.3148) |
| Republic of Mauritius | 104 | 127 |  | Bolivia (Plurinational State of) | 104 | -16.80 (-73.00, 113.26) |  | Republic of Botswana | 104 | 121 |  | San Marino | 104 | -24.6231(-72.5007,129.5585) |
| Puerto Rico | 105 | 137 |  | Samoa | 105 | -18.36 (-76.61, 190.68) |  | Bolivarian Republic of Venezuela | 104 | 110 |  | Brazil | 105 | -24.7962(-64.3290,54.4882) |
| Republic of Moldova | 106 | 140 |  | Thailand | 106 | -19.09 (-74.11, 125.14) |  | Islamic Republic of Pakistan | 106 | 80 |  | Denmark | 106 | -24.8558(-63.0442,62.6533) |
| Republic of Senegal | 107 | 63 |  | Malta | 107 | -20.90 (-68.56, 101.86) |  | Republic of Rwanda | 106 | 142 |  | North Macedonia | 107 | -24.5307(-77.2464,119.8535) |
| Independent State of Papua New Guinea | 108 | 75 |  | Fiji | 108 | -21.48 (-81.26, 149.21) |  | Republic of Ghana | 108 | 105 |  | United Arab Emirates | 108 | -26.0269(-69.1670,92.3603) |
| Republic of the Niger | 109 | 49 |  | Brazil | 109 | -21.63 (-62.83, 60.98) |  | Argentine Republic | 109 | 142 |  | Montenegro | 109 | -27.2517(-72.9335,105.4744) |
| Republic of Mali | 109 | 47 |  | South Africa | 110 | -22.76 (-64.98, 54.80) |  | Republic of Trinidad and Tobago | 110 | 75 |  | Singapore | 110 | -27.1238(-57.7113,25.3762) |
| Republic of Burundi | 111 | 86 |  | Bhutan | 111 | -23.44 (-79.19, 171.02) |  | Independent State of Papua New Guinea | 110 | 96 |  | Iran (Islamic Republic of) | 111 | -27.8641(-62.4900,33.4098) |
| Federal Republic of Somalia | 112 | 70 |  | Guatemala | 112 | -23.64 (-71.91, 86.64) |  | Belize | 112 | 56 |  | Tunisia | 112 | -28.2824(-70.5182,91.7201) |
| State of Israel | 113 | 105 |  | Cyprus | 113 | -24.58 (-77.02, 113.12) |  | Bermuda | 113 | 130 |  | Honduras | 113 | -28.2968(-75.5164,91.2152) |
| Republic of Panama | 114 | 132 |  | Saudi Arabia | 114 | -25.51 (-63.99, 57.94) |  | Independent State of Samoa | 114 | 121 |  | Palestine | 114 | -28.6444(-78.3222,161.4611) |
| Republic of Chad | 115 | 52 |  | Sweden | 115 | -25.93 (-64.27, 33.77) |  | Islamic Republic of Afghanistan | 114 | 116 |  | Venezuela (Bolivarian Republic of) | 115 | -29.3223(-73.1954,76.3263) |
| Republic of Mozambique | 116 | 74 |  | Uruguay | 116 | -26.14 (-69.70, 78.44) |  | Republic of Guinea-Bissau | 116 | 70 |  | Belgium | 116 | -29.7374(-65.2648,50.0150) |
| Republic of Guinea | 117 | 65 |  | Nicaragua | 117 | -26.46 (-73.32, 111.29) |  | Kingdom of Cambodia | 117 | 100 |  | Ireland | 117 | -30.0490(-67.6390,41.9916) |
| Republic of South Sudan | 118 | 100 |  | San Marino | 118 | -26.84 (-73.31, 122.81) |  | Kingdom of Tonga | 118 | 90 |  | Burundi | 118 | -30.2253(-67.2630,58.3636) |
| Republic of Benin | 119 | 66 |  | Australia | 119 | -27.46 (-57.09, 13.86) |  | Portuguese Republic | 118 | 164 |  | Equatorial Guinea | 119 | -30.9901(-77.6401,88.3463) |
| Republic of Paraguay | 120 | 99 |  | Denmark | 120 | -27.83 (-64.50, 56.23) |  | Dominican Republic | 120 | 116 |  | Sweden | 120 | -30.8921(-66.6658,24.8134) |
| Republic of Tunisia | 121 | 129 |  | Algeria | 121 | -28.10 (-67.20, 65.81) |  | State of Israel | 121 | 127 |  | Philippines | 121 | -31.1560(-75.8980,90.7400) |
| Ireland | 122 | 131 |  | Andorra | 122 | -29.15 (-70.13, 79.44) |  | Republic of Burundi | 122 | 138 |  | Slovakia | 122 | -31.6493(-76.2865,86.3245) |
| Republic of Yemen | 123 | 93 |  | Turkey | 123 | -29.19 (-75.35, 108.25) |  | Montenegro | 122 | 134 |  | Turkey | 123 | -32.4899(-76.4977,98.5584) |
| Togolese Republic | 124 | 83 |  | Ireland | 124 | -29.38 (-67.33, 43.34) |  | Ireland | 124 | 142 |  | Sudan | 124 | -32.5658(-69.9014,60.1066) |
| Republic of Sierra Leone | 125 | 78 |  | American Samoa | 125 | -29.96 (-79.17, 121.12) |  | Brunei Darussalam | 125 | 167 |  | Cyprus | 125 | -33.7593(-79.8190,87.1740) |
| Greenland | 126 | 147 |  | Venezuela (Bolivarian Republic of) | 126 | -30.09 (-73.49, 74.42) |  | Taiwan (Province of China) | 126 | 129 |  | Czechia | 126 | -33.5099(-70.9453,50.8398) |
| State of Eritrea | 127 | 110 |  | Costa Rica | 127 | -30.33 (-69.61, 59.37) |  | Syrian Arab Republic | 127 | 167 |  | Andorra | 127 | -33.9257(-72.1452,67.3340) |
| People's Republic of Bangladesh | 128 | 133 |  | Iran (Islamic Republic of) | 128 | -30.66 (-63.94, 28.24) |  | Republic of the Gambia | 127 | 83 |  | Tajikistan | 128 | -34.3190(-71.8667,40.7480) |
| Gabonese Republic | 129 | 116 |  | Northern Mariana Islands | 129 | -31.12 (-79.18, 137.90) |  | Republic of Colombia | 129 | 178 |  | Iraq | 129 | -34.5917(-69.8819,48.7584) |
| Republic of Zimbabwe | 130 | 107 |  | Belgium | 130 | -31.44 (-66.10, 46.39) |  | Republic of Paraguay | 129 | 109 |  | Barbados | 130 | -34.6824(-74.4205,56.5972) |
| Republic of Botswana | 131 | 135 |  | Lebanon | 131 | -32.24 (-70.88, 68.05) |  | Republic of Liberia | 129 | 93 |  | France | 131 | -35.2243(-76.5795,42.2947) |
| Republic of Albania | 132 | 157 |  | Morocco | 132 | -32.32 (-71.66, 54.15) |  | Republic of Madagascar | 129 | 131 |  | Cook Islands | 132 | -35.4151(-80.8435,108.5500) |
| State of Libya | 133 | 125 |  | Tunisia | 133 | -32.67 (-72.32, 80.00) |  | North Macedonia | 129 | 149 |  | Bangladesh | 133 | -35.4399(-69.2101,69.9548) |
| Republic of Liberia | 134 | 95 |  | Turkmenistan | 134 | -32.82 (-67.94, 37.25) |  | Federal Democratic Republic of Ethiopia | 134 | 170 |  | Cuba | 134 | -35.5869(-73.4890,69.9117) |
| Eastern Republic of Uruguay | 135 | 142 |  | Japan | 135 | -35.42 (-64.68, 31.73) |  | Burkina Faso | 134 | 81 |  | Oman | 135 | -35.9788(-79.1941,87.9452) |
| Kingdom of Saudi Arabia | 136 | 146 |  | Singapore | 136 | -36.01 (-62.87, 10.08) |  | Republic of Cameroon | 136 | 105 |  | South Africa | 136 | -36.3368(-71.1367,27.5951) |
| Solomon Islands | 137 | 120 |  | Brunei Darussalam | 137 | -36.08 (-74.47, 58.33) |  | Kingdom of the Netherlands | 136 | 134 |  | Bermuda | 137 | -37.1325(-76.9874,48.8975) |
| Republic of Trinidad and Tobago | 138 | 143 |  | Poland | 138 | -36.94 (-76.01, 76.71) |  | Republic of Zambia | 136 | 138 |  | Botswana | 138 | -37.5271(-73.1584,32.1729) |
| Republic of Guyana | 139 | 141 |  | Palau | 139 | -37.28 (-85.58, 131.19) |  | Islamic Republic of Iran | 136 | 156 |  | Croatia | 139 | -38.7400(-79.5559,68.7419) |
| Hellenic Republic | 140 | 150 |  | France | 140 | -37.39 (-77.36, 37.55) |  | Eastern Republic of Uruguay | 140 | 153 |  | Australia | 140 | -39.0457(-63.9449, -4.3250) |
| North Macedonia | 141 | 152 |  | Bahrain | 141 | -37.44 (-79.45, 91.92) |  | United Republic of Tanzania | 140 | 132 |  | Algeria | 141 | -39.7837(-72.5332,38.8561) |
| Kingdom of Bhutan | 141 | 148 |  | Argentina | 142 | -38.63 (-72.37, 25.05) |  | Kingdom of Bahrain | 142 | 188 |  | Maldives | 142 | -40.2212(-78.7267,80.4189) |
| Islamic Republic of Mauritania | 143 | 121 |  | Greece | 143 | -39.27 (-75.94, 57.70) |  | Republic of Benin | 142 | 110 |  | Slovenia | 143 | -40.4711(-74.9954,51.1410) |
| Republic of Guinea-Bissau | 144 | 114 |  | Mongolia | 144 | -39.74 (-73.85, 23.46) |  | Republic of Malawi | 142 | 134 |  | Costa Rica | 144 | -41.3178(-74.4046,34.2318) |
| Republic of Equatorial Guinea | 145 | 119 |  | Luxembourg | 145 | -39.84 (-72.84, 30.62) |  | Togolese Republic | 142 | 104 |  | Morocco | 145 | -41.3548(-75.4479,33.5620) |
| Democratic Republic of Timor-Leste | 145 | 134 |  | Niue | 146 | -42.11 (-83.70, 104.22) |  | French Republic | 146 | 162 |  | Turkmenistan | 146 | -42.2758(-72.4564,17.9311) |
| Kingdom of Lesotho | 147 | 126 |  | Iceland | 147 | -42.56 (-72.37, 12.41) |  | Union of the Comoros | 146 | 138 |  | Albania | 147 | -43.2987(-83.5885,56.5505) |
| State of Kuwait | 148 | 176 |  | Taiwan (Province of China) | 148 | -44.75 (-78.21, 37.16) |  | Republic of Senegal | 146 | 115 |  | Mongolia | 148 | -43.7889(-75.6033,15.1755) |
| United Arab Emirates | 149 | 138 |  | Barbados | 149 | -44.76 (-78.37, 32.43) |  | Republic of Côte d'Ivoire | 146 | 116 |  | Austria | 149 | -45.3225(-74.6523,36.3734) |
| Republic of Namibia | 149 | 144 |  | North Macedonia | 150 | -45.41 (-83.54, 59.02) |  | Republic of Sierra Leone | 150 | 97 |  | Republic of Moldova | 150 | -45.8775(-80.1028,43.4441) |
| Republic of the Gambia | 151 | 113 |  | Syrian Arab Republic | 151 | -45.60 (-72.23, 21.99) |  | Malaysia | 150 | 156 |  | El Salvador | 151 | -46.3115(-81.2440,57.8737) |
| Lebanese Republic | 152 | 158 |  | Montenegro | 152 | -46.20 (-79.99, 51.94) |  | State of Eritrea | 150 | 134 |  | Iceland | 152 | -46.7453(-74.3787,4.2188) |
| Grand Duchy of Luxembourg | 153 | 162 |  | Hungary | 153 | -46.79 (-78.71, 30.87) |  | Republic of Chad | 150 | 100 |  | Bolivia (Plurinational State of) | 153 | -48.4628(-83.2751,32.0971) |
| Republic of Suriname | 154 | 150 |  | El Salvador | 154 | -47.17 (-81.55, 55.34) |  | Republic of Seychelles | 150 | 195 |  | Nicaragua | 154 | -48.7248(-81.3971,47.3228) |
| Commonwealth of the Bahamas | 155 | 145 |  | Bulgaria | 155 | -48.35 (-83.59, 28.39) |  | United Arab Emirates | 155 | 160 |  | Syrian Arab Republic | 155 | -49.0383(-73.9839,14.2910) |
| Sultanate of Oman | 156 | 154 |  | New Zealand | 156 | -50.20 (-69.78, -8.96) |  | Republic of the Niger | 155 | 126 |  | Ethiopia | 156 | -49.6050(-72.7254,26.4391) |
| Kingdom of Eswatini | 157 | 155 |  | Panama | 157 | -50.41 (-82.47, 24.25) |  | Kingdom of Eswatini | 155 | 158 |  | Mauritius | 157 | -49.5063(-83.9444,35.6068) |
| Saint Lucia | 158 | 164 |  | Grenada | 158 | -51.22 (-79.10, 9.43) |  | Republic of Guinea | 155 | 108 |  | Grenada | 158 | -49.5684(-78.3929,13.1472) |
| Hashemite Kingdom of Jordan | 159 | 153 |  | Azerbaijan | 159 | -51.29 (-75.97, -1.10) |  | Kingdom of Spain | 155 | 183 |  | Jordan | 159 | -49.5805(-83.7649,34.1312) |
| Jamaica | 159 | 162 |  | Austria | 160 | -52.50 (-77.98, 18.46) |  | Republic of Albania | 155 | 171 |  | Argentina | 160 | -49.9927(-77.4826,1.8952) |
| United States Virgin Islands | 161 | 160 |  | Peru | 161 | -53.70 (-84.12, 28.18) |  | Socialist Republic of Viet Nam | 161 | 142 |  | Lebanon | 161 | -50.3863(-78.6811,23.0431) |
| Montenegro | 162 | 170 |  | Bermuda | 162 | -53.73 (-83.06, 9.58) |  | Republic of Sudan | 161 | 167 |  | Greenland | 162 | -50.6771(-75.1151,3.5869) |
| Republic of Iceland | 163 | 174 |  | Slovakia | 163 | -55.10 (-84.42, 22.39) |  | Kingdom of Lesotho | 161 | 97 |  | Germany | 163 | -51.1966(-71.6577, -14.2753) |
| Saint Kitts and Nevis | 164 | 165 |  | Cook Islands | 164 | -55.29 (-86.74, 44.37) |  | Islamic Republic of Mauritania | 161 | 149 |  | Rwanda | 164 | -52.1146(-79.1625,30.3315) |
| Guam | 164 | 190 |  | Czechia | 165 | -56.29 (-80.90, -0.84) |  | Republic of Mali | 161 | 116 |  | Puerto Rico | 165 | -52.0611(-85.2168,36.6732) |
| Saint Vincent and the Grenadines | 166 | 167 |  | Switzerland | 166 | -57.75 (-77.99, -12.50) |  | State of Kuwait | 166 | 198 |  | Azerbaijan | 166 | -53.3050(-76.9654, -5.1868) |
| Union of the Comoros | 167 | 161 |  | Germany | 167 | -58.64 (-75.98, -27.35) |  | Republic of Namibia | 167 | 158 |  | Serbia | 167 | -53.7650(-86.0996,42.1971) |
| Barbados | 167 | 178 |  | Norway | 168 | -59.01 (-77.64, -20.49) |  | Federal Republic of Somalia | 168 | 149 |  | Brunei Darussalam | 168 | -54.0829(-81.6632,13.7419) |
| Republic of Singapore | 167 | 173 |  | Cuba | 169 | -59.58 (-83.36, 6.63) |  | Republic of Cabo Verde | 168 | 138 |  | Saudi Arabia | 169 | -54.3770(-77.9436, -3.2642) |
| Republic of Vanuatu | 170 | 155 |  | Mauritius | 170 | -59.80 (-87.22, 7.97) |  | Republic of South Sudan | 170 | 146 |  | Romania | 170 | -55.3779(-82.0465,30.3133) |
| Kingdom of Bahrain | 171 | 178 |  | Greenland | 171 | -59.83 (-79.73, -15.63) |  | Kingdom of Morocco | 170 | 174 |  | Portugal | 171 | -57.6783(-82.3174, -1.3027) |
| Federated States of Micronesia | 172 | 170 |  | Slovenia | 172 | -60.26 (-83.31, 0.90) |  | State of Qatar | 172 | 192 |  | New Zealand | 172 | -58.6314(-74.8997, -24.3792) |
| Republic of Cyprus | 173 | 174 |  | Croatia | 173 | -60.74 (-86.90, 8.15) |  | People's Democratic Republic of Algeria | 173 | 177 |  | Switzerland | 173 | -58.6830(-78.4711, -14.4329) |
| Antigua and Barbuda | 174 | 168 |  | Bosnia and Herzegovina | 174 | -61.57 (-87.69, 16.17) |  | Japan | 173 | 160 |  | Guatemala | 174 | -58.8088(-84.8481,0.6738) |
| Republic of Kiribati | 174 | 166 |  | Colombia | 175 | -62.96 (-86.34, 1.18) |  | Republic of Cyprus | 175 | 174 |  | Luxembourg | 175 | -59.3827(-81.6603, -11.8082) |
| Belize | 176 | 149 |  | Mexico | 176 | -63.40 (-84.72, -6.08) |  | Republic of Djibouti | 175 | 164 |  | Lithuania | 176 | -60.9248(-86.5932, -5.7568) |
| Independent State of Samoa | 177 | 178 |  | Chile | 177 | -64.06 (-85.06, -19.25) |  | Republic of Italy | 175 | 188 |  | China | 177 | -61.7126(-84.8440, -16.6647) |
| Republic of Djibouti | 177 | 159 |  | Ecuador | 178 | -65.82 (-87.38, -24.97) |  | Republic of Uganda | 175 | 132 |  | Norway | 178 | -61.9498(-79.2417, -26.1869) |
| Grenada | 179 | 188 |  | Serbia | 179 | -65.86 (-89.74, 5.01) |  | Republic of Kenya | 179 | 162 |  | Georgia | 179 | -62.1685(-82.3250, -18.8241) |
| Republic of Fiji | 179 | 181 |  | Kyrgyzstan | 180 | -69.03 (-84.45, -36.66) |  | State of Libya | 180 | 164 |  | Italy | 180 | -63.8453(-82.8178, -27.4121) |
| Brunei Darussalam | 179 | 183 |  | Albania | 181 | -69.41 (-91.15, -15.53) |  | Sultanate of Oman | 181 | 181 |  | Peru | 181 | -64.4211(-87.7940, -1.5020) |
| Republic of Cabo Verde | 182 | 169 |  | Puerto Rico | 182 | -70.16 (-90.80, -14.93) |  | Republic of Tunisia | 181 | 178 |  | Belarus | 182 | -65.4940(-85.4388, -18.8933) |
| Commonwealth of Dominica | 183 | 181 |  | Republic of Moldova | 183 | -71.26 (-89.44, -23.83) |  | Republic of Turkey | 183 | 183 |  | Chile | 183 | -65.5880(-85.6963, -22.6813) |
| Republic of Iraq | 184 | 172 |  | Portugal | 184 | -72.17 (-88.37, -35.10) |  | Democratic Republic of Sao Tome and Principe | 183 | 146 |  | Panama | 184 | -66.0084(-87.9831, -14.8243) |
| State of Qatar | 185 | 177 |  | Italy | 185 | -74.87 (-88.06, -49.55) |  | Republic of Fiji | 185 | 183 |  | Russian Federation | 185 | -66.5450(-82.0073, -36.6580) |
| Northern Mariana Islands | 185 | 187 |  | Russian Federation | 186 | -74.94 (-86.52, -52.55) |  | Arab Republic of Egypt | 185 | 201 |  | Spain | 186 | -67.2899(-84.9920, -27.0707) |
| Republic of the Marshall Islands | 187 | 183 |  | China | 187 | -75.18 (-90.17, -45.97) |  | Lebanese Republic | 185 | 192 |  | Mexico | 187 | -67.9494(-86.6187, -17.7541) |
| American Samoa | 188 | 189 |  | Kuwait | 188 | -76.39 (-90.24, -42.93) |  | Republic of Indonesia | 185 | 171 |  | Colombia | 188 | -68.3158(-88.3126, -13.4425) |
| Kingdom of Tonga | 188 | 186 |  | Spain | 189 | -76.50 (-89.22, -47.61) |  | Republic of Mozambique | 185 | 173 |  | Latvia | 189 | -69.1753(-88.1422, -18.4099) |
| Cook Islands | 190 | 193 |  | Romania | 190 | -76.54 (-90.56, -31.48) |  | Republic of Malta | 190 | 178 |  | Qatar | 190 | -72.8348(-92.3199, -10.0910) |
| Republic of Malta | 191 | 191 |  | Belarus | 191 | -77.84 (-90.65, -47.92) |  | Principality of Andorra | 190 | 188 |  | Armenia | 191 | -73.4599(-87.2411, -47.2813) |
| Bermuda | 192 | 197 |  | Sri Lanka | 192 | -78.44 (-94.01, -33.04) |  | Republic of Yemen | 192 | 181 |  | Estonia | 192 | -74.0635(-88.9717, -37.2475) |
| Republic of Seychelles | 192 | 199 |  | Lithuania | 193 | -80.29 (-93.24, -52.47) |  | Jamaica | 192 | 183 |  | Republic of Korea | 193 | -74.3214(-86.7644, -50.0196) |
| Democratic Republic of Sao Tome and Principe | 194 | 183 |  | Guam | 194 | -81.60 (-95.56, -11.50) |  | Republic of Zimbabwe | 194 | 174 |  | Bahrain | 194 | -75.9114(-92.0854, -26.1025) |
| Republic of Nauru | 195 | 194 |  | Georgia | 195 | -81.65 (-91.43, -60.63) |  | Principality of Monaco | 194 | 183 |  | Ecuador | 195 | -77.0710(-91.5346, -49.6608) |
| Republic of Palau | 195 | 198 |  | Seychelles | 196 | -82.24 (-94.89, -41.57) |  | Republic of San Marino | 194 | 192 |  | Kyrgyzstan | 196 | -77.4495(-88.6792, -53.8790) |
| Republic of Maldives | 195 | 194 |  | Armenia | 197 | -83.58 (-92.10, -67.38) |  | Hellenic Republic | 194 | 188 |  | Ukraine | 197 | -78.8267(-91.3271, -48.5713) |
| Palestine | 198 | 192 |  | Estonia | 198 | -83.94 (-93.17, -61.15) |  | Republic of Maldives | 198 | 196 |  | Sri Lanka | 198 | -78.6620(-94.0699, -33.7442) |
| Tuvalu | 198 | 194 |  | Latvia | 199 | -84.51 (-94.04, -58.99) |  | Republic of Singapore | 198 | 196 |  | Guam | 199 | -79.9203(-95.1547, -3.4339) |
| Republic of Niue | 200 | 201 |  | Republic of Korea | 200 | -84.94 (-92.24, -70.69) |  | Hashemite Kingdom of Jordan | 200 | 198 |  | Seychelles | 200 | -81.9202(-94.7944, -40.5180) |
| Principality of Andorra | 200 | 199 |  | Ukraine | 201 | -87.16 (-94.74, -68.80) |  | Kingdom of Saudi Arabia | 201 | 200 |  | Kuwait | 201 | -86.9483(-94.6048, -68.4511) |
| Principality of Monaco | 202 | 201 |  | Uzbekistan | 202 | -87.16 (-93.28, -76.08) |  | People's Republic of Bangladesh | 202 | 201 |  | Kazakhstan | 202 | -88.7340(-93.7815, -78.3220) |
| Republic of San Marino | 202 | 201 |  | Egypt | 203 | -87.39 (-94.29, -70.37) |  | Palestine | 202 | 203 |  | Uzbekistan | 203 | -89.7621(-94.6426, -80.9236) |
| Tokelau | 202 | 201 |  | Kazakhstan | 204 | -89.42 (-94.16, -79.64) |  | Republic of Iraq | 204 | 203 |  | Egypt | 204 | -92.7085(-96.7014, -82.8669) |

UI, uncertainty interval.

**Table S4.** Ranks of childhood sexual abuse and bullying-related DALYs, DALY rates, and the changes in adolescents and young adults of 204 countries/territories, 1990-2021

| **The rank of DALYs** | | |  | **The rank of percentage change in DALYs** | | |  | **The rank of DALY rates** | | |  | **The rank of percentage change in DALY rates** | | |
| --- | --- | --- | --- | --- | --- | --- | --- | --- | --- | --- | --- | --- | --- | --- |
| **(Descending)** | | |  | **(Descending) 1990-2021** | | |  | **(Descending)** | | |  | **(Descending) 1990-2021** | | |
| **Country or region** | **Rank** | |  | **Country or region** | **Rank** | **Value (95%UI)** |  | **Country or region** | **Rank** | |  | **Country or region** | **Rank** | **Value (95%UI)** |
|  | **1990** | **2021** |  |  |  |  |  |  | **1990** | **2021** |  |  |  |  |
| People's Republic of China | 1 | 2 |  | Niger | 1 | 611.6966(465.3376,769.2769) |  | People's Republic of China | 1 | 2 |  | Burkina Faso | 1 | 110.3523(73.1870,158.5453) |
| Republic of India | 2 | 1 |  | Equatorial Guinea | 2 | 585.5129(474.4233,757.1517) |  | Republic of India | 2 | 1 |  | Niger | 2 | 106.7803(64.2563,152.5645) |
| United States of America | 3 | 3 |  | Burkina Faso | 3 | 421.7088(329.5328,541.2355) |  | United States of America | 3 | 3 |  | Guinea | 3 | 85.0136(55.2739,116.2692) |
| People's Republic of Bangladesh | 4 | 7 |  | Chad | 4 | 413.7304(320.8620,522.9582) |  | People's Republic of Bangladesh | 4 | 7 |  | Djibouti | 4 | 79.7793(44.3248,125.2222) |
| Federative Republic of Brazil | 5 | 8 |  | Mali | 5 | 413.1962(322.6847,522.2858) |  | Federative Republic of Brazil | 5 | 8 |  | Oman | 5 | 75.6496(43.3724,127.5600) |
| Arab Republic of Egypt | 6 | 5 |  | Qatar | 6 | 405.4719(330.9794,519.9485) |  | Arab Republic of Egypt | 6 | 5 |  | Somalia | 6 | 75.1872(38.6817,111.6874) |
| Islamic Republic of Iran | 7 | 12 |  | Somalia | 7 | 400.3655(296.0994,504.6166) |  | Islamic Republic of Iran | 7 | 12 |  | Burundi | 7 | 65.6119(37.1245,97.3100) |
| Republic of Turkey | 8 | 11 |  | Guinea | 8 | 378.3287(301.4406,459.1360) |  | Republic of Turkey | 8 | 11 |  | Mozambique | 8 | 64.3446(43.5451,91.6083) |
| Republic of Indonesia | 9 | 10 |  | Benin | 9 | 352.6504(274.4609,468.7071) |  | Republic of Indonesia | 9 | 10 |  | Mali | 9 | 63.8079(34.9174,98.6283) |
| Russian Federation | 10 | 18 |  | Djibouti | 10 | 335.0964(249.2904,445.0758) |  | Russian Federation | 10 | 18 |  | Equatorial Guinea | 10 | 63.2150(36.7655,104.0808) |
| Islamic Republic of Pakistan | 11 | 4 |  | Afghanistan | 11 | 332.8471(266.4334,422.7871) |  | Islamic Republic of Pakistan | 11 | 4 |  | Chad | 11 | 60.7746(31.7109,94.9579) |
| Federal Republic of Germany | 12 | 16 |  | Burundi | 12 | 326.2642(252.9413,407.8511) |  | Federal Republic of Germany | 12 | 16 |  | Mauritania | 12 | 60.6382(28.6727,111.6011) |
| French Republic | 13 | 17 |  | Mozambique | 13 | 320.8393(267.5776,390.6536) |  | French Republic | 13 | 17 |  | Guinea-Bissau | 13 | 59.0861(35.9064,85.8781) |
| United Kingdom of Great Britain and Northern Ireland | 14 | 24 |  | Angola | 14 | 310.1628(254.4586,376.2651) |  | United Kingdom of Great Britain and Northern Ireland | 14 | 24 |  | United States of America | 14 | 56.0253(40.5091,74.2330) |
| Federal Republic of Nigeria | 15 | 6 |  | Uganda | 15 | 296.7672(245.9548,369.3963) |  | Federal Republic of Nigeria | 15 | 6 |  | Ethiopia | 15 | 55.3904(27.0647,86.8398) |
| United Mexican States | 16 | 14 |  | Cameroon | 16 | 295.8615(257.2746,340.7155) |  | United Mexican States | 16 | 14 |  | Morocco | 16 | 53.4000(17.0857,100.1571) |
| Republic of Italy | 17 | 36 |  | Ethiopia | 17 | 264.3457(197.9302,338.0855) |  | Republic of Italy | 17 | 36 |  | Saudi Arabia | 17 | 53.3520(36.2886,77.7564) |
| Republic of the Philippines | 18 | 15 |  | Mauritania | 18 | 262.4251(190.3060,377.4056) |  | Republic of the Philippines | 18 | 15 |  | Senegal | 18 | 52.2115(30.3904,76.8587) |
| Democratic Republic of the Congo | 19 | 9 |  | Gambia | 19 | 261.8132(215.1586,329.4117) |  | Democratic Republic of the Congo | 19 | 9 |  | Benin | 19 | 49.1530(23.3887,87.3949) |
| Japan | 20 | 42 |  | Liberia | 20 | 251.8666(197.7272,324.5452) |  | Japan | 20 | 42 |  | Uganda | 20 | 47.7531(28.8310,74.7996) |
| Republic of South Africa | 21 | 25 |  | Yemen | 21 | 242.7875(198.9603,312.0597) |  | Republic of South Africa | 21 | 25 |  | Eritrea | 21 | 47.7428(23.0520,79.5545) |
| People's Democratic Republic of Algeria | 22 | 23 |  | Madagascar | 22 | 241.8702(193.6195,297.4059) |  | People's Democratic Republic of Algeria | 22 | 23 |  | Canada | 22 | 46.6309(18.3835,83.9127) |
| Kingdom of Spain | 23 | 44 |  | Sierra Leone | 23 | 239.8791(197.8257,293.5422) |  | Kingdom of Spain | 23 | 44 |  | Pakistan | 23 | 45.1842(21.9784,80.1827) |
| Ukraine | 24 | 52 |  | United Arab Emirates | 24 | 239.1494(171.0725,341.4398) |  | Ukraine | 24 | 52 |  | Bhutan | 24 | 43.7038(19.5117,74.7843) |
| Argentine Republic | 25 | 28 |  | Nigeria | 25 | 233.5609(190.9493,289.8404) |  | Argentine Republic | 25 | 28 |  | Liberia | 25 | 42.2535(20.3659,71.6362) |
| Kingdom of Thailand | 26 | 46 |  | Democratic Republic of the Congo | 26 | 230.9483(193.0942,275.0878) |  | Kingdom of Thailand | 26 | 46 |  | Sierra Leone | 26 | 42.0552(24.4787,64.4842) |
| Republic of Korea | 27 | 54 |  | Guinea-Bissau | 27 | 230.7579(182.5645,286.4614) |  | Republic of Korea | 27 | 54 |  | Guatemala | 27 | 41.5847(6.1870,69.8373) |
| Socialist Republic of Viet Nam | 28 | 41 |  | Senegal | 28 | 228.7411(181.6128,281.9733) |  | Socialist Republic of Viet Nam | 28 | 41 |  | Ghana | 28 | 41.4279(10.2075,98.9835) |
| Republic of Chile | 29 | 45 |  | Jordan | 29 | 225.9726(180.2926,302.0425) |  | Republic of Chile | 29 | 45 |  | United Republic of Tanzania | 29 | 41.4183(17.6646,78.1776) |
| Federal Democratic Republic of Ethiopia | 30 | 13 |  | United Republic of Tanzania | 30 | 219.9551(166.2131,303.1222) |  | Federal Democratic Republic of Ethiopia | 30 | 13 |  | Mexico | 30 | 41.3860(27.1391,55.0078) |
| Republic of Kenya | 31 | 20 |  | Ghana | 31 | 215.4710(145.8303,343.8552) |  | Republic of Kenya | 31 | 20 |  | Turkey | 31 | 39.4046(12.6360,80.7818) |
| Republic of Colombia | 32 | 40 |  | Republic of Côte d'Ivoire | 32 | 206.1362(162.2575,264.7879) |  | Republic of Colombia | 32 | 40 |  | Egypt | 32 | 39.1378(15.0060,82.4852) |
| Republic of Sudan | 33 | 22 |  | Palestine | 33 | 204.1422(171.0256,244.8115) |  | Republic of Sudan | 33 | 22 |  | Gambia | 33 | 39.0610(21.1296,65.0421) |
| Taiwan (Province of China) | 34 | 77 |  | Togo | 34 | 202.5801(168.4923,239.2260) |  | Taiwan (Province of China) | 34 | 77 |  | Togo | 34 | 38.0308(22.4806,54.7479) |
| Canada | 35 | 37 |  | Oman | 35 | 199.7336(144.6547,288.3150) |  | Canada | 35 | 37 |  | Afghanistan | 35 | 37.2622(16.2015,65.7835) |
| Australia | 36 | 48 |  | Zambia | 36 | 196.3509(154.2032,245.4632) |  | Australia | 36 | 48 |  | Madagascar | 36 | 36.2570(17.0261,58.3916) |
| Federal Democratic Republic of Nepal | 37 | 33 |  | Pakistan | 37 | 195.8754(148.5837,267.2001) |  | Federal Democratic Republic of Nepal | 37 | 33 |  | Republic of Côte d'Ivoire | 37 | 36.0120(16.5173,62.0701) |
| Republic of Yemen | 38 | 21 |  | Malawi | 38 | 177.9780(138.6473,247.9926) |  | Republic of Yemen | 38 | 21 |  | Botswana | 38 | 35.4219(18.1524,61.8278) |
| Syrian Arab Republic | 39 | 50 |  | Eritrea | 39 | 173.2935(127.6207,232.1385) |  | Syrian Arab Republic | 39 | 50 |  | Nicaragua | 39 | 35.4209(14.8649,60.2675) |
| Republic of Uganda | 40 | 19 |  | Papua New Guinea | 40 | 172.7053(138.9951,209.0311) |  | Republic of Uganda | 40 | 19 |  | Slovakia | 40 | 35.1464(9.8520,92.2798) |
| Kingdom of Saudi Arabia | 41 | 31 |  | Iraq | 41 | 172.6429(130.1580,239.0440) |  | Kingdom of Saudi Arabia | 41 | 31 |  | Cambodia | 41 | 35.0118(9.1630,64.2232) |
| Kingdom of the Netherlands | 42 | 65 |  | Central African Republic | 42 | 171.0882(135.4467,209.0936) |  | Kingdom of the Netherlands | 42 | 65 |  | Algeria | 42 | 34.4404(15.4686,67.8015) |
| Portuguese Republic | 43 | 81 |  | Kenya | 43 | 170.1656(151.4630,193.8397) |  | Portuguese Republic | 43 | 81 |  | Iraq | 43 | 33.4295(12.6377,65.9258) |
| Kingdom of Morocco | 44 | 43 |  | Bahrain | 44 | 168.1732(147.5537,192.7469) |  | Kingdom of Morocco | 44 | 43 |  | Democratic Republic of the Congo | 44 | 33.4066(18.1475,51.1994) |
| Republic of Iraq | 45 | 30 |  | Sudan | 45 | 165.7756(133.1602,212.0291) |  | Republic of Iraq | 45 | 30 |  | Comoros | 45 | 31.4615(13.9442,52.6780) |
| Republic of Peru | 46 | 53 |  | Guatemala | 46 | 162.4791(96.8565,214.8557) |  | Republic of Peru | 46 | 53 |  | Yemen | 46 | 31.2485(14.4676,57.7718) |
| Bolivarian Republic of Venezuela | 47 | 55 |  | Timor-Leste | 47 | 150.9685(111.5596,202.3464) |  | Bolivarian Republic of Venezuela | 47 | 55 |  | Nepal | 47 | 30.6235(16.8934,48.0099) |
| Republic of Ghana | 48 | 26 |  | Saudi Arabia | 48 | 150.3781(122.5187,190.2233) |  | Republic of Ghana | 48 | 26 |  | Lao People's Democratic Republic | 48 | 30.5286(14.5570,49.9913) |
| Democratic People's Republic of Korea | 49 | 66 |  | Belize | 49 | 150.2788(109.6300,212.2545) |  | Democratic People's Republic of Korea | 49 | 66 |  | Brazil | 49 | 30.4410(14.2466,46.9071) |
| Romania | 50 | 88 |  | Vanuatu | 50 | 147.5457(112.8181,205.7946) |  | Romania | 50 | 88 |  | Ireland | 50 | 29.9027(11.0465,66.0613) |
| United Republic of Tanzania | 51 | 29 |  | Honduras | 51 | 146.2329(108.2084,203.3373) |  | United Republic of Tanzania | 51 | 29 |  | Eswatini | 51 | 28.6076(9.8916,59.0377) |
| Democratic Socialist Republic of Sri Lanka | 52 | 68 |  | Rwanda | 52 | 142.3669(109.2821,179.7072) |  | Democratic Socialist Republic of Sri Lanka | 52 | 68 |  | United Arab Emirates | 52 | 27.9651(2.2788,66.5604) |
| Republic of Angola | 53 | 27 |  | Egypt | 53 | 140.6454(98.9082,215.6168) |  | Republic of Angola | 53 | 27 |  | Myanmar | 53 | 27.8655(-9.7749,67.3327) |
| Republic of Zambia | 54 | 39 |  | Haiti | 54 | 139.9389(106.4397,178.9231) |  | Republic of Zambia | 54 | 39 |  | Rwanda | 54 | 27.1334(9.7788,46.7203) |
| Republic of Cuba | 55 | 102 |  | Kuwait | 55 | 120.4254(80.7538,198.8482) |  | Republic of Cuba | 55 | 102 |  | Costa Rica | 55 | 26.5168(1.5127,55.0721) |
| Republic of Zimbabwe | 56 | 59 |  | Sao Tome and Principe | 56 | 114.6489(93.5513,139.9677) |  | Republic of Zimbabwe | 56 | 59 |  | Indonesia | 56 | 26.2657(16.9609,37.6130) |
| Republic of the Union of Myanmar | 57 | 63 |  | Solomon Islands | 57 | 111.8806(74.2469,167.5850) |  | Republic of the Union of Myanmar | 57 | 63 |  | Central African Republic | 57 | 25.8272(9.2840,43.4676) |
| Hellenic Republic | 58 | 85 |  | Lao People's Democratic Republic | 58 | 111.1475(85.3112,142.6309) |  | Hellenic Republic | 58 | 85 |  | Mauritius | 58 | 25.4639(5.6860,57.2759) |
| Republic of Tunisia | 59 | 72 |  | Congo | 59 | 109.5647(88.3303,131.7405) |  | Republic of Tunisia | 59 | 72 |  | Palestine | 59 | 24.9657(11.3588,41.6759) |
| Kingdom of Belgium | 60 | 73 |  | Nepal | 60 | 107.2623(85.4765,134.8495) |  | Kingdom of Belgium | 60 | 73 |  | Vanuatu | 60 | 24.9132(7.3895,54.3060) |
| Republic of Poland | 61 | 84 |  | South Sudan | 61 | 101.5033(63.0205,148.2809) |  | Republic of Poland | 61 | 84 |  | Cabo Verde | 61 | 24.8601(12.5837,38.4512) |
| Malaysia | 62 | 58 |  | Gabon | 62 | 97.7735(78.4522,117.2138) |  | Malaysia | 62 | 58 |  | Zambia | 62 | 24.5127(6.8042,45.1474) |
| Republic of Madagascar | 63 | 35 |  | Botswana | 63 | 96.2952(71.2630,134.5708) |  | Republic of Madagascar | 63 | 35 |  | Haiti | 63 | 24.5069(7.1238,44.7362) |
| Islamic Republic of Afghanistan | 64 | 32 |  | Cambodia | 64 | 94.9338(57.6126,137.1102) |  | Islamic Republic of Afghanistan | 64 | 32 |  | Poland | 64 | 24.4739(11.5751,37.4135) |
| Republic of Belarus | 65 | 101 |  | Nicaragua | 65 | 94.2203(64.7390,129.8553) |  | Republic of Belarus | 65 | 101 |  | Angola | 65 | 24.4736(7.5688,44.5339) |
| Republic of Malawi | 66 | 47 |  | Paraguay | 66 | 93.5472(70.6227,114.1038) |  | Republic of Malawi | 66 | 47 |  | Kenya | 66 | 24.1708(15.5749,35.0517) |
| Republic of Cameroon | 67 | 34 |  | Comoros | 67 | 87.9733(62.9258,118.3102) |  | Republic of Cameroon | 67 | 34 |  | Greece | 67 | 23.6590(9.1957,48.8221) |
| Republic of Ecuador | 68 | 67 |  | United States of America | 68 | 82.7368(64.5643,104.0616) |  | Republic of Ecuador | 68 | 67 |  | Tunisia | 68 | 23.5728(11.0985,44.6789) |
| Republic of Austria | 69 | 91 |  | Philippines | 69 | 78.9399(64.5276,95.8370) |  | Republic of Austria | 69 | 91 |  | Malawi | 69 | 23.4715(6.0018,54.5705) |
| Kingdom of Sweden | 70 | 94 |  | Ecuador | 70 | 77.7329(56.4887,108.2308) |  | Kingdom of Sweden | 70 | 94 |  | Tuvalu | 70 | 23.4127(4.6375,59.1082) |
| Republic of Mozambique | 71 | 38 |  | Namibia | 71 | 77.7109(60.1539,100.7894) |  | Republic of Mozambique | 71 | 38 |  | Iran (Islamic Republic of) | 71 | 23.3450(15.7170,35.5065) |
| Swiss Confederation | 72 | 87 |  | Morocco | 72 | 77.0453(35.1335,131.0096) |  | Swiss Confederation | 72 | 87 |  | Sao Tome and Principe | 72 | 23.0851(10.9872,37.6035) |
| Republic of Côte d'Ivoire | 73 | 49 |  | Malaysia | 73 | 75.5472(41.7604,126.0609) |  | Republic of Côte d'Ivoire | 73 | 49 |  | Cameroon | 73 | 22.8958(10.9164,36.8208) |
| Hashemite Kingdom of Jordan | 74 | 51 |  | Tuvalu | 74 | 74.7028(48.1247,125.2333) |  | Hashemite Kingdom of Jordan | 74 | 51 |  | Timor-Leste | 74 | 22.0585(2.8919,47.0461) |
| Republic of Rwanda | 75 | 64 |  | India | 75 | 73.9620(53.6390,93.9025) |  | Republic of Rwanda | 75 | 64 |  | Guyana | 75 | 22.0002(1.2798,53.3675) |
| State of Israel | 76 | 78 |  | Bolivia (Plurinational State of) | 76 | 72.8919(45.8099,109.5694) |  | State of Israel | 76 | 78 |  | Kuwait | 76 | 21.8536(-0.0773,65.2066) |
| State of Libya | 77 | 86 |  | Eswatini | 77 | 71.7058(46.7178,112.3334) |  | State of Libya | 77 | 86 |  | Colombia | 77 | 21.4649(3.9465,45.8864) |
| Republic of Uzbekistan | 78 | 97 |  | Israel | 78 | 71.4551(51.5768,98.8069) |  | Republic of Uzbekistan | 78 | 97 |  | Lebanon | 78 | 21.1561(-7.6520,60.7679) |
| Dominican Republic | 79 | 90 |  | Tajikistan | 79 | 70.1845(32.5194,112.4874) |  | Dominican Republic | 79 | 90 |  | El Salvador | 79 | 21.0954(-0.9349,51.3256) |
| Republic of Bulgaria | 80 | 123 |  | Panama | 80 | 65.6793(48.2838,86.9542) |  | Republic of Bulgaria | 80 | 123 |  | Nigeria | 80 | 20.7966(5.3651,41.1778) |
| Republic of Nicaragua | 81 | 76 |  | Lebanon | 81 | 65.4714(26.1261,119.5720) |  | Republic of Nicaragua | 81 | 76 |  | Saint Lucia | 81 | 20.6223(4.1905,50.0264) |
| Republic of Lithuania | 82 | 130 |  | Cabo Verde | 82 | 64.0677(47.9364,81.9266) |  | Republic of Lithuania | 82 | 130 |  | Paraguay | 82 | 19.9571(5.7489,32.6977) |
| Republic of Kazakhstan | 83 | 111 |  | Brunei Darussalam | 83 | 63.5399(40.6382,97.4325) |  | Republic of Kazakhstan | 83 | 111 |  | Sudan | 83 | 19.5974(4.9206,40.4112) |
| Republic of Guatemala | 84 | 69 |  | Canada | 84 | 62.6440(31.3117,103.9973) |  | Republic of Guatemala | 84 | 69 |  | Saint Vincent and the Grenadines | 84 | 19.4373(4.8258,39.6884) |
| Republic of Haiti | 85 | 75 |  | Mexico | 85 | 61.4468(45.1785,77.0013) |  | Republic of Haiti | 85 | 75 |  | Ecuador | 85 | 19.2393(4.9868,39.7001) |
| Plurinational State of Bolivia | 86 | 95 |  | Algeria | 86 | 60.5338(37.8797,100.3699) |  | Plurinational State of Bolivia | 86 | 95 |  | Honduras | 86 | 18.5321(0.2278,46.0211) |
| Kingdom of Denmark | 87 | 114 |  | Kiribati | 87 | 60.2077(32.4751,95.2622) |  | Kingdom of Denmark | 87 | 114 |  | Venezuela (Bolivarian Republic of) | 87 | 18.4433(4.6102,32.9716) |
| Republic of Finland | 88 | 110 |  | Zimbabwe | 88 | 57.8654(41.0070,79.6048) |  | Republic of Finland | 88 | 110 |  | Belize | 88 | 18.4408(-0.7956,47.7699) |
| Republic of Serbia | 89 | 122 |  | Costa Rica | 89 | 50.2073(20.5212,84.1096) |  | Republic of Serbia | 89 | 122 |  | South Sudan | 89 | 17.5849(-4.8712,44.8815) |
| New Zealand | 90 | 103 |  | Myanmar | 90 | 49.1758(5.2621,95.2206) |  | New Zealand | 90 | 103 |  | Saint Kitts and Nevis | 90 | 17.5516(0.5021,47.7050) |
| Ireland | 91 | 105 |  | Bangladesh | 91 | 47.9949(29.2930,71.5032) |  | Ireland | 91 | 105 |  | Thailand | 91 | 17.5267(-2.7731,43.1038) |
| Republic of Senegal | 92 | 70 |  | Indonesia | 92 | 46.8512(36.0293,60.0484) |  | Republic of Senegal | 92 | 70 |  | Brunei Darussalam | 92 | 17.4819(1.0300,41.8293) |
| Republic of South Sudan | 93 | 92 |  | Turkey | 93 | 46.2098(18.1345,89.6069) |  | Republic of South Sudan | 93 | 92 |  | Nauru | 93 | 17.1688(-0.8334,44.6609) |
| Republic of the Congo | 94 | 89 |  | Lesotho | 94 | 46.0377(32.3463,62.1151) |  | Republic of the Congo | 94 | 89 |  | Papua New Guinea | 94 | 16.9311(2.4768,32.5069) |
| Kingdom of Norway | 95 | 112 |  | Luxembourg | 95 | 44.3492(13.7859,88.0943) |  | Kingdom of Norway | 95 | 112 |  | Jordan | 95 | 16.6695(0.3200,43.8958) |
| Republic of Moldova | 96 | 137 |  | Colombia | 96 | 41.9874(21.5090,70.5351) |  | Republic of Moldova | 96 | 137 |  | Germany | 96 | 16.6195(-1.4467,41.3268) |
| Czech Republic | 97 | 131 |  | Argentina | 97 | 41.5577(12.6000,79.9436) |  | Czech Republic | 97 | 131 |  | Malaysia | 97 | 16.5317(-5.8967,50.0637) |
| Federal Republic of Somalia | 98 | 56 |  | Maldives | 98 | 40.4729(21.2453,74.1707) |  | Federal Republic of Somalia | 98 | 56 |  | Libya | 98 | 16.3873(2.6668,37.0957) |
| Kingdom of Cambodia | 99 | 99 |  | Libya | 99 | 37.9148(21.6565,62.4536) |  | Kingdom of Cambodia | 99 | 99 |  | Solomon Islands | 99 | 15.9908(-4.6112,46.4853) |
| Palestine | 100 | 80 |  | Brazil | 100 | 35.9251(19.0498,53.0835) |  | Palestine | 100 | 80 |  | Armenia | 100 | 15.8724(-8.9292,44.6057) |
| Burkina Faso | 101 | 57 |  | South Africa | 101 | 33.9820(24.8446,45.1592) |  | Burkina Faso | 101 | 57 |  | Romania | 101 | 15.8263(-5.7169,48.9009) |
| Hungary | 102 | 132 |  | Dominican Republic | 102 | 33.6956(19.8549,49.7808) |  | Hungary | 102 | 132 |  | Cyprus | 102 | 15.7996(6.1720,27.5214) |
| Republic of Honduras | 103 | 93 |  | New Zealand | 103 | 33.5100(14.4385,53.8027) |  | Republic of Honduras | 103 | 93 |  | Grenada | 103 | 15.6195(1.7309,35.1955) |
| Republic of Mali | 104 | 60 |  | Nauru | 104 | 32.6121(12.2372,63.7278) |  | Republic of Mali | 104 | 60 |  | Argentina | 104 | 15.3485(-8.2478,46.6273) |
| Republic of Chad | 105 | 62 |  | Cyprus | 105 | 31.8537(20.8913,45.2005) |  | Republic of Chad | 105 | 62 |  | Uruguay | 105 | 15.3351(-5.7072,44.0432) |
| Republic of El Salvador | 106 | 115 |  | Bahamas | 106 | 31.7029(14.3250,57.6181) |  | Republic of El Salvador | 106 | 115 |  | Syrian Arab Republic | 106 | 14.9664(5.4161,24.0796) |
| Republic of Burundi | 107 | 71 |  | Ireland | 107 | 31.1392(12.1035,67.6419) |  | Republic of Burundi | 107 | 71 |  | Belgium | 107 | 14.3923(-3.9893,42.2624) |
| Republic of Paraguay | 108 | 100 |  | Bhutan | 108 | 29.2827(7.5184,57.2442) |  | Republic of Paraguay | 108 | 100 |  | Austria | 108 | 14.0413(-4.1048,45.2816) |
| Togolese Republic | 109 | 83 |  | Suriname | 109 | 29.0669(11.3637,52.3861) |  | Togolese Republic | 109 | 83 |  | Lesotho | 109 | 14.0185(3.3289,26.5708) |
| Lebanese Republic | 110 | 109 |  | Peru | 110 | 28.3065(13.7640,41.1401) |  | Lebanese Republic | 110 | 109 |  | Panama | 110 | 13.5768(1.6517,28.1612) |
| Independent State of Papua New Guinea | 111 | 96 |  | Antigua and Barbuda | 111 | 23.0728(10.6797,40.1672) |  | Independent State of Papua New Guinea | 111 | 96 |  | Namibia | 111 | 13.1108(1.9360,27.8001) |
| Bosnia and Herzegovina | 112 | 147 |  | Kyrgyzstan | 112 | 22.8150(-5.8173,42.6668) |  | Bosnia and Herzegovina | 112 | 147 |  | Bangladesh | 112 | 12.7863(-1.4663,30.7018) |
| Republic of Sierra Leone | 113 | 82 |  | Syrian Arab Republic | 113 | 22.7140(12.5201,32.4413) |  | Republic of Sierra Leone | 113 | 82 |  | Fiji | 113 | 12.7846(-2.3183,35.0049) |
|  |  |  |  |  |  |  |  |  |  |  |  |  |  |  |
| Central African Republic | 115 | 98 |  | Uzbekistan | 115 | 22.2907(0.5001,42.3798) |  | Central African Republic | 115 | 98 |  | India | 115 | 12.7152(-0.4527,25.6353) |
| Lao People's Democratic Republic | 116 | 107 |  | Monaco | 116 | 22.1079(12.1058,33.6407) |  | Lao People's Democratic Republic | 116 | 107 |  | Cook Islands | 116 | 12.2669(-7.4730,44.9025) |
| Republic of Guinea | 117 | 74 |  | Mongolia | 117 | 19.9496(-3.4849,57.3093) |  | Republic of Guinea | 117 | 74 |  | Dominica | 117 | 11.9842(-3.3588,35.4049) |
| Republic of Benin | 118 | 79 |  | El Salvador | 118 | 19.1502(-2.5262,48.8948) |  | Republic of Benin | 118 | 79 |  | Japan | 118 | 11.9806(1.5055,25.5058) |
| Republic of Singapore | 119 | 140 |  | Iran (Islamic Republic of) | 119 | 18.5631(11.2309,30.2531) |  | Republic of Singapore | 119 | 140 |  | Qatar | 119 | 11.9345(-4.5615,37.2849) |
| Republic of the Niger | 120 | 61 |  | Venezuela (Bolivarian Republic of) | 120 | 17.1623(3.4789,31.5335) |  | Republic of the Niger | 120 | 61 |  | Mongolia | 120 | 11.8991(-9.9626,46.7514) |
| Puerto Rico | 121 | 144 |  | Tunisia | 121 | 16.0166(4.3050,35.8321) |  | Puerto Rico | 121 | 144 |  | Russian Federation | 121 | 11.7446(-2.1971,22.4517) |
| Republic of Latvia | 122 | 148 |  | Turkmenistan | 122 | 15.6859(-1.6578,35.1941) |  | Republic of Latvia | 122 | 148 |  | Dominican Republic | 122 | 11.4541(-0.0840,24.8634) |
| Eastern Republic of Uruguay | 123 | 133 |  | Fiji | 123 | 13.5119(-1.6884,35.8755) |  | Eastern Republic of Uruguay | 123 | 133 |  | Zimbabwe | 123 | 11.1074(-0.7578,26.4078) |
| Kingdom of Lesotho | 124 | 124 |  | Switzerland | 124 | 12.7855(-4.7744,42.1150) |  | Kingdom of Lesotho | 124 | 124 |  | Suriname | 124 | 10.9518(-4.2667,30.9980) |
| Republic of Croatia | 125 | 145 |  | Saint Kitts and Nevis | 125 | 12.7461(-3.6064,41.6668) |  | Republic of Croatia | 125 | 145 |  | New Zealand | 125 | 10.8999(-4.9418,27.7560) |
| State of Eritrea | 126 | 106 |  | Grenada | 126 | 11.8250(-1.6078,30.7585) |  | State of Eritrea | 126 | 106 |  | Republic of Korea | 126 | 10.7428(-3.6013,28.1170) |
| Republic of Costa Rica | 127 | 128 |  | Belgium | 127 | 11.6274(-6.3099,38.8239) |  | Republic of Costa Rica | 127 | 128 |  | South Africa | 127 | 10.4327(2.9013,19.6453) |
| Republic of Namibia | 128 | 117 |  | Marshall Islands | 128 | 9.5247(-1.7320,21.6015) |  | Republic of Namibia | 128 | 117 |  | Finland | 128 | 10.3990(-5.3079,33.7782) |
| Republic of Azerbaijan | 129 | 138 |  | Norway | 129 | 9.1478(1.3307,16.7422) |  | Republic of Azerbaijan | 129 | 138 |  | Albania | 129 | 10.3311(-1.2622,21.3071) |
| Republic of Panama | 130 | 125 |  | Iceland | 130 | 9.0232(-10.6208,39.8186) |  | Republic of Panama | 130 | 125 |  | Switzerland | 130 | 10.2961(-6.8763,38.9783) |
| Slovak Republic | 131 | 142 |  | Seychelles | 131 | 8.3263(-4.4362,29.3200) |  | Slovak Republic | 131 | 142 |  | Seychelles | 131 | 10.2827(-2.7103,31.6555) |
| Sultanate of Oman | 132 | 108 |  | Uruguay | 132 | 8.2649(-11.4874,35.2133) |  | Sultanate of Oman | 132 | 108 |  | Tonga | 132 | 9.5948(-5.3331,31.9025) |
| Mongolia | 133 | 134 |  | Samoa | 133 | 7.9744(-1.4956,18.1545) |  | Mongolia | 133 | 134 |  | Georgia | 133 | 9.4340(-8.5799,26.9498) |
|  |  |  |  |  |  |  |  |  |  |  |  |  |  |  |
| Republic of Albania | 135 | 153 |  | Andorra | 135 | 6.2803(-3.7565,16.2039) |  | Republic of Albania | 135 | 153 |  | Hungary | 135 | 9.2382(-9.9693,40.6580) |
| Gabonese Republic | 136 | 119 |  | Azerbaijan | 136 | 4.6863(-13.5615,22.4315) |  | Gabonese Republic | 136 | 119 |  | Barbados | 136 | 9.2197(-8.1854,33.0690) |
| Republic of Liberia | 137 | 104 |  | Finland | 137 | 3.9984(-10.7979,26.0221) |  | Republic of Liberia | 137 | 104 |  | North Macedonia | 137 | 9.1950(-12.5365,44.6399) |
| Kyrgyz Republic | 138 | 139 |  | Tonga | 138 | 1.9435(-11.9422,22.6938) |  | Kyrgyz Republic | 138 | 139 |  | Antigua and Barbuda | 138 | 8.6155(-2.3218,23.7018) |
| State of Kuwait | 139 | 126 |  | Mauritius | 139 | -0.1102(-15.8566,25.2174) |  | State of Kuwait | 139 | 126 |  | Micronesia (Federated States of) | 139 | 8.4646(-3.9003,22.7565) |
| Republic of Estonia | 140 | 156 |  | Jamaica | 140 | -0.1701(-12.9597,16.4940) |  | Republic of Estonia | 140 | 156 |  | Niue | 140 | 8.1928(-5.0326,23.3160) |
| Turkmenistan | 141 | 143 |  | Sweden | 141 | -0.2788(-9.1839,10.6808) |  | Turkmenistan | 141 | 143 |  | Viet Nam | 141 | 8.1733(-2.0028,20.5982) |
| Georgia | 142 | 160 |  | Sri Lanka | 142 | -0.2858(-16.6205,23.8477) |  | Georgia | 142 | 160 |  | Trinidad and Tobago | 142 | 7.8330(-12.0642,34.8662) |
| United Arab Emirates | 143 | 113 |  | France | 143 | -0.3062(-19.2598,30.8878) |  | United Arab Emirates | 143 | 113 |  | Gabon | 143 | 7.6400(-2.8758,18.2206) |
| Republic of Guyana | 144 | 150 |  | Austria | 144 | -0.9362(-16.6991,26.2012) |  | Republic of Guyana | 144 | 150 |  | Jamaica | 144 | 7.3887(-6.3693,25.3146) |
| Republic of Mauritius | 145 | 151 |  | Micronesia (Federated States of) | 145 | -1.0521(-12.3322,11.9857) |  | Republic of Mauritius | 145 | 151 |  | Bahamas | 145 | 7.3436(-6.8202,28.4656) |
| Islamic Republic of Mauritania | 146 | 116 |  | Germany | 146 | -1.1615(-16.4732,19.7786) |  | Islamic Republic of Mauritania | 146 | 116 |  | Bolivia (Plurinational State of) | 146 | 7.0939(-9.6815,29.8129) |
| Republic of Tajikistan | 147 | 141 |  | Democratic People's Republic of Korea | 147 | -1.4756(-10.5152,8.9272) |  | Republic of Tajikistan | 147 | 141 |  | Malta | 147 | 6.8355(-6.3960,26.5281) |
| Republic of Slovenia | 148 | 163 |  | Guyana | 148 | -1.6845(-18.3823,23.5933) |  | Republic of Slovenia | 148 | 163 |  | United States Virgin Islands | 148 | 6.1292(-4.6540,18.8083) |
| Republic of the Gambia | 149 | 121 |  | San Marino | 149 | -2.5700(-10.3262,6.1289) |  | Republic of the Gambia | 149 | 121 |  | Northern Mariana Islands | 149 | 4.3524(-6.4357,16.3403) |
| North Macedonia | 150 | 161 |  | Saint Lucia | 150 | -3.4680(-16.6181,20.0636) |  | North Macedonia | 150 | 161 |  | Palau | 150 | 3.6416(-5.2610,13.1374) |
| Republic of Trinidad and Tobago | 151 | 159 |  | American Samoa | 151 | -5.1563(-13.6927,3.1124) |  | Republic of Trinidad and Tobago | 151 | 159 |  | Israel | 151 | 3.6106(-8.4019,20.1394) |
| Republic of Fiji | 152 | 155 |  | Northern Mariana Islands | 152 | -5.6398(-15.3949,5.2002) |  | Republic of Fiji | 152 | 155 |  | Estonia | 152 | 3.2936(-15.8130,37.9579) |
| Republic of Cyprus | 153 | 152 |  | Netherlands | 153 | -6.5782(-24.4958,12.8682) |  | Republic of Cyprus | 153 | 152 |  | Bahrain | 153 | 3.2604(-4.6792,12.7225) |
| Kingdom of Bahrain | 154 | 136 |  | Barbados | 154 | -7.6341(-22.3534,12.5350) |  | Kingdom of Bahrain | 154 | 136 |  | France | 154 | 3.1354(-16.4725,35.4063) |
| Kingdom of Eswatini | 155 | 146 |  | Guam | 155 | -8.3996(-17.3287,1.3430) |  | Kingdom of Eswatini | 155 | 146 |  | Australia | 155 | 2.9015(-10.6458,18.6283) |
| State of Qatar | 156 | 118 |  | Chile | 156 | -9.9554(-26.9986,10.1174) |  | State of Qatar | 156 | 118 |  | Tokelau | 156 | 2.4154(-6.6028,13.4206) |
| Republic of Armenia | 157 | 166 |  | United Kingdom | 157 | -10.4396(-20.4714,0.6694) |  | Republic of Armenia | 157 | 166 |  | Congo | 157 | 2.4105(-7.9664,13.2474) |
| Republic of Guinea-Bissau | 158 | 135 |  | Greenland | 158 | -10.9552(-23.0034,6.1969) |  | Republic of Guinea-Bissau | 158 | 135 |  | American Samoa | 158 | 2.4045(-6.8125,11.3324) |
| Kingdom of Bhutan | 159 | 157 |  | Slovakia | 159 | -11.2265(-27.8416,26.3027) |  | Kingdom of Bhutan | 159 | 157 |  | Latvia | 159 | 2.3122(-16.0784,33.0677) |
| Republic of Suriname | 160 | 165 |  | Denmark | 160 | -12.8247(-27.6139,1.5572) |  | Republic of Suriname | 160 | 165 |  | Spain | 160 | 2.1614(-20.2110,28.9245) |
| Democratic Republic of Timor-Leste | 161 | 149 |  | Trinidad and Tobago | 161 | -13.8848(-29.7746,7.7039) |  | Democratic Republic of Timor-Leste | 161 | 149 |  | Samoa | 161 | 1.7777(-7.1489,11.3735) |
| Grand Duchy of Luxembourg | 162 | 164 |  | Saint Vincent and the Grenadines | 162 | -15.2041(-25.5777, -0.8266) |  | Grand Duchy of Luxembourg | 162 | 164 |  | Netherlands | 162 | 1.6841(-17.8182,22.8503) |
| Union of the Comoros | 163 | 158 |  | Poland | 163 | -15.5024(-24.2586, -6.7185) |  | Union of the Comoros | 163 | 158 |  | Tajikistan | 163 | 1.4980(-20.9655,26.7273) |
| Republic of Equatorial Guinea | 164 | 129 |  | Tokelau | 164 | -16.2544(-23.6286, -7.2554) |  | Republic of Equatorial Guinea | 164 | 129 |  | Puerto Rico | 164 | 1.3637(-9.7152,12.0661) |
| Republic of Cabo Verde | 165 | 162 |  | Russian Federation | 165 | -16.2958(-26.7390, -8.2754) |  | Republic of Cabo Verde | 165 | 162 |  | Norway | 165 | 1.3268(-5.9301,8.3770) |
| Montenegro | 166 | 174 |  | Malta | 166 | -16.9714(-27.2544, -1.6671) |  | Montenegro | 166 | 174 |  | Kiribati | 166 | 1.2787(-16.2531,23.4391) |
| Republic of Maldives | 167 | 169 |  | Kazakhstan | 167 | -17.1208(-34.1657, -1.5090) |  | Republic of Maldives | 167 | 169 |  | Iceland | 167 | 1.0826(-17.1306,29.6351) |
| Republic of Malta | 168 | 175 |  | Greece | 168 | -18.5361(-28.0642, -1.9592) |  | Republic of Malta | 168 | 175 |  | Croatia | 168 | 0.9947(-20.9164,35.8394) |
| Republic of Djibouti | 169 | 154 |  | Dominica | 169 | -19.1711(-30.2455, -2.2664) |  | Republic of Djibouti | 169 | 154 |  | Maldives | 169 | 0.7994(-12.9978,24.9800) |
| Commonwealth of the Bahamas | 170 | 172 |  | North Macedonia | 170 | -21.0178(-36.7365,4.6200) |  | Commonwealth of the Bahamas | 170 | 172 |  | Monaco | 170 | 0.7251(-7.5255,10.2384) |
| Republic of Iceland | 171 | 176 |  | Palau | 171 | -22.2483(-28.9271, -15.1246) |  | Republic of Iceland | 171 | 176 |  | Marshall Islands | 171 | 0.7241(-9.6281,11.8304) |
| Brunei Darussalam | 172 | 171 |  | Cook Islands | 172 | -22.2844(-35.9492,0.3072) |  | Brunei Darussalam | 172 | 171 |  | San Marino | 172 | 0.3829(-7.6084,9.3455) |
| Solomon Islands | 173 | 170 |  | Thailand | 173 | -22.9101(-36.2255, -6.1332) |  | Solomon Islands | 173 | 170 |  | Azerbaijan | 173 | 0.3650(-17.1295,17.3777) |
| Independent State of Samoa | 174 | 177 |  | Montenegro | 174 | -26.0044(-33.2090, -18.9569) |  | Independent State of Samoa | 174 | 177 |  | Montenegro | 174 | 0.0660(-9.6770,9.5965) |
| Belize | 175 | 167 |  | Spain | 175 | -26.6123(-42.6835, -7.3869) |  | Belize | 175 | 167 |  | Guam | 175 | -0.0527(-9.7954,10.5777) |
| Republic of Vanuatu | 176 | 168 |  | Singapore | 176 | -28.1083(-36.9047, -18.8800) |  | Republic of Vanuatu | 176 | 168 |  | Democratic People's Republic of Korea | 176 | -0.5823(-9.7039,9.9147) |
| Greenland | 177 | 178 |  | Armenia | 177 | -28.2980(-43.6453, -10.5177) |  | Greenland | 177 | 178 |  | Turkmenistan | 177 | -0.5979(-15.5003,16.1643) |
| Guam | 178 | 180 |  | Japan | 178 | -29.9558(-36.5080, -21.4958) |  | Guam | 178 | 180 |  | Czechia | 178 | -0.6952(-15.7948,19.7474) |
| Kingdom of Tonga | 179 | 179 |  | Bermuda | 179 | -30.5128(-38.7523, -22.0570) |  | Kingdom of Tonga | 179 | 179 |  | Andorra | 179 | -0.8878(-10.2477,8.3665) |
| Saint Lucia | 180 | 181 |  | Serbia | 180 | -30.6383(-37.9917, -23.2210) |  | Saint Lucia | 180 | 181 |  | Sri Lanka | 180 | -1.3338(-17.4968,22.5460) |
| Democratic Republic of Sao Tome and Principe | 181 | 173 |  | Hungary | 181 | -31.1696(-43.2722, -11.3722) |  | Democratic Republic of Sao Tome and Principe | 181 | 173 |  | Peru | 181 | -1.4054(-12.5802,8.4564) |
| Saint Vincent and the Grenadines | 182 | 183 |  | Niue | 182 | -32.9278(-41.1267, -23.5525) |  | Saint Vincent and the Grenadines | 182 | 183 |  | Uzbekistan | 182 | -2.4661(-19.8453,13.5562) |
| Barbados | 183 | 187 |  | Czechia | 183 | -34.7195(-44.6456, -21.2810) |  | Barbados | 183 | 187 |  | Luxembourg | 183 | -2.5384(-23.1741,26.9973) |
| Federated States of Micronesia | 184 | 186 |  | Republic of Korea | 184 | -35.0522(-43.4646, -24.8626) |  | Federated States of Micronesia | 184 | 186 |  | Ukraine | 184 | -3.2428(-13.7567,7.2211) |
| Republic of Seychelles | 185 | 185 |  | Croatia | 185 | -35.2684(-49.3121, -12.9351) |  | Republic of Seychelles | 185 | 185 |  | Portugal | 185 | -3.3191(-18.5734,27.9720) |
| Grenada | 186 | 184 |  | Italy | 186 | -35.8264(-41.5487, -28.0447) |  | Grenada | 186 | 184 |  | Republic of Moldova | 186 | -4.0429(-13.0782,5.1523) |
| United States Virgin Islands | 187 | 193 |  | Estonia | 187 | -36.0478(-47.8773, -14.5861) |  | United States Virgin Islands | 187 | 193 |  | Belarus | 187 | -4.3268(-13.8634,5.9232) |
| Republic of Kiribati | 188 | 182 |  | Portugal | 188 | -36.4259(-46.4566, -15.8500) |  | Republic of Kiribati | 188 | 182 |  | Slovenia | 188 | -5.2402(-27.2115,28.9255) |
| Commonwealth of Dominica | 189 | 190 |  | Slovenia | 189 | -36.7400(-51.4077, -13.9315) |  | Commonwealth of Dominica | 189 | 190 |  | Bermuda | 189 | -5.5794(-16.7754,5.9105) |
| Principality of Andorra | 190 | 188 |  | Puerto Rico | 190 | -36.9093(-43.8050, -30.2479) |  | Principality of Andorra | 190 | 188 |  | Serbia | 190 | -6.0709(-16.0288,3.9736) |
| Antigua and Barbuda | 191 | 189 |  | Belarus | 191 | -38.5609(-44.6850, -31.9785) |  | Antigua and Barbuda | 191 | 189 |  | Sweden | 191 | -6.9559(-15.2648,3.2699) |
| Republic of the Marshall Islands | 192 | 191 |  | Romania | 192 | -39.1000(-50.4272, -21.7099) |  | Republic of the Marshall Islands | 192 | 191 |  | Lithuania | 192 | -7.4383(-20.0276,5.0571) |
| American Samoa | 193 | 194 |  | Albania | 193 | -40.4713(-46.7264, -34.5492) |  | American Samoa | 193 | 194 |  | Italy | 193 | -7.6609(-15.8947,3.5362) |
| Saint Kitts and Nevis | 194 | 192 |  | Ukraine | 194 | -41.3094(-47.6869, -34.9622) |  | Saint Kitts and Nevis | 194 | 192 |  | Bulgaria | 194 | -7.8631(-19.6794,6.8847) |
| Northern Mariana Islands | 195 | 195 |  | China | 195 | -46.0220(-55.8711, -38.7874) |  | Northern Mariana Islands | 195 | 195 |  | Denmark | 195 | -9.2379(-24.6356,5.7358) |
| Bermuda | 196 | 198 |  | Taiwan (Province of China) | 196 | -46.3119(-58.2787, -30.6529) |  | Bermuda | 196 | 198 |  | Kyrgyzstan | 196 | -10.5719(-31.4205,3.8833) |
| Republic of San Marino | 197 | 196 |  | Georgia | 197 | -46.9244(-55.6612, -38.4292) |  | Republic of San Marino | 197 | 196 |  | Kazakhstan | 197 | -11.7478(-29.8976,4.8762) |
| Principality of Monaco | 198 | 197 |  | United States Virgin Islands | 198 | -47.3609(-52.7093, -41.0722) |  | Principality of Monaco | 198 | 197 |  | United Kingdom | 198 | -12.2517(-22.0805, -1.3675) |
| Cook Islands | 199 | 199 |  | Latvia | 199 | -48.5700(-57.8146, -33.1100) |  | Cook Islands | 199 | 199 |  | Taiwan (Province of China) | 199 | -12.7339(-32.1849,12.7188) |
| Republic of Palau | 200 | 201 |  | Republic of Moldova | 200 | -49.0486(-53.8461, -44.1660) |  | Republic of Palau | 200 | 201 |  | Chile | 200 | -13.7855(-30.1038,5.4335) |
| Republic of Nauru | 201 | 200 |  | Cuba | 201 | -49.8970(-57.5718, -43.3957) |  | Republic of Nauru | 201 | 200 |  | Bosnia and Herzegovina | 201 | -15.7750(-26.7524, -2.9393) |
| Tuvalu | 202 | 202 |  | Bulgaria | 202 | -51.4699(-57.6938, -43.7020) |  | Tuvalu | 202 | 202 |  | China | 202 | -16.7498(-31.9401, -5.5919) |
| Republic of Niue | 203 | 203 |  | Lithuania | 203 | -53.3136(-59.6634, -47.0111) |  | Republic of Niue | 203 | 203 |  | Singapore | 203 | -18.1194(-28.1380, -7.6089) |
| Tokelau | 204 | 204 |  | Bosnia and Herzegovina | 204 | -60.3431(-65.5118, -54.2995) |  | Tokelau | 204 | 204 |  | Cuba | 204 | -20.1627(-32.3921, -9.8031) |

DALYs, disability-adjusted life years; DALY, disability-adjusted life year; UI, uncertainty interval.

**Table S5.** EAPC ranks of childhood sexual abuse and bullying-related death and DALY rates in adolescents and young adults of 204 countries/territories, 1990-2021

| **EAPC of death rate(95%CI)** | | |  | **EAPC of DALY rate(95%CI)** | | |
| --- | --- | --- | --- | --- | --- | --- |
| **(Descending)** | | |  | **(Descending)** | | |
| **Country or region** | **Rank** | **Value (95%CI)** |  | **Country or region** | **Rank** | **Value (95%CI)** |
|  | **1990-2021** |  |  |  | **1990-2021** |  |
| United States Virgin Islands | 1 | 4.0501(3.3176,4.7878) |  | Burkina Faso | 1 | 2.8537(2.6360,3.0719) |
| Republic of Liberia | 2 | 3.0737(2.3960,3.7559) |  | Republic of the Niger | 2 | 2.5739(2.4143,2.7338) |
| Kingdom of Lesotho | 3 | 3.0443(2.5223,3.5689) |  | Republic of Guinea | 3 | 2.2852(2.1914,2.3791) |
| Canada | 4 | 2.5894(2.1449,3.0359) |  | Republic of Djibouti | 4 | 2.2348(2.0900,2.3798) |
| United States of America | 5 | 2.4994(2.0744,2.9262) |  | Sultanate of Oman | 5 | 1.8158(1.5616,2.0707) |
| Republic of Sierra Leone | 6 | 2.4782(2.0832,2.8746) |  | Federal Republic of Somalia | 6 | 1.7941(1.7311,1.8572) |
| Burkina Faso | 7 | 2.2190(1.8323,2.6072) |  | Republic of Mozambique | 7 | 1.7845(1.7228,1.8463) |
| Republic of Zimbabwe | 8 | 2.1098(1.5824,2.6399) |  | Federal Democratic Republic of Ethiopia | 8 | 1.7321(1.4979,1.9668) |
| Republic of Chad | 9 | 1.9952(1.4828,2.5102) |  | Republic of Burundi | 9 | 1.7224(1.6867,1.7581) |
| Democratic Republic of Sao Tome and Principe | 10 | 1.9693(1.3832,2.5588) |  | Malaysia | 10 | 1.6653(1.1822,2.1506) |
| Republic of Guinea | 11 | 1.8126(1.5342,2.0918) |  | Republic of Chad | 11 | 1.6517(1.6133,1.6901) |
| Togolese Republic | 12 | 1.6439(1.2561,2.0331) |  | Islamic Republic of Mauritania | 12 | 1.6480(1.6014,1.6947) |
| Republic of Guinea-Bissau | 13 | 1.6310(1.1383,2.1260) |  | Republic of Guinea-Bissau | 13 | 1.6357(1.5932,1.6782) |
| Republic of Guyana | 14 | 1.5485(1.1088,1.9901) |  | Republic of Equatorial Guinea | 14 | 1.6191(1.5680,1.6703) |
| Commonwealth of the Bahamas | 15 | 1.4528(1.0114,1.8962) |  | Republic of Mali | 15 | 1.6078(1.5630,1.6527) |
| Republic of Senegal | 16 | 1.4421(1.0720,1.8137) |  | Republic of Benin | 16 | 1.5315(1.4465,1.6166) |
| Republic of Trinidad and Tobago | 17 | 1.4196(0.8242,2.0186) |  | Kingdom of Morocco | 17 | 1.4990(1.4537,1.5443) |
| Republic of Cameroon | 18 | 1.4184(0.9492,1.8898) |  | Kingdom of Saudi Arabia | 18 | 1.4815(1.3886,1.5745) |
| Republic of the Gambia | 19 | 1.3608(0.9445,1.7787) |  | United States of America | 19 | 1.4607(1.0507,1.8724) |
| Republic of Benin | 20 | 1.3115(1.0157,1.6082) |  | Kingdom of Bhutan | 20 | 1.4520(1.3409,1.5632) |
| Belize | 21 | 1.3059(0.6584,1.9575) |  | Republic of Liberia | 21 | 1.4462(1.2464,1.6464) |
| Republic of Mali | 22 | 1.3052(1.0197,1.5914) |  | State of Eritrea | 22 | 1.3985(1.3397,1.4573) |
| Republic of Paraguay | 23 | 1.2292(0.8907,1.5690) |  | Republic of Senegal | 23 | 1.3694(1.3394,1.3995) |
| United Kingdom of Great Britain and Northern Ireland | 24 | 1.1858(0.4143,1.9631) |  | Republic of Turkey | 24 | 1.3294(1.1850,1.4739) |
| Republic of Côte d'Ivoire | 25 | 1.1677(0.8254,1.5112) |  | Islamic Republic of Pakistan | 25 | 1.3088(1.1996,1.4182) |
| Republic of Indonesia | 26 | 1.0937(0.9730,1.2146) |  | United Republic of Tanzania | 26 | 1.2992(1.2368,1.3616) |
| Saint Vincent and the Grenadines | 27 | 1.0594(0.5020,1.6199) |  | United Mexican States | 27 | 1.2931(1.1137,1.4728) |
| Kingdom of Tonga | 28 | 0.9614(0.7539,1.1694) |  | Canada | 28 | 1.2843(1.2136,1.3550) |
| Sultanate of Oman | 29 | 0.9152(0.2382,1.5968) |  | Republic of Ghana | 29 | 1.2573(1.1687,1.3460) |
| Republic of Cabo Verde | 30 | 0.8640(0.4892,1.2402) |  | Republic of Sierra Leone | 30 | 1.2423(1.2027,1.2819) |
| Republic of Mozambique | 31 | 0.8393(0.6810,0.9979) |  | Slovak Republic | 31 | 1.2165(1.1360,1.2970) |
| Republic of the Niger | 32 | 0.8175(0.4859,1.1502) |  | Arab Republic of Egypt | 32 | 1.2035(1.1343,1.2727) |
| Commonwealth of Dominica | 33 | 0.7891(0.5733,1.0053) |  | Kingdom of Cambodia | 33 | 1.1429(0.9968,1.2892) |
| Saint Lucia | 34 | 0.7880(0.3383,1.2398) |  | Togolese Republic | 34 | 1.1328(1.1050,1.1606) |
| Dominican Republic | 35 | 0.6072(0.2137,1.0023) |  | Republic of Guatemala | 35 | 1.1060(1.0600,1.1520) |
| Republic of Poland | 36 | 0.5912(0.1099,1.0747) |  | Republic of Iraq | 36 | 1.1052(0.9432,1.2675) |
| Antigua and Barbuda | 37 | 0.5595(-0.0754,1.1985) |  | Ireland | 37 | 1.0919(0.9677,1.2164) |
| State of Libya | 38 | 0.5420(0.3245,0.7600) |  | People's Democratic Republic of Algeria | 38 | 1.0912(0.9903,1.1922) |
| Republic of Finland | 39 | 0.5229(-0.1438,1.1940) |  | Republic of the Gambia | 39 | 1.0642(1.0321,1.0964) |
| Republic of Uganda | 40 | 0.5110(0.0715,0.9524) |  | Republic of Madagascar | 40 | 1.0490(0.9741,1.1240) |
| Kingdom of Cambodia | 41 | 0.4961(0.3801,0.6122) |  | Democratic Republic of the Congo | 41 | 1.0490(0.9419,1.1562) |
| Socialist Republic of Viet Nam | 42 | 0.4636(0.3122,0.6151) |  | Lao People's Democratic Republic | 42 | 1.0377(0.9647,1.1108) |
| Democratic People's Republic of Korea | 43 | 0.4607(0.3645,0.5569) |  | Islamic Republic of Afghanistan | 43 | 0.9991(0.8974,1.1010) |
| Republic of Kenya | 44 | 0.4359(0.2246,0.6476) |  | Republic of Costa Rica | 44 | 0.9815(0.8810,1.0822) |
| Islamic Republic of Pakistan | 45 | 0.2078(0.0783,0.3375) |  | Republic of Côte d'Ivoire | 45 | 0.9650(0.9255,1.0045) |
| Federal Democratic Republic of Nepal | 46 | 0.1973(0.0326,0.3623) |  | Republic of Nicaragua | 46 | 0.9376(0.7498,1.1257) |
| Principality of Monaco | 47 | 0.1525(-0.2177,0.5241) |  | Republic of Botswana | 47 | 0.9053(0.7018,1.1091) |
| State of Eritrea | 48 | 0.1497(0.0769,0.2225) |  | Union of the Comoros | 48 | 0.9030(0.8238,0.9823) |
| Republic of Haiti | 49 | 0.1495(-0.0763,0.3758) |  | Islamic Republic of Iran | 49 | 0.8973(0.7832,1.0115) |
| Republic of Yemen | 50 | 0.1426(-0.5551,0.8452) |  | Republic of Yemen | 50 | 0.8960(0.8709,0.9211) |
| Federal Republic of Somalia | 51 | 0.1334(-0.1814,0.4492) |  | State of Kuwait | 51 | 0.8863(0.7866,0.9860) |
| Saint Kitts and Nevis | 52 | 0.1215(-0.4083,0.6541) |  | Federal Democratic Republic of Nepal | 52 | 0.8807(0.8274,0.9339) |
| United Arab Emirates | 53 | 0.0873(-0.3023,0.4784) |  | Republic of Cabo Verde | 53 | 0.8781(0.7268,1.0297) |
| Solomon Islands | 54 | 0.0541(-0.1336,0.2421) |  | Lebanese Republic | 54 | 0.8267(0.6277,1.0260) |
| Republic of South Sudan | 55 | 0.0217(-0.3466,0.3913) |  | Tuvalu | 55 | 0.8262(0.7751,0.8773) |
| Republic of Kiribati | 56 | 0.0017(-0.1736,0.1773) |  | Kingdom of Eswatini | 56 | 0.8143(0.7104,0.9182) |
| Islamic Republic of Mauritania | 57 | 0.0003(-0.2929,0.2944) |  | Republic of Armenia | 57 | 0.8041(0.6707,0.9377) |
| Kingdom of Thailand | 58 | -0.0034(-0.3582,0.3527) |  | Hellenic Republic | 58 | 0.7912(0.5755,1.0074) |
| Bosnia and Herzegovina | 59 | -0.1093(-0.5238,0.3070) |  | Republic of Guyana | 59 | 0.7832(0.7051,0.8614) |
| Kingdom of Bhutan | 60 | -0.1237(-0.2852,0.0381) |  | Republic of Zambia | 60 | 0.7800(0.6931,0.8670) |
| Federated States of Micronesia | 61 | -0.1297(-0.1743, -0.0850) |  | Republic of Indonesia | 61 | 0.7783(0.7568,0.7999) |
| Independent State of Papua New Guinea | 62 | -0.1488(-0.4463,0.1496) |  | Saint Lucia | 62 | 0.7739(0.7117,0.8363) |
| Kingdom of Eswatini | 63 | -0.1959(-0.9340,0.5477) |  | Republic of Vanuatu | 63 | 0.7694(0.7435,0.7953) |
| Taiwan (Province of China) | 64 | -0.1980(-0.7901,0.3977) |  | Palestine | 64 | 0.7606(0.5688,0.9528) |
| Federal Republic of Nigeria | 65 | -0.2044(-0.5254,0.1177) |  | United Arab Emirates | 65 | 0.7592(0.6112,0.9075) |
| Hellenic Republic | 66 | -0.2532(-0.7194,0.2152) |  | Republic of the Union of Myanmar | 66 | 0.7561(0.7011,0.8111) |
| Republic of Nauru | 67 | -0.2607(-0.5012, -0.0195) |  | Republic of Tunisia | 67 | 0.7340(0.6892,0.7788) |
| Republic of Namibia | 68 | -0.2623(-0.4834, -0.0406) |  | Republic of Haiti | 68 | 0.7154(0.6784,0.7524) |
| Republic of Djibouti | 69 | -0.2856(-0.8395,0.2713) |  | Republic of Angola | 69 | 0.7076(0.6485,0.7666) |
| Republic of Suriname | 70 | -0.2945(-0.6146,0.0266) |  | Republic of El Salvador | 70 | 0.7056(0.5975,0.8137) |
| Republic of the Marshall Islands | 71 | -0.3144(-0.5742, -0.0538) |  | Saint Vincent and the Grenadines | 71 | 0.6986(0.6553,0.7419) |
| United Republic of Tanzania | 72 | -0.3204(-0.4275, -0.2133) |  | Republic of Ecuador | 72 | 0.6959(0.6164,0.7754) |
| Tokelau | 73 | -0.3352(-0.6885,0.0193) |  | Federative Republic of Brazil | 73 | 0.6956(0.1991,1.1946) |
| Democratic Republic of the Congo | 74 | -0.3442(-0.5332, -0.1547) |  | Eastern Republic of Uruguay | 74 | 0.6859(0.6195,0.7524) |
| Republic of Vanuatu | 75 | -0.3671(-0.5210, -0.2130) |  | Republic of Poland | 75 | 0.6822(0.5334,0.8312) |
| Republic of the Congo | 76 | -0.3969(-0.6293, -0.1639) |  | Federal Republic of Germany | 76 | 0.6717(0.4280,0.9161) |
| Republic of India | 77 | -0.4159(-0.6852, -0.1457) |  | Greenland | 77 | 0.6713(0.5315,0.8113) |
| Republic of Ghana | 78 | -0.4306(-0.5365, -0.3246) |  | Republic of Kenya | 78 | 0.6682(0.5806,0.7559) |
| Republic of Angola | 79 | -0.4553(-0.6605, -0.2497) |  | Central African Republic | 79 | 0.6651(0.5997,0.7306) |
| Republic of Malta | 80 | -0.4641(-1.0094,0.0843) |  | Democratic Republic of Sao Tome and Principe | 80 | 0.6618(0.5905,0.7331) |
| Republic of the Union of Myanmar | 81 | -0.5075(-0.6628, -0.3518) |  | Republic of Malawi | 81 | 0.6611(0.6177,0.7045) |
| Republic of Malawi | 82 | -0.5103(-0.7531, -0.2670) |  | Republic of Mauritius | 82 | 0.6582(0.5806,0.7359) |
| Republic of Madagascar | 83 | -0.5159(-0.6753, -0.3562) |  | Republic of Cameroon | 83 | 0.6501(0.5667,0.7335) |
| Slovak Republic | 84 | -0.5392(-0.7955, -0.2823) |  | Romania | 84 | 0.6495(0.5427,0.7563) |
| Kingdom of Sweden | 85 | -0.5528(-1.2697,0.1693) |  | Democratic Republic of Timor-Leste | 85 | 0.6491(0.5115,0.7868) |
| Democratic Republic of Timor-Leste | 86 | -0.5618(-1.5141,0.3998) |  | Republic of Paraguay | 86 | 0.6258(0.5835,0.6681) |
| Tuvalu | 87 | -0.5712(-0.6210, -0.5214) |  | Bolivarian Republic of Venezuela | 87 | 0.6258(0.4978,0.7540) |
| Lao People's Democratic Republic | 88 | -0.5714(-0.6361, -0.5066) |  | Republic of Honduras | 88 | 0.6164(0.5759,0.6569) |
| Republic of Niue | 89 | -0.5811(-0.7348, -0.4272) |  | Republic of Austria | 89 | 0.6115(0.5627,0.6604) |
| Montenegro | 90 | -0.5820(-1.1247, -0.0364) |  | Republic of Rwanda | 90 | 0.6089(0.4910,0.7270) |
| Republic of Fiji | 91 | -0.5906(-0.7095, -0.4716) |  | Mongolia | 91 | 0.6060(0.4909,0.7212) |
| Kingdom of the Netherlands | 92 | -0.6339(-0.9130, -0.3540) |  | Republic of Uganda | 92 | 0.6041(0.0974,1.1133) |
| Islamic Republic of Iran | 93 | -0.6427(-0.9558, -0.3286) |  | Republic of Nauru | 93 | 0.5993(0.5619,0.6368) |
| Central African Republic | 94 | -0.6742(-0.7925, -0.5558) |  | Saint Kitts and Nevis | 94 | 0.5964(0.5566,0.6362) |
| Federative Republic of Brazil | 95 | -0.7292(-0.9389, -0.5191) |  | Independent State of Papua New Guinea | 95 | 0.5818(0.5533,0.6103) |
| Republic of Zambia | 96 | -0.7577(-0.9576, -0.5574) |  | Brunei Darussalam | 96 | 0.5747(0.5417,0.6077) |
| Japan | 97 | -0.7712(-1.0528, -0.4888) |  | State of Libya | 97 | 0.5679(0.5197,0.6162) |
| Republic of San Marino | 98 | -0.8002(-0.9434, -0.6567) |  | Republic of Korea | 98 | 0.5443(0.3945,0.6943) |
| Grenada | 99 | -0.8172(-1.4063, -0.2247) |  | Republic of the Philippines | 99 | 0.5433(0.3557,0.7313) |
| Jamaica | 100 | -0.8773(-1.6294, -0.1195) |  | Belize | 100 | 0.5385(0.5162,0.5608) |
| Malaysia | 101 | -0.9062(-1.2061, -0.6055) |  | State of Qatar | 101 | 0.5325(0.4576,0.6074) |
| Republic of Maldives | 102 | -0.9259(-1.1539, -0.6974) |  | Hashemite Kingdom of Jordan | 102 | 0.5281(0.4193,0.6370) |
| Republic of Palau | 103 | -0.9309(-1.1931, -0.6680) |  | Grenada | 103 | 0.5194(0.4752,0.5636) |
| Independent State of Samoa | 104 | -0.9550(-1.1288, -0.7809) |  | Kingdom of Lesotho | 104 | 0.5077(0.4568,0.5586) |
| Palestine | 105 | -0.9830(-1.4275, -0.5364) |  | Republic of Finland | 105 | 0.4979(0.4108,0.5852) |
| North Macedonia | 106 | -0.9918(-1.3255, -0.6569) |  | Georgia | 106 | 0.4952(0.3859,0.6046) |
| Republic of Bulgaria | 107 | -1.0018(-1.4557, -0.5458) |  | Republic of South Africa | 107 | 0.4914(0.4097,0.5731) |
| Northern Mariana Islands | 108 | -1.0216(-1.6472, -0.3920) |  | Republic of Seychelles | 108 | 0.4905(0.4158,0.5652) |
| Bermuda | 109 | -1.0226(-1.6231, -0.4184) |  | Federal Republic of Nigeria | 109 | 0.4892(0.1862,0.7931) |
| Gabonese Republic | 110 | -1.0512(-1.1904, -0.9118) |  | Republic of Fiji | 110 | 0.4870(0.4357,0.5383) |
| Bolivarian Republic of Venezuela | 111 | -1.1084(-1.4967, -0.7187) |  | Commonwealth of Dominica | 111 | 0.4848(0.4339,0.5358) |
| Republic of Tunisia | 112 | -1.1490(-1.2818, -1.0159) |  | New Zealand | 112 | 0.4738(0.4075,0.5402) |
| Republic of Burundi | 113 | -1.1677(-1.2938, -1.0415) |  | Republic of South Sudan | 113 | 0.4677(0.4150,0.5204) |
| State of Israel | 114 | -1.2262(-1.8868, -0.5612) |  | Republic of India | 114 | 0.4668(0.3660,0.5677) |
| Republic of Sudan | 115 | -1.3007(-1.3629, -1.2384) |  | Cook Islands | 115 | 0.4666(0.4041,0.5291) |
| People's Republic of Bangladesh | 116 | -1.3133(-1.4656, -1.1609) |  | Solomon Islands | 116 | 0.4580(0.4457,0.4702) |
| Union of the Comoros | 117 | -1.3279(-2.4727, -0.1697) |  | Republic of Cyprus | 117 | 0.4579(0.3943,0.5216) |
| Australia | 118 | -1.3463(-1.6082, -1.0836) |  | Republic of Panama | 118 | 0.4527(0.4354,0.4699) |
| Republic of the Philippines | 119 | -1.3486(-1.5013, -1.1955) |  | Kingdom of Belgium | 119 | 0.4359(0.3574,0.5144) |
| Republic of Turkey | 120 | -1.3858(-1.5340, -1.2374) |  | Kingdom of Thailand | 120 | 0.4322(0.3304,0.5341) |
| Islamic Republic of Afghanistan | 121 | -1.3919(-1.8352, -0.9467) |  | Republic of Sudan | 121 | 0.4197(0.3294,0.5101) |
| People's Democratic Republic of Algeria | 122 | -1.4242(-1.5684, -1.2797) |  | Republic of Trinidad and Tobago | 122 | 0.4152(0.2696,0.5610) |
| Republic of Honduras | 123 | -1.4331(-1.6727, -1.1929) |  | Syrian Arab Republic | 123 | 0.4126(0.3761,0.4491) |
| Ireland | 124 | -1.4346(-2.3213, -0.5398) |  | People's Republic of Bangladesh | 124 | 0.4001(0.3520,0.4482) |
| Kingdom of Denmark | 125 | -1.4380(-1.7541, -1.1209) |  | Kingdom of Tonga | 125 | 0.3899(0.3515,0.4284) |
| French Republic | 126 | -1.4586(-1.8707, -1.0448) |  | Republic of Namibia | 126 | 0.3886(0.3135,0.4637) |
| Hungary | 127 | -1.4696(-2.0188, -0.9172) |  | Dominican Republic | 127 | 0.3832(0.3630,0.4035) |
| Cook Islands | 128 | -1.4698(-1.6156, -1.3239) |  | Republic of Suriname | 128 | 0.3830(0.3380,0.4280) |
| New Zealand | 129 | -1.4852(-1.9999, -0.9678) |  | Republic of Albania | 129 | 0.3623(0.3379,0.3868) |
| Republic of Botswana | 130 | -1.4858(-1.7402, -1.2308) |  | Jamaica | 130 | 0.3598(0.3167,0.4030) |
| Republic of Equatorial Guinea | 131 | -1.4872(-1.7069, -1.2670) |  | Japan | 131 | 0.3492(0.3018,0.3965) |
| Czech Republic | 132 | -1.4990(-1.8981, -1.0983) |  | Republic of Zimbabwe | 132 | 0.3368(0.2571,0.4165) |
| Republic of Iraq | 133 | -1.4996(-1.7555, -1.2430) |  | Argentine Republic | 133 | 0.3324(0.2064,0.4586) |
| Republic of Cyprus | 134 | -1.5103(-1.8664, -1.1530) |  | Hungary | 134 | 0.3314(0.2562,0.4066) |
| Republic of Iceland | 135 | -1.5521(-2.0347, -1.0671) |  | Swiss Confederation | 135 | 0.2987(0.2099,0.3875) |
| Kingdom of Belgium | 136 | -1.5603(-1.8363, -1.2837) |  | Republic of Colombia | 136 | 0.2978(-0.0220,0.6185) |
| Lebanese Republic | 137 | -1.5690(-1.9823, -1.1539) |  | Plurinational State of Bolivia | 137 | 0.2948(0.2522,0.3373) |
| Republic of Cuba | 138 | -1.5760(-2.3007, -0.8459) |  | Commonwealth of the Bahamas | 138 | 0.2850(0.2044,0.3657) |
| Eastern Republic of Uruguay | 139 | -1.5884(-1.9769, -1.1984) |  | Antigua and Barbuda | 139 | 0.2803(0.2268,0.3338) |
| American Samoa | 140 | -1.6067(-1.9798, -1.2321) |  | Barbados | 140 | 0.2790(0.2328,0.3253) |
| Barbados | 141 | -1.6105(-2.0722, -1.1466) |  | Kingdom of Norway | 141 | 0.2703(0.1839,0.3568) |
| Mongolia | 142 | -1.6960(-2.2216, -1.1675) |  | United States Virgin Islands | 142 | 0.2682(0.2409,0.2956) |
| Turkmenistan | 143 | -1.7075(-2.9212, -0.4786) |  | Socialist Republic of Viet Nam | 143 | 0.2645(0.0948,0.4344) |
| Kingdom of Morocco | 144 | -1.7130(-1.9099, -1.5156) |  | Russian Federation | 144 | 0.2609(-0.0181,0.5408) |
| Principality of Andorra | 145 | -1.7243(-1.9833, -1.4646) |  | Republic of Niue | 145 | 0.2557(0.2175,0.2939) |
| Republic of South Africa | 146 | -1.8974(-2.2938, -1.4995) |  | Australia | 146 | 0.2285(-0.0385,0.4962) |
| Greenland | 147 | -1.9109(-2.1088, -1.7126) |  | Gabonese Republic | 147 | 0.2276(0.2044,0.2508) |
| Republic of Austria | 148 | -1.9664(-2.2433, -1.6887) |  | Republic of Malta | 148 | 0.2241(0.1802,0.2681) |
| Republic of Croatia | 149 | -2.0620(-2.4699, -1.6524) |  | Kingdom of Spain | 149 | 0.2194(0.0544,0.3847) |
| Republic of Albania | 150 | -2.0741(-2.5023, -1.6440) |  | Tokelau | 150 | 0.2129(0.1655,0.2603) |
| Brunei Darussalam | 151 | -2.1239(-2.6244, -1.6209) |  | Republic of Kiribati | 151 | 0.2029(0.1194,0.2864) |
| Republic of Tajikistan | 152 | -2.1411(-2.5472, -1.7333) |  | North Macedonia | 152 | 0.2026(0.0797,0.3257) |
| Republic of Serbia | 153 | -2.1856(-2.3354, -2.0356) |  | Federated States of Micronesia | 153 | 0.1981(0.1752,0.2211) |
| Plurinational State of Bolivia | 154 | -2.2148(-2.2955, -2.1342) |  | Republic of Latvia | 154 | 0.1848(0.0380,0.3318) |
| Republic of Costa Rica | 155 | -2.2554(-2.5971, -1.9125) |  | Republic of Maldives | 155 | 0.1844(0.0951,0.2739) |
| Puerto Rico | 156 | -2.3132(-3.0183, -1.6031) |  | Northern Mariana Islands | 156 | 0.1676(0.0275,0.3079) |
| Republic of Singapore | 157 | -2.3436(-2.8312, -1.8535) |  | Republic of Tajikistan | 157 | 0.1637(0.1107,0.2167) |
| Republic of Nicaragua | 158 | -2.3636(-2.5932, -2.1335) |  | Kingdom of the Netherlands | 158 | 0.1470(0.0582,0.2360) |
| Republic of Slovenia | 159 | -2.3777(-2.8190, -1.9343) |  | Guam | 159 | 0.1446(0.0569,0.2323) |
| Republic of Moldova | 160 | -2.4145(-2.9594, -1.8666) |  | French Republic | 160 | 0.1408(0.0792,0.2024) |
| Republic of Seychelles | 161 | -2.4348(-3.5809, -1.2751) |  | Republic of Palau | 161 | 0.1383(0.1171,0.1596) |
| Federal Republic of Germany | 162 | -2.4416(-2.6981, -2.1844) |  | Republic of Estonia | 162 | 0.1272(-0.0496,0.3044) |
| Argentine Republic | 163 | -2.4708(-2.9993, -1.9394) |  | Republic of Azerbaijan | 163 | 0.1194(0.0704,0.1685) |
| Hashemite Kingdom of Jordan | 164 | -2.5287(-2.7284, -2.3285) |  | Republic of Iceland | 164 | 0.1104(0.0885,0.1324) |
| Republic of Guatemala | 165 | -2.5504(-3.0507, -2.0475) |  | Principality of Monaco | 165 | 0.0871(0.0616,0.1125) |
| Republic of El Salvador | 166 | -2.5827(-3.0079, -2.1557) |  | American Samoa | 166 | 0.0866(0.0520,0.1213) |
| Republic of Italy | 167 | -2.6011(-3.1525, -2.0466) |  | State of Israel | 167 | 0.0713(0.0190,0.1235) |
| People's Republic of China | 168 | -2.8444(-3.2022, -2.4853) |  | Independent State of Samoa | 168 | 0.0434(-0.0277,0.1145) |
| Swiss Confederation | 169 | -2.8462(-3.0079, -2.6842) |  | Democratic People's Republic of Korea | 169 | 0.0158(-0.0056,0.0373) |
| Kingdom of Saudi Arabia | 170 | -2.8781(-3.1888, -2.5665) |  | Montenegro | 170 | -0.0055(-0.0447,0.0338) |
| Federal Democratic Republic of Ethiopia | 171 | -2.9280(-3.2008, -2.6545) |  | Republic of San Marino | 171 | -0.0156(-0.0451,0.0140) |
| Republic of Rwanda | 172 | -2.9447(-3.1621, -2.7268) |  | Turkmenistan | 172 | -0.0183(-0.1123,0.0757) |
| Republic of Azerbaijan | 173 | -2.9689(-3.7024, -2.2299) |  | Republic of Croatia | 173 | -0.0357(-0.1249,0.0536) |
| Syrian Arab Republic | 174 | -3.2523(-3.7037, -2.7988) |  | Grand Duchy of Luxembourg | 174 | -0.0395(-0.0785, -0.0005) |
| Kingdom of Norway | 175 | -3.4324(-3.8503, -3.0127) |  | Kingdom of Bahrain | 175 | -0.0734(-0.1848,0.0381) |
| Republic of Panama | 176 | -3.4923(-3.8304, -3.1531) |  | Principality of Andorra | 176 | -0.0882(-0.1451, -0.0314) |
| Republic of Chile | 177 | -3.5015(-4.0927, -2.9067) |  | Republic of the Marshall Islands | 177 | -0.0980(-0.1768, -0.0192) |
| Georgia | 178 | -3.5338(-4.5820, -2.4740) |  | Republic of the Congo | 178 | -0.1181(-0.2351, -0.0010) |
| Grand Duchy of Luxembourg | 179 | -3.5598(-3.9331, -3.1849) |  | Republic of Uzbekistan | 179 | -0.1288(-0.1765, -0.0812) |
| Romania | 180 | -3.6369(-4.5770, -2.6876) |  | Republic of Moldova | 180 | -0.1431(-0.1779, -0.1083) |
| Portuguese Republic | 181 | -3.7504(-4.1861, -3.3128) |  | Republic of Peru | 181 | -0.1651(-0.2292, -0.1010) |
| State of Qatar | 182 | -3.8158(-4.2850, -3.3442) |  | Ukraine | 182 | -0.1751(-0.3107, -0.0393) |
| Republic of Colombia | 183 | -3.8562(-4.3361, -3.3738) |  | Portuguese Republic | 183 | -0.1806(-0.4536,0.0932) |
| United Mexican States | 184 | -3.8649(-4.1760, -3.5528) |  | Democratic Socialist Republic of Sri Lanka | 184 | -0.1830(-0.2690, -0.0968) |
| Republic of Lithuania | 185 | -4.0093(-4.7185, -3.2949) |  | Czech Republic | 185 | -0.2143(-0.3724, -0.0560) |
| Republic of Peru | 186 | -4.0680(-4.5757, -3.5575) |  | Republic of Belarus | 186 | -0.2150(-0.3832, -0.0465) |
| Republic of Armenia | 187 | -4.2168(-5.3462, -3.0739) |  | Bermuda | 187 | -0.2489(-0.2766, -0.2211) |
| Kingdom of Spain | 188 | -4.3905(-4.7229, -4.0569) |  | Puerto Rico | 188 | -0.2522(-0.3560, -0.1482) |
| Republic of Mauritius | 189 | -4.4940(-5.6448, -3.3291) |  | Kingdom of Denmark | 189 | -0.2705(-0.3258, -0.2152) |
| Republic of Korea | 190 | -4.6214(-5.5054, -3.7290) |  | Republic of Lithuania | 190 | -0.2705(-0.4257, -0.1150) |
| Republic of Ecuador | 191 | -4.9406(-6.0191, -3.8497) |  | Republic of Slovenia | 191 | -0.2743(-0.3771, -0.1714) |
| Republic of Latvia | 192 | -4.9551(-5.5474, -4.3591) |  | United Kingdom of Great Britain and Northern Ireland | 192 | -0.2756(-0.3901, -0.1611) |
| Guam | 193 | -5.0209(-5.4667, -4.5729) |  | Republic of Kazakhstan | 193 | -0.2763(-0.4391, -0.1134) |
| Kyrgyz Republic | 194 | -5.1715(-6.0923, -4.2417) |  | Kingdom of Sweden | 194 | -0.2886(-0.3659, -0.2111) |
| Russian Federation | 195 | -5.2420(-6.9554, -3.4971) |  | Republic of Bulgaria | 195 | -0.3224(-0.3573, -0.2875) |
| Republic of Belarus | 196 | -5.4795(-6.1923, -4.7613) |  | Republic of Serbia | 196 | -0.3289(-0.3757, -0.2821) |
| Republic of Estonia | 197 | -5.7570(-6.8419, -4.6595) |  | Kyrgyz Republic | 197 | -0.3498(-0.4257, -0.2740) |
| Kingdom of Bahrain | 198 | -6.0654(-6.7537, -5.3720) |  | Taiwan (Province of China) | 198 | -0.3588(-0.4249, -0.2927) |
| Democratic Socialist Republic of Sri Lanka | 199 | -7.2004(-8.1565, -6.2344) |  | Republic of Italy | 199 | -0.3663(-0.4768, -0.2556) |
| Republic of Kazakhstan | 200 | -7.4355(-8.7958, -6.0549) |  | Republic of Chile | 200 | -0.4558(-0.7140, -0.1970) |
| Ukraine | 201 | -7.8755(-8.9447, -6.7936) |  | Bosnia and Herzegovina | 201 | -0.6798(-0.7677, -0.5919) |
| State of Kuwait | 202 | -9.4957(-11.4605, -7.4874) |  | Republic of Singapore | 202 | -0.6818(-0.8434, -0.5199) |
| Republic of Uzbekistan | 203 | -9.9587(-11.3263, -8.5699) |  | Republic of Cuba | 203 | -0.7619(-0.9330, -0.5906) |
| Arab Republic of Egypt | 204 | -10.1762(-11.4216, -8.9134) |  | People's Republic of China | 204 | -0.8090(-0.9686, -0.6492) |

CI, confidence interval; DALY, disability-adjusted life year; EAPC, estimated annual percentage change.

Table S6. Deaths, mortality rates, DALYs, and DALY rates of adolescents and young adults with childhood sexual abuse and bullying (1990–2021) by different age groups

| **Age group** | **Death cases (95%UI)** | | **Death rate/100,000 (95%UI)** | | **DALYs (95%UI)** | | **DALY rate/100,000 (95%UI)** | |
| --- | --- | --- | --- | --- | --- | --- | --- | --- |
|  | **1990** | **2021** | **1990** | **2021** | **1990** | **2021** | **1990** | **2021** |
| 10-14 years | 0.96(0.14,2.60) | 0.00(0.00,0.00) | 0.00(0.00,0.00) | 0.00(0.00,0.00) | 548049.26(245933.86,1013221.60) | 841192.32(385273.62,1509495.55) | 102.31(45.91,189.15) | 126.18(57.79,226.43) |
| 15-19 years | 73.38(12.64,169.19) | 47.99(7.35,117.21) | 0.01(0.00,0.03) | 0.01(0.00,0.02) | 941869.30(424757.90,1757484.26) | 1392223.55(665266.38,2524358.39) | 181.33(81.77,338.35) | 223.12(106.62,404.56) |
| 20-24 years | 185.97(33.40,446.84) | 138.68(22.56,347.13) | 0.04(0.01,0.09) | 0.02(0.00,0.06) | 1049560.08(486768.78,1956467.78) | 1453107.01(669293.81,2663157.73) | 213.29(98.92,397.59) | 243.34(112.08,445.97) |

CI, confidence interval; EAPC, estimated annual percentage change; GBD, Global Burden of Disease; SDI, sociodemographic index; UI, uncertainty interval.

**Table S7.** CSA/B-attributable deaths: counts, crude rates, age-standardized mortality rates (per 100,000; 95% UI), and EAPC, 1990 and 2021, by location

| **Location** | **Death cases (95%UI)** | |  | **Mortality rates/100 000(95%UI)** | |  | **EAPC of mortality rates(95%CI)** |  | **Age‑standardised mortality rates/100 000 (95%UI)** | |  | **EAPC of age‑ standardised mortality rates (95%CI)** |  | **Cases change (%, 95%UI)** |
| --- | --- | --- | --- | --- | --- | --- | --- | --- | --- | --- | --- | --- | --- | --- |
|  | 1990 | 2021 |  | 1990 | 2021 |  | 1990-2021 |  | 1990 | 2021 |  | 1990-2021 |  | 1990-2021 |
| **Global** | 260.31(45.95,615.84) | 186.67(33.69,453.54) |  | 0.017(0.003,0.040) | 0.010(0.002,0.024) |  | -2.26(-2.60,-1.92) |  | 0.016(0.003,0.039) | 0.010(0.002,0.024) |  | -2.40(-2.77, -2.02) |  | -28.29(-52.02,12.11) |
| **SDI region** |  |  |  |  |  |  |  |  |  |  |  |  |  |  |
| High SDI | 24.58(4.01,57.13) | 26.88(4.15,62.15) |  | 0.013(0.002,0.029) | 0.014(0.002,0.033) |  | 0.85(0.64,1.07) |  | 0.012(0.002,0.028) | 0.013(0.002,0.031) |  | 0.70(0.49,0.91) |  | 9.35(-17.62,47.59) |
| High-middle SDI | 63.87(10.16,151.26) | 16.42(2.71,38.92) |  | 0.023(0.004,0.053) | 0.007(0.001,0.017) |  | -5.23(-6.42, -4.02) |  | 0.021(0.003,0.050) | 0.007(0.001,0.017) |  | -5.48(-6.66, -4.27) |  | -74.30(-83.56, -61.14) |
| Middle SDI | 97.74(16.52,216.88) | 45.36(7.64,111.72) |  | 0.018(0.003,0.040) | 0.008(0.001,0.020) |  | -2.43(-2.59, -2.28) |  | 0.017(0.003,0.040) | 0.008(0.001,0.020) |  | -2.60(-2.75, -2.45) |  | -53.59(-73.53, -22.73) |
| Low-middle SDI | 63.16(10.45,157.39) | 75.90(11.87,197.63) |  | 0.017(0.003,0.044) | 0.014(0.002,0.036) |  | -0.83(-0.97, -0.68) |  | 0.018(0.003,0.048) | 0.014(0.002,0.036) |  | -1.14(-1.29, -0.98) |  | 20.17(-40.50,141.81) |
| Low SDI | 10.81(1.67,26.25) | 21.99(3.20,51.71) |  | 0.007(0.001,0.017) | 0.006(0.001,0.014) |  | -0.47(-0.63, -0.30) |  | 0.008(0.001,0.020) | 0.006(0.001,0.015) |  | -0.52(-0.69, -0.36) |  | 103.46(13.72,291.13) |
| **GBD region** |  |  |  |  |  |  |  |  |  |  |  |  |  |  |
| Andean Latin America | 1.70(0.20,4.42) | 0.85(0.11,2.36) |  | 0.014(0.002,0.036) | 0.005(0.001,0.014) |  | -3.78(-4.19, -3.36) |  | 0.014(0.002,0.039) | 0.005(0.001,0.014) |  | -4.11(-4.54, -3.69) |  | -49.65(-75.67, -0.20) |
| Australasia | 0.91(0.15,2.06) | 0.55(0.09,1.27) |  | 0.019(0.003,0.043) | 0.010(0.002,0.022) |  | -1.40(-1.69, -1.11) |  | 0.018(0.003,0.041) | 0.009(0.001,0.021) |  | -1.43(-1.69, -1.16) |  | -40.28(-57.85, -8.42) |
| Caribbean | 1.25(0.17,2.76) | 1.53(0.20,3.77) |  | 0.012(0.002,0.026) | 0.014(0.002,0.033) |  | 0.73(0.55,0.91) |  | 0.011(0.001,0.027) | 0.013(0.002,0.031) |  | 0.61(0.42,0.80) |  | 22.63(-22.51,90.58) |
| Central Asia | 8.79(1.40,21.36) | 2.41(0.35,6.21) |  | 0.044(0.007,0.108) | 0.011(0.002,0.028) |  | -5.51(-6.14, -4.89) |  | 0.045(0.006,0.113) | 0.011(0.001,0.029) |  | -5.86(-6.53, -5.18) |  | -72.55(-80.14, -62.03) |
| Central Europe | 3.83(0.55,10.13) | 1.75(0.25,4.38) |  | 0.013(0.002,0.035) | 0.010(0.001,0.024) |  | -0.86(-1.23, -0.49) |  | 0.013(0.002,0.036) | 0.009(0.001,0.024) |  | -1.17(-1.54, -0.80) |  | -54.36(-70.68, -28.69) |
| Central Latin America | 16.54(2.44,39.14) | 8.72(1.17,21.40) |  | 0.030(0.005,0.072) | 0.013(0.002,0.033) |  | -2.77(-3.06, -2.48) |  | 0.032(0.004,0.077) | 0.013(0.002,0.033) |  | -3.04(-3.30, -2.78) |  | -47.26(-69.24, -11.01) |
| Central Sub-Saharan Africa | 1.72(0.26,4.46) | 3.83(0.50,9.60) |  | 0.010(0.001,0.026) | 0.009(0.001,0.021) |  | -0.42(-0.54, -0.31) |  | 0.011(0.001,0.028) | 0.009(0.001,0.025) |  | -0.42(-0.54, -0.30) |  | 122.32(24.37,307.70) |
| East Asia | 78.08(13.04,179.20) | 19.84(2.95,54.57) |  | 0.021(0.004,0.048) | 0.008(0.001,0.022) |  | -2.80(-3.15, -2.45) |  | 0.018(0.003,0.045) | 0.008(0.001,0.025) |  | -2.82(-3.15, -2.50) |  | -74.59(-89.66, -45.28) |
| Eastern Europe | 36.58(5.87,87.59) | 7.99(1.17,19.17) |  | 0.077(0.012,0.185) | 0.024(0.004,0.058) |  | -5.61(-7.13, -4.08) |  | 0.076(0.011,0.199) | 0.025(0.003,0.062) |  | -5.99(-7.35, -4.61) |  | -78.17(-86.62, -63.84) |
| Eastern Sub-Saharan Africa | 1.82(0.23,4.15) | 3.54(0.51,8.33) |  | 0.003(0.000,0.007) | 0.002(0.000,0.006) |  | -0.94(-1.07, -0.81) |  | 0.003(0.000,0.008) | 0.003(0.000,0.006) |  | -1.05(-1.15, -0.94) |  | 94.78(43.14,252.13) |
| High-income Asia Pacific | 3.50(0.54,8.69) | 0.82(0.11,1.98) |  | 0.008(0.001,0.021) | 0.003(0.000,0.008) |  | -3.40(-4.01, -2.79) |  | 0.008(0.001,0.021) | 0.003(0.000,0.007) |  | -3.39(-3.98, -2.78) |  | -76.68(-85.68, -59.14) |
| High-income North America | 9.77(1.54,23.95) | 19.56(2.93,44.89) |  | 0.016(0.003,0.039) | 0.027(0.004,0.063) |  | 2.50(2.10,2.91) |  | 0.015(0.002,0.038) | 0.026(0.004,0.061) |  | 2.38(1.97,2.79) |  | 100.10(30.47,227.80) |
| North Africa and Middle East | 2.10(0.34,5.12) | 1.89(0.28,4.63) |  | 0.002(0.000,0.005) | 0.001(0.000,0.003) |  | -1.75(-1.89, -1.62) |  | 0.002(0.000,0.005) | 0.001(0.000,0.003) |  | -2.02(-2.15, -1.89) |  | -9.94(-35.61,31.04) |
| Oceania | 0.13(0.01,0.38) | 0.24(0.03,0.66) |  | 0.006(0.001,0.018) | 0.006(0.001,0.016) |  | -0.25(-0.48, -0.01) |  | 0.007(0.001,0.019) | 0.006(0.001,0.019) |  | -0.41(-0.62, -0.19) |  | 84.08(-21.21,269.88) |
| South Asia | 52.91(7.39,148.88) | 71.47(8.55,205.10) |  | 0.016(0.002,0.045) | 0.014(0.002,0.039) |  | -0.38(-0.64, -0.11) |  | 0.017(0.002,0.047) | 0.013(0.001,0.038) |  | -0.74(-1.01, -0.46) |  | 35.08(-51.43,267.91) |
| Southeast Asia | 21.54(2.72,56.01) | 21.51(2.59,67.66) |  | 0.015(0.002,0.038) | 0.013(0.002,0.040) |  | -0.85(-1.05, -0.65) |  | 0.015(0.002,0.043) | 0.012(0.001,0.037) |  | -1.08(-1.29, -0.87) |  | -0.15(-58.17,136.49) |
| Southern Latin America | 0.80(0.11,1.93) | 0.42(0.06,1.06) |  | 0.006(0.001,0.015) | 0.003(0.000,0.007) |  | -2.70(-3.21, -2.20) |  | 0.006(0.001,0.016) | 0.003(0.000,0.007) |  | -2.99(-3.50, -2.48) |  | -47.25(-71.07, -4.08) |
| Southern Sub-Saharan Africa | 0.98(0.15,2.41) | 0.84(0.13,2.09) |  | 0.006(0.001,0.014) | 0.004(0.001,0.010) |  | -1.62(-2.01, -1.22) |  | 0.006(0.001,0.015) | 0.004(0.001,0.010) |  | -1.92(-2.31, -1.53) |  | -14.72(-54.77,56.60) |
| Tropical Latin America | 6.65(0.95,16.70) | 5.27(0.70,13.09) |  | 0.014(0.002,0.035) | 0.010(0.001,0.026) |  | -0.74(-0.94, -0.53) |  | 0.014(0.002,0.040) | 0.010(0.001,0.025) |  | -1.15(-1.35, -0.96) |  | -20.77(-62.38,61.23) |
| Western Europe | 7.70(1.25,17.15) | 4.68(0.75,10.74) |  | 0.009(0.002,0.021) | 0.006(0.001,0.015) |  | -1.17(-1.52, -0.81) |  | 0.008(0.001,0.019) | 0.006(0.001,0.014) |  | -1.04(-1.42, -0.67) |  | -39.25(-57.65, -10.45) |
| Western Sub-Saharan Africa | 2.99(0.46,7.19) | 8.96(1.40,20.87) |  | 0.005(0.001,0.012) | 0.006(0.001,0.013) |  | 0.35(0.05,0.64) |  | 0.005(0.001,0.013) | 0.006(0.001,0.014) |  | 0.40(0.14,0.66) |  | 199.96(73.51,467.79) |

AYA adolescents and young adults, CSA/B Childhood sexual abuse and bullying, CI confidence interval, EAPC estimated annual percentage change, GBD Global Burden of Disease, SDI sociodemographic index, UI uncertainty interval.

**Table S8.** CSA/B-attributable DALYs: counts, crude rates, age-standardized DALY rates (per 100,000; 95% UI), and EAPC, 1990 and 2021, by location

| **Location** | **DALY cases (95%UI)** | |  | **DALY rates/100 000(95%UI)** | |  | **EAPC of DALY rates(95%CI)** |  | **Age‑standardised DALY rates/100 000 (95%UI)** | |  | **EAPC of age‑ standardised DALY rates (95%CI)** |  | **Cases change (%, 95%UI)** |
| --- | --- | --- | --- | --- | --- | --- | --- | --- | --- | --- | --- | --- | --- | --- |
|  | **1990** | **2021** |  | **1990** | **2021** |  | **1990-2021** |  | **1990** | **2021** |  | **1990-2021** |  | **1992-2021** |
| **Global** | 2539478.64(1188028.88,4668588.62) | 3686522.88(1762134.17,6574720.99) |  | 164.13(76.79,301.75) | 195.28(93.34,348.28) |  | 0.58(0.51,0.64) |  | 162.94(74.25,303.28) | 194.65(90.81,353.57) |  | 0.54(0.48,0.61) |  | 45.17(37.07,55.15) |
| **SDI region** |  |  |  |  |  |  |  |  |  |  |  |  |  |  |
| High-middle SDI | 437300.46(211460.77,787490.89) | 560207.65(280455.87,972500.62) |  | 223.27(107.97,402.07) | 301.88(151.13,524.05) |  | 1.10(0.92,1.29) |  | 217.56(104.37,393.01) | 296.33(145.38,521.54) |  | 1.09(0.90,1.28) |  | 28.11(20.04,40.15) |
| High SDI | 489543.72(225494.62,911395.83) | 418134.62(194819.22,766898.80) |  | 172.51(79.46,321.16) | 185.11(86.25,339.52) |  | 0.22(0.13,0.31) |  | 169.09(77.24,315.51) | 184.27(85.23,339.75) |  | 0.23(0.15,0.32) |  | -14.59(-22.24, -4.49) |
| Low-middle SDI | 825834.07(376810.33,1536430.78) | 922148.57(426155.97,1721678.27) |  | 150.47(68.66,279.95) | 166.83(77.10,311.48) |  | 0.31(0.22,0.39) |  | 148.51(66.34,278.96) | 166.03(74.81,312.87) |  | 0.27(0.18,0.36) |  | 11.66(4.68,18.50) |
| Low SDI | 581789.83(272416.18,1059877.63) | 1128937.32(552467.13,1968846.11) |  | 160.84(75.31,293.02) | 204.24(99.95,356.19) |  | 0.79(0.75,0.83) |  | 164.22(74.28,300.85) | 203.17(96.71,357.81) |  | 0.68(0.64,0.71) |  | 94.05(78.79,112.94) |
| Middle SDI | 203058.96(97710.65,370479.48) | 654586.21(307701.77,1186254.11) |  | 130.45(62.77,238.00) | 177.19(83.29,321.12) |  | 1.01(0.99,1.03) |  | 135.11(61.65,251.52) | 180.30(83.53,331.91) |  | 0.96(0.94,0.98) |  | 222.36(205.46,239.96) |
| **GBD region** |  |  |  |  |  |  |  |  |  |  |  |  |  |  |
| Andean Latin America | 17203.44(7380.05,33629.37) | 25766.77(11331.81,51091.43) |  | 139.75(59.95,273.18) | 149.25(65.64,295.94) |  | 0.21(0.19,0.22) |  | 141.49(59.34,284.02) | 147.28(63.31,294.02) |  | 0.11(0.10,0.12) |  | 49.78(36.70,63.84) |
| Australasia | 14088.30(6665.62,25466.20) | 17523.55(8643.57,30820.61) |  | 292.83(138.55,529.32) | 305.44(150.66,537.21) |  | 0.28(0.06,0.49) |  | 284.09(133.21,514.78) | 302.12(143.05,548.45) |  | 0.30(0.10,0.49) |  | 24.38(10.28,39.99) |
| Caribbean | 18506.29(8529.21,35827.34) | 20129.93(9250.12,38540.46) |  | 173.30(79.87,335.50) | 177.71(81.66,340.24) |  | 0.12(0.05,0.19) |  | 170.56(76.22,330.83) | 174.76(78.11,337.32) |  | 0.08(0.04,0.13) |  | 8.77(1.39,16.45) |
| Central Asia | 12088.52(5631.51,22991.47) | 12870.65(5792.32,24716.53) |  | 60.96(28.40,115.94) | 58.17(26.18,111.70) |  | -0.10(-0.16, -0.05) |  | 61.30(28.06,118.95) | 58.65(25.59,114.84) |  | -0.28(-0.34, -0.22) |  | 6.47(-7.58,17.17) |
| Central Europe | 31352.50(13950.83,61280.56) | 20656.70(9534.03,38810.95) |  | 107.38(47.78,209.88) | 113.90(52.57,214.00) |  | 0.21(0.16,0.27) |  | 107.55(46.71,210.52) | 113.01(51.54,212.49) |  | 0.16(0.13,0.18) |  | -34.11(-40.13, -25.04) |
| Central Latin America | 70054.48(31592.44,132677.54) | 111377.95(51238.66,207583.76) |  | 129.12(58.23,244.54) | 171.26(78.79,319.20) |  | 0.95(0.79,1.11) |  | 130.41(57.50,250.45) | 168.82(75.28,317.25) |  | 0.87(0.70,1.03) |  | 58.99(47.90,72.42) |
| Central Sub-Saharan Africa | 38145.13(17436.40,71666.23) | 127563.69(60249.21,231389.84) |  | 220.39(100.74,414.07) | 283.83(134.05,514.84) |  | 0.88(0.80,0.96) |  | 227.13(101.64,431.27) | 292.61(133.66,542.33) |  | 0.86(0.78,0.95) |  | 234.42(206.96,267.06) |
| East Asia | 548117.10(252375.69,1024490.13) | 299907.83(129839.37,576175.76) |  | 147.27(67.81,275.27) | 123.41(53.43,237.10) |  | -0.77(-0.92, -0.62) |  | 142.28(64.05,269.10) | 123.75(53.02,242.17) |  | -0.68(-0.85, -0.52) |  | -45.28(-54.83, -38.24) |
| Eastern Europe | 84217.11(39391.22,152993.48) | 61627.62(28413.27,113035.61) |  | 178.32(83.41,323.94) | 186.78(86.11,342.59) |  | 0.09(-0.14,0.31) |  | 177.17(81.71,328.05) | 189.24(84.83,354.22) |  | -0.01(-0.16,0.14) |  | -26.82(-33.68, -21.99) |
| Eastern Sub-Saharan Africa | 83074.84(39587.79,153813.03) | 275078.91(129298.91,500438.92) |  | 133.92(63.82,247.95) | 189.14(88.90,344.09) |  | 1.12(1.08,1.17) |  | 139.15(62.39,261.65) | 192.50(88.81,352.97) |  | 1.08(1.05,1.11) |  | 231.12(209.32,254.42) |
| High-income Asia Pacific | 43992.86(20741.48,84113.60) | 30035.14(14064.78,56831.23) |  | 104.43(49.23,199.66) | 115.06(53.88,217.70) |  | 0.43(0.36,0.51) |  | 101.77(46.38,192.93) | 111.69(51.67,210.98) |  | 0.45(0.37,0.54) |  | -31.73(-37.30, -25.26) |
| High-income North America | 159696.02(76252.85,283434.86) | 289290.31(146006.28,493571.10) |  | 261.06(124.65,463.33) | 405.85(204.84,692.45) |  | 1.45(1.07,1.84) |  | 253.33(122.28,448.82) | 396.70(197.62,683.39) |  | 1.44(1.04,1.84) |  | 81.15(64.37,102.03) |
| North Africa and Middle East | 285753.25(128092.61,525537.69) | 551137.45(268496.99,956031.76) |  | 262.39(117.62,482.56) | 339.59(165.44,589.06) |  | 0.93(0.87,0.99) |  | 264.01(114.80,490.60) | 340.17(159.74,597.44) |  | 0.90(0.86,0.95) |  | 92.87(73.93,126.21) |
| Oceania | 2944.45(1226.57,5823.95) | 6291.71(2735.35,12412.51) |  | 140.75(58.63,278.40) | 156.03(67.83,307.82) |  | 0.40(0.38,0.42) |  | 142.27(57.97,278.76) | 156.61(65.23,305.57) |  | 0.36(0.34,0.38) |  | 113.68(95.88,133.45) |
| South Asia | 525912.26(250967.99,951255.16) | 959783.18(459312.75,1692241.70) |  | 157.24(75.03,284.41) | 182.51(87.34,321.80) |  | 0.54(0.47,0.62) |  | 160.48(74.08,291.26) | 179.76(85.50,320.90) |  | 0.38(0.31,0.45) |  | 82.50(67.21,98.58) |
| Southeast Asia | 146556.75(65085.12,284650.97) | 204431.27(89118.21,386594.32) |  | 98.78(43.87,191.86) | 119.54(52.11,226.07) |  | 0.73(0.68,0.78) |  | 99.24(42.87,193.00) | 118.40(50.64,228.57) |  | 0.68(0.62,0.73) |  | 39.49(31.97,48.27) |
| Southern Latin America | 37029.25(17811.88,66679.63) | 43412.00(21299.78,75180.79) |  | 279.75(134.56,503.75) | 283.06(138.88,490.21) |  | -0.01(-0.19,0.17) |  | 280.27(130.03,517.90) | 278.13(132.87,488.56) |  | -0.09(-0.28,0.09) |  | 17.24(-1.36,38.61) |
| Southern Sub-Saharan Africa | 33595.76(15310.03,62850.55) | 47920.83(22420.48,86681.67) |  | 196.65(89.62,367.90) | 219.67(102.78,397.36) |  | 0.48(0.40,0.56) |  | 198.68(88.66,369.34) | 220.10(102.67,399.11) |  | 0.40(0.35,0.45) |  | 42.64(34.53,52.78) |
| Tropical Latin America | 70753.14(32180.51,135255.56) | 97209.48(42932.37,192920.35) |  | 147.83(67.24,282.61) | 192.20(84.89,381.44) |  | 0.69(0.20,1.17) |  | 149.48(66.49,283.51) | 186.85(81.15,366.45) |  | 0.53(0.06,1.01) |  | 37.39(20.50,54.29) |
| Western Europe | 243600.15(117490.18,446920.75) | 222018.49(112589.14,395461.01) |  | 296.35(142.93,543.70) | 308.04(156.21,548.68) |  | 0.21(0.15,0.26) |  | 289.40(138.84,528.47) | 305.17(149.75,538.60) |  | 0.23(0.17,0.28) |  | -8.86(-18.58,4.26) |
| Western Sub-Saharan Africa | 72797.05(35175.92,129897.36) | 262489.43(127945.20,469104.85) |  | 121.65(58.78,217.06) | 162.67(79.29,290.71) |  | 0.94(0.80,1.08) |  | 124.58(57.70,227.98) | 165.88(78.22,305.81) |  | 0.94(0.81,1.06) |  | 260.58(231.15,293.97) |

AYA adolescents and young adults, CSA/B Childhood sexual abuse and bullying, CI confidence interval, EAPC estimated annual percentage change, GBD Global Burden of Disease, SDI sociodemographic index, UI uncertainty interval.

Note: For CSA/B, DALYs ≈ YLD; attributable YLL is minimal because the included outcomes (major depressive, anxiety, alcohol-use disorders) are non-fatal in the GBD framework.

Figure S1. Comparison of childhood sexual abuse and bullying-related mortality (a) and DALY rates (b) in the 21 regions in 1990 and 2021.


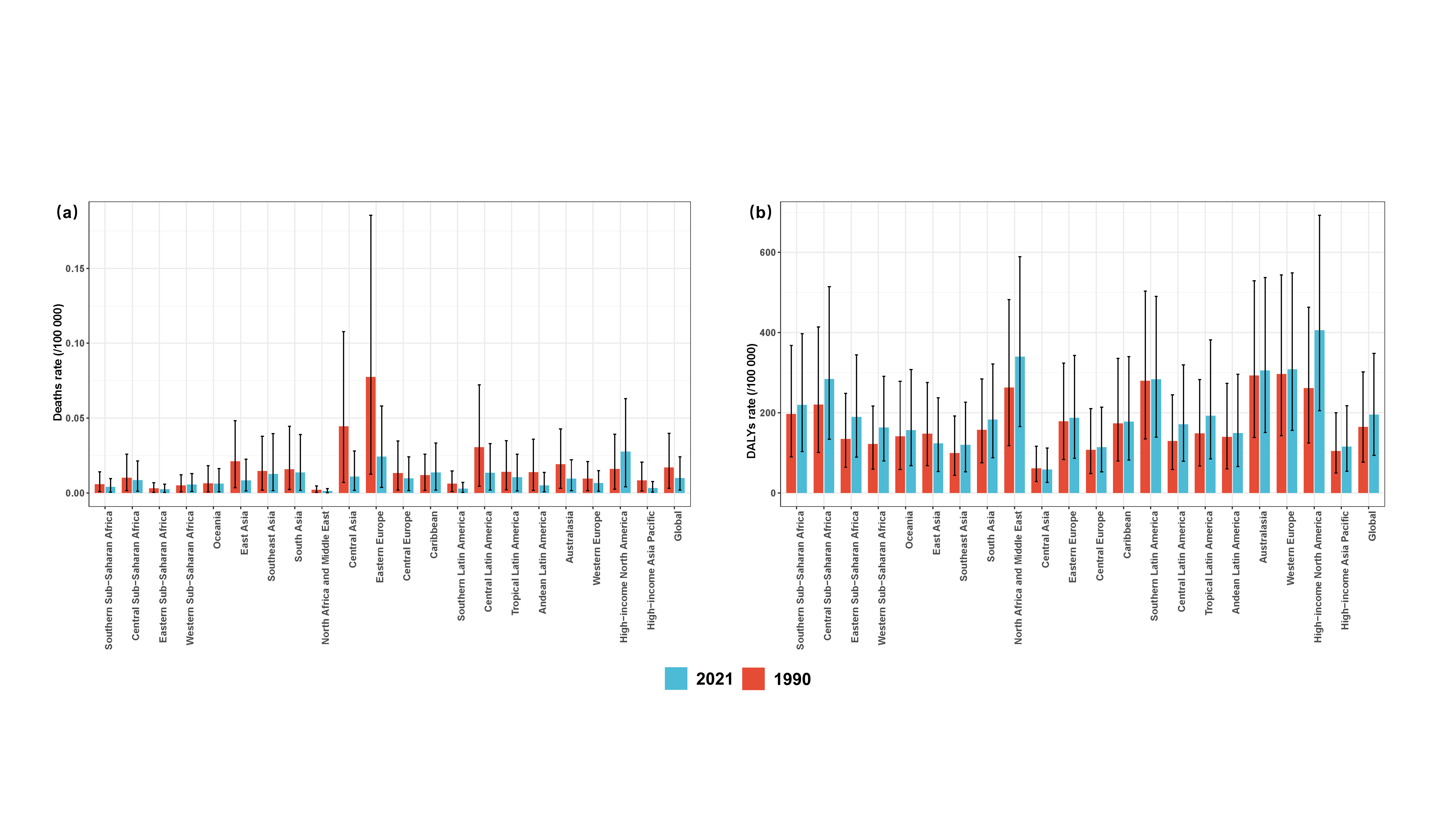


DALYs, disability-adjusted life years; SDI, sociodemographic index.

Figure S2. Proportion of childhood sexual abuse and bullying-related disease burden by different age groups


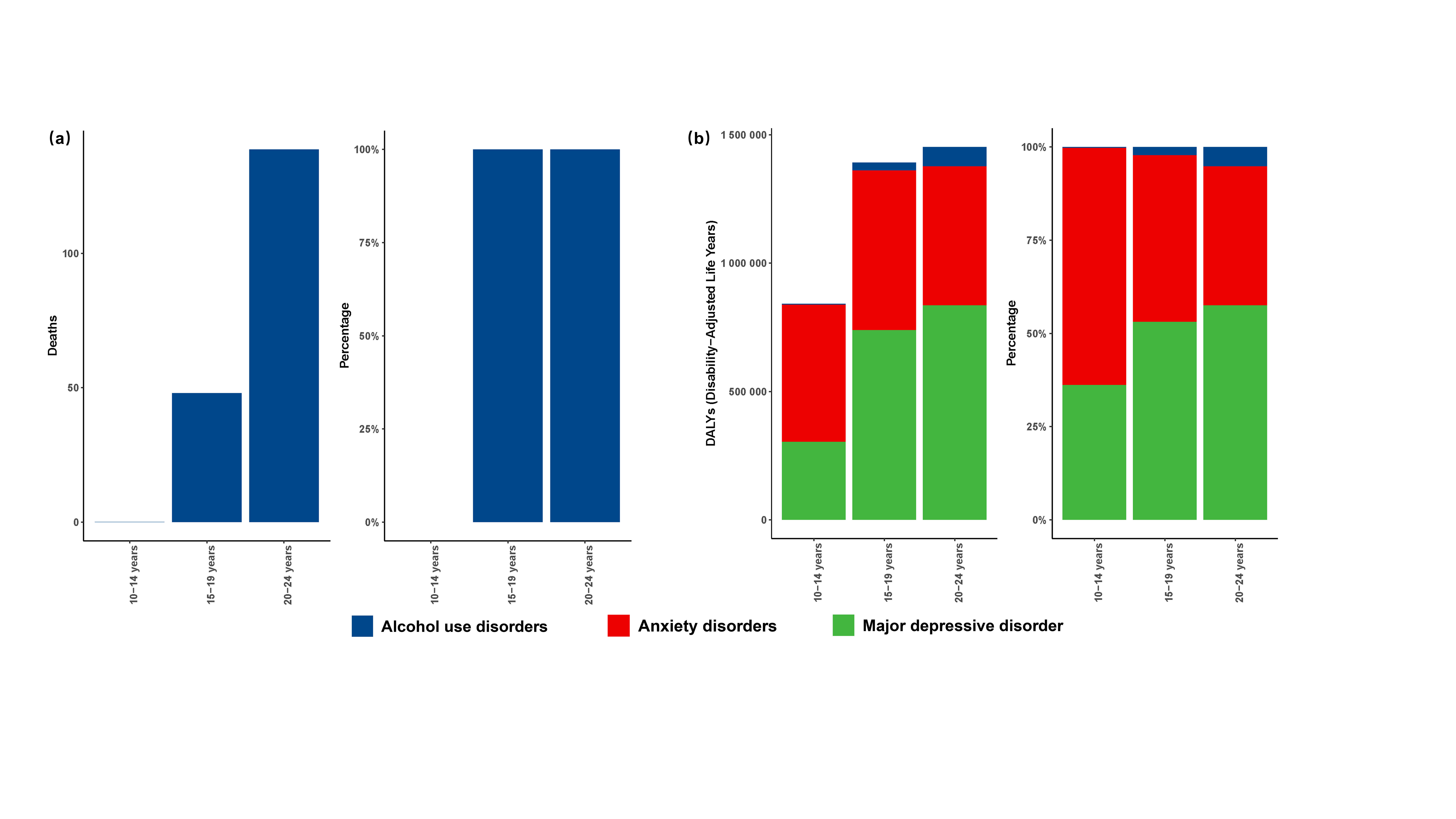


DALYs, disability-adjusted life years.
